# Reaction Temperature and Solvent Influence Reactivity Ratios in the Copolymerization of Ethylene Oxide and Propylene Oxide

**DOI:** 10.1021/acs.macromol.5c02759

**Published:** 2025-12-12

**Authors:** Milena S. Hesse, Gregor M. Linden, Holger Frey

**Affiliations:** Department of Chemistry, 9182 Johannes Gutenberg University Mainz, Duesbergweg 10-14, 55128 Mainz, Germany

## Abstract

Statistical copolymers of ethylene oxide (EO) and propylene oxide (PO) are widely used in industry and academia. Despite their decade-long use, the influence of the polymerization conditions on reactivity ratios is underexplored, and surprisingly solution and bulk properties of the resulting polyether copolymers have not been reported in a systematic manner. In this study we examined the copolymerization of EO and PO in a variety of solvents (dimethyl sulfoxide, toluene, anisole) and at different temperatures (25–60 °C), correlating reaction conditions with the thermal and solubility properties of the resulting P­(EO-*co*-PO) copolymers. The copolymerization was monitored online by *in situ* ^1^H NMR spectroscopy to determine the reactivity ratios for the full conversion range. The results show a temperature-dependent trend in reactivity ratios (*r*) for different solvents. In toluene, the reactivity ratios converge with increasing temperature, changing from *r*_PO_ = 0.26 and *r*_EO_ = 3.78 at 40 °C to *r*_PO_ = 0.31 and *r*_EO_ = 3.21 at 60 °C. A similar pattern is observed in anisole, with the reactivity ratios shifting from *r*_PO_ = 0.28 and *r*_EO_ = 3.52 at 40 °C to *r*_PO_ = 0.30 and *r*_EO_ = 3.32 at 60 °C, respectively. In contrast, the reactivity ratios in DMSO are generally slightly more similar, with *r*_PO_ = 0.32 and *r*_EO_ = 3.10 at 40 °C. Thermal characterization of the polyether copolymers revealed similar melting points of approximately 10 °C and enthalpies of around 40 J·g^–1^. Cloud point measurements of the copolymers showed decreased aqueous solubility as the differences in reactivity ratios decreased. These findings demonstrate that the statistical EO/PO copolymerization reaction conditions affect the gradient and thereby significantly influence copolymer physical properties, highlighting the need to consider these parameters for applications.

## Graphical abstract

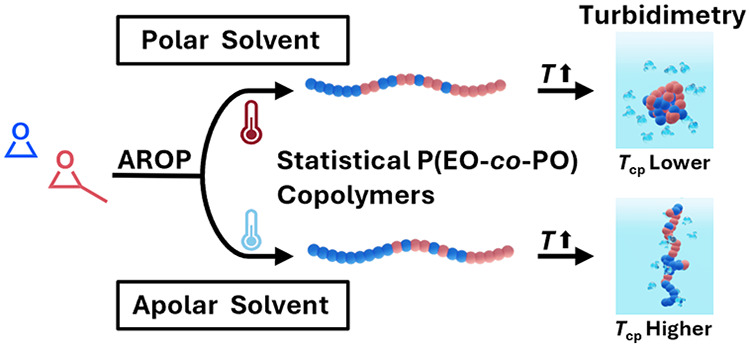

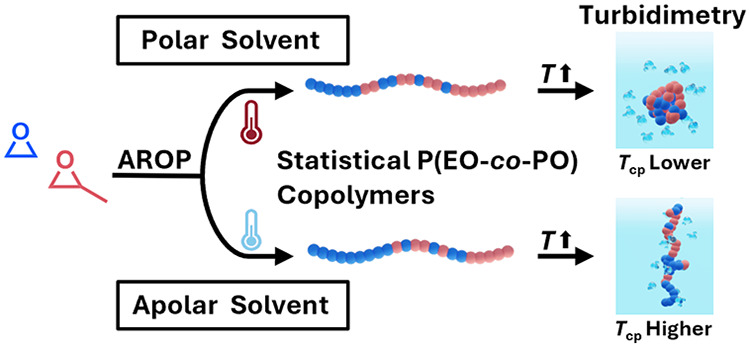

## Introduction

Statistical polyether copolymers of the type poly­(ethylene oxide-*co*-propylene oxide) (P­(EO-*co*-PO)) are a crucially important class of materials.^1^ They are commonly produced by copolymerizing ethylene oxide (EO) with propylene oxide (PO), enabling tailoring the properties of poly­(ethylene oxide) (PEO), often aiming at reducing or fully avoiding crystallization. On the other hand, introducing a certain amount of EO in PPO structures can be used to increase the polarity of polyols.^2–5^ The resulting polyether copolymers are widely used for the large-scale industrial production of polar polyether polyols in polyurethane (PU) manufacturing,^6,7^ while also being tailored for various applications such as surfactants for drug delivery, tissue engineering, and biomolecule delivery.^8–11^ In PU foam fabrication, P­(EO-*co*-PO) copolymers with moderate hydrophilicity act as intrinsic surfactants, enhancing compatibility with water used as a blowing agent. This results in the formation of a highly uniform cellular structure. P­(EO-*co*-PO) copolymers with a high EO content enable the production of flexible and soft polyurethane foams without the need for auxiliary blowing agents, such as dichloromethane. However, incorporating more than 25% PO is essential to prevent the EO-rich segments from forming undesired crystalline domains.^12^ P­(EO-*co*-PO) copolymers exhibit a lower critical solution temperature (LCST), which allows them to be dissolved in aqueous solutions at lower temperatures, while undergoing phase separation at higher temperatures, particularly at the cloud point temperature (*T*_cp_).^13^ At temperatures above *T*_cp_, the copolymer chains aggregate due to inter- and intramolecular interactions. This transition is thermodynamically driven by unfavorable entropy of mixing. This allows for numerous applications that require precise control over release mechanisms and selective separations in aqueous environments.^8,10^ The adaptation of the thermoresponsive behavior of P­(EO-*co*-PO) copolymers is closely linked to their microstructure, which determines the lower critical solution temperature (LCST).^8^ The distribution of EO and PO units within the copolymer chains significantly influences both thermal properties and the interaction with water.^14^ Deliberate tuning of the monomer gradients is employed both for polyols and for foam stabilizers.^12^

Early fundamental studies, e.g., by Bailey and Callard in 1959, have shown that the statistical copolymerization of EO and PO allows for tunable LCSTs that can be adjusted depending on the ratio of these two monomers.^2,15^ Further studies by Tjerneld et al. afforded the temperature versus copolymer concentration phase diagram of P­(EO_45_-*co*-PO_34_) based on the Flory–Huggins theory of polymer solubility.^11,13^ More recent studies, for instance by Persson et al., investigated the phase behavior of copolymers with different PO contents and provided information on how changes in composition affect the cloud point temperature.^11^ In addition, Louai et al. studied the influence of salt addition on the solution properties of P­(EO-*co*-PO).^16^ Overall, this research illustrates the crucial role of microstructural control in optimizing the thermoresponsive features of P­(EO-*co*-PO) copolymers for a wide range of practical applications.^2^

Various studies investigated the kinetics of EO/PO copolymerization, focusing on the resulting copolymer microstructure. This is represented by the reactivity ratios (*r*_PO_ = *k*_PO,PO_/*k*_PO,EO_; *r*_EO_ = *k*_EO,EO_/*k*_EO,PO_) with the rate constants *k* for homo- and cross propagation, respectively. In the widely employed anionic ring-opening copolymerization (AROP), EO reacts faster than PO, which contains a methyl group attached to the epoxide moiety. In 1991, Heatley et al. reviewed the available literature on the reactivity ratios for the EO/PO comonomer pair, which varied widely from *r*_EO_ = 1.34, *r*_PO_ = 0.14 to *r*_EO_ = 6.5, *r*_PO_ = 1.49. However, in some cases, details of the reaction conditions were not reported.^17–22^ Furthermore, the calculation methods used are considered outdated and not recommended anymore.^23^ Heatley et al. investigated the copolymerization in bulk and analyzed the results using the Mayo–Lewis equation,^24^ obtaining reactivity ratios of *r*_EO_ = 2.8 and *r*_PO_ = 0.25. Although the reactivity ratios were stated as being independent of temperature, this could imply only slight variations.^17^ Three years later, in 1994, Holmberg et al. reported reactivity ratios of *r*_EO_ = 1.8 and *r*_PO_ = 0.3 for copolymerization in *N*,*N*-dimethylformamide (DMF) at 90 °C,^25^ using the Fineman–Ross method for data analysis.^26^ Santacesaria et al. investigated the bulk copolymerization between 100 and 130 °C in 1996, reporting reactivity ratios of *r*_EO_ = 4.8–2.5 and *r*_PO_ = 0.22–0.17, depending on the temperature.^27^ Applying the related monomer-activated anionic ring-opening polymerization (MAROP) further increases this reactivity difference (*r*_PO_ = 0.16, *r*_EO_ = 6.4).^5^ In contrast, copolymerizations employing the industrially established double metal cyanide (DMC) catalysis^28^ reverse these reactivity ratios, with PO becoming the more reactive monomer (*r*_PO_ = 2.4, *r*_EO_ = 0.42).^5^

To the best of our knowledge, no conclusive study presents the influence of solvents and temperatures on the reactivity ratios of the EO/PO comonomer pair in AROP. In industrial syntheses, P­(EO-*co*-PO) are commonly prepared solvent-free, i.e., in bulk or by using the DMC catalyst.^12^ No conclusive study has investigated the influence of solvent or temperature on the reactivity ratios for this comonomer pair. In other studies, the copolymerization of EO and glycidyl ethers have been reported. Reactivity ratios of EO and allyl glycidyl ether (AGE) vary with the choice of solvent. Copolymerization in dimethyl sulfoxide (DMSO) yields ratios of *r*_EO_ = 0.92 and *r*_AGE_ = 1.08, while THF gives *r*_EO_ = 0.78 and *r*_AGE_ = 1.29. For EO and ethoxy vinyl glycidyl ether (EVGE), the difference in reactivity ratios is even more pronounced, leading to a soft gradient structure.^29^ A similar trend is observed for the copolymerization of EO with glycidyl methyl ether (GME). In DMSO, the copolymerization yields random copolymers (*r*_EO_ ≈ *r*_GME_ ≈ 1),^30^ whereas slight gradient structures are procured in the more apolar solvents toluene and anisole.^31^

AROP is typically carried out in polar, aprotic solvents like tetrahydrofuran (THF), DMSO, or hexamethylphosphoric triamide (HMPTA).^12^ Polymerizations in DMSO proceed at a high rate,^32,33^ albeit its high boiling point (189 °C) makes complete solvent removal challenging. Toluene is less polar and has been used occasionally, despite the low polymerization rate of epoxides in this solvent.^34,35^ Anisole, with a lower boiling point of 154 °C, is considered a green solvent alternative.^36,37^

A critical issue in the AROP of substituted epoxides such as PO and glycidyl ethers is the occurrence of undesirable chain transfer reactions. Proton abstraction from the methyl or methylene group of the epoxide moiety generates an allyl alkoxide, which can act as an initiator. This process limits the achievable molar mass and increases the dispersity of the resulting polymer.^28,38–40^ Low temperature, a moderate degree of deprotonation, a low monomer-to-initiator ratio, and use of a suitable solvent can decrease it.^12^ Allgaier et al. suppressed the transfer reaction by adding [18]­crown-6 in toluene while maintaining a reasonable polymerization rate.^41^

This study aims at elucidating the copolymerization kinetics of EO and PO under varied reaction conditions, using the solvents DMSO, anisole, and toluene at different temperatures. Copolymers with comparable molar masses and comonomer composition were synthesized to assess (i) the nature of the gradient formed and (ii) the extent of chain transfer under the chosen reaction conditions. Since there is a lack of systematic data on the physical properties of P­(EO-*co*-PO) copolymers in literature, aqueous solubility and thermal properties in solution and in bulk have been analyzed in a detailed manner for the series of copolymers.

## Experimental Section

Detailed information regarding reagents, polymer synthesis, and the characterization techniques employed, is available in the Supporting Information (SI).

## Results and Discussion

The influence of the copolymerization conditions on the microstructure of statistical P­(EO-*co*-PO) copolymers has been investigated. To this end, multiple copolymerizations were conducted in sealable NMR tubes, measuring *in situ* ^1^H NMR kinetics. Copolymers were synthesized under corresponding copolymerization conditions in anionic flasks in an additional series of experiments to investigate how variations of the microstructure impact the physical properties of the copolymers. The frequently employed initiator alcohol 2-(benzyloxy)­ethanol was partially deprotonated and used for the ^1^H NMR kinetics measurements, as it provides an integrable benzyl group in the ^1^H NMR and exhibits good solubility in all studied solvents.^42–44^ Partially deprotonated triethylene glycol monomethyl ether was employed for the synthesis of the P­(EO-*co*-PO) copolymers to reduce the impact of the initiator on the physical properties, as it comprises three EO repeating units (Scheme 1).

**Scheme 1:**
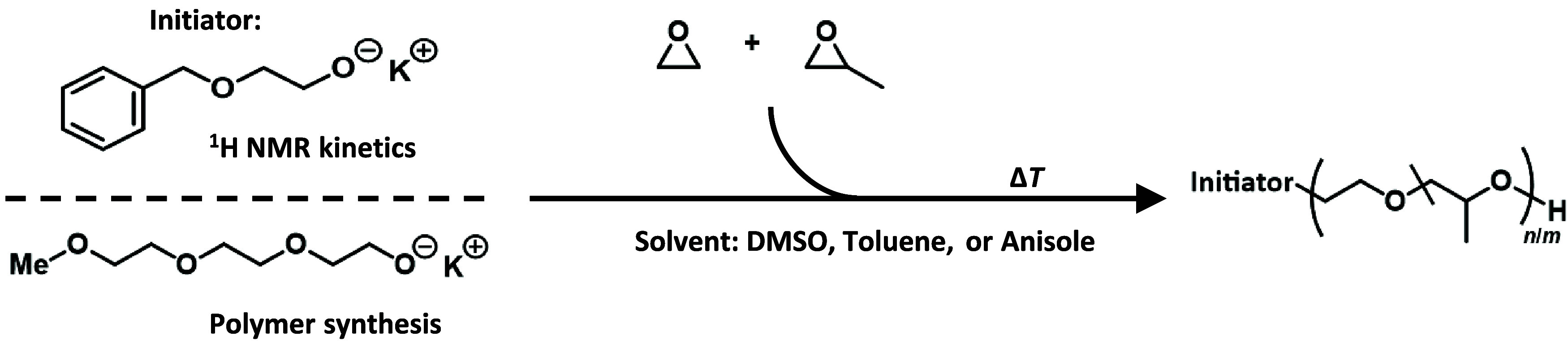
Synthesis of EO/PO Copolymers[Fn s1fn1]

a
^1^H NMR kinetics were conducted using 2-(benzyloxy)-ethanolate as an initiator, while monomethyl triethylene glycolate was employed for copolymer synthesis in reaction vessels. The termination step is omitted for clarity reasons.

To investigate the copolymerization kinetics *in situ*, the polymerizations were conducted within an NMR tube. The initiator salt, potassium 2-(benzyloxy)­ethanolate, was synthesized by heating KO^*t*^Bu with 2-(benzyloxy)­ethanol overnight under azeotropic distillation in vacuo using a Schlenk flask. The resulting salt was then dissolved in the different, predried solvents employed for polymerization (DMSO-*d*_6_, toluene-*d*_8_, anisole) and transferred to the NMR tube. Dried and freshly distilled PO was also transferred into the NMR tube under an argon counterflow. EO was condensed into the tube under vacuum at −78 °C, achieving the targeted comonomer ratio EO/PO with respect to the initiator. Subsequently, the NMR tube was sealed and transferred to the NMR spectrometer, where it was heated to the respective polymerization temperature (25, 40, 50, 60 °C). The entire polymerization process was monitored by acquiring ^1^H NMR spectra, usually every 2 min throughout the reaction. To separate the effects of solvent and temperature on the polymerization process, all other parameters were maintained constant. The degree of deprotonation (base equivalents per hydroxyl group of the initiator) was set to 0.45 for all reactions to ensure a homogeneous solution. All copolymers obtained from the kinetics measurements were characterized by size exclusion chromatography (SEC) and showed monomodal molar mass distributions with a dispersity *Đ* of <1.1. The respective SECs can be found in the SI (Figures S67–S78).

Experimental details can be found in the SI. An example of the stacked spectra is shown in Figure 1, demonstrating the time-dependent decrease of the signals of both monomers, which was used for evaluation of the copolymerization kinetics. The resulting time–conversion plots and individual versus total monomer conversion can be found in the SI (Figures S1–S59).

**Figure 1:**
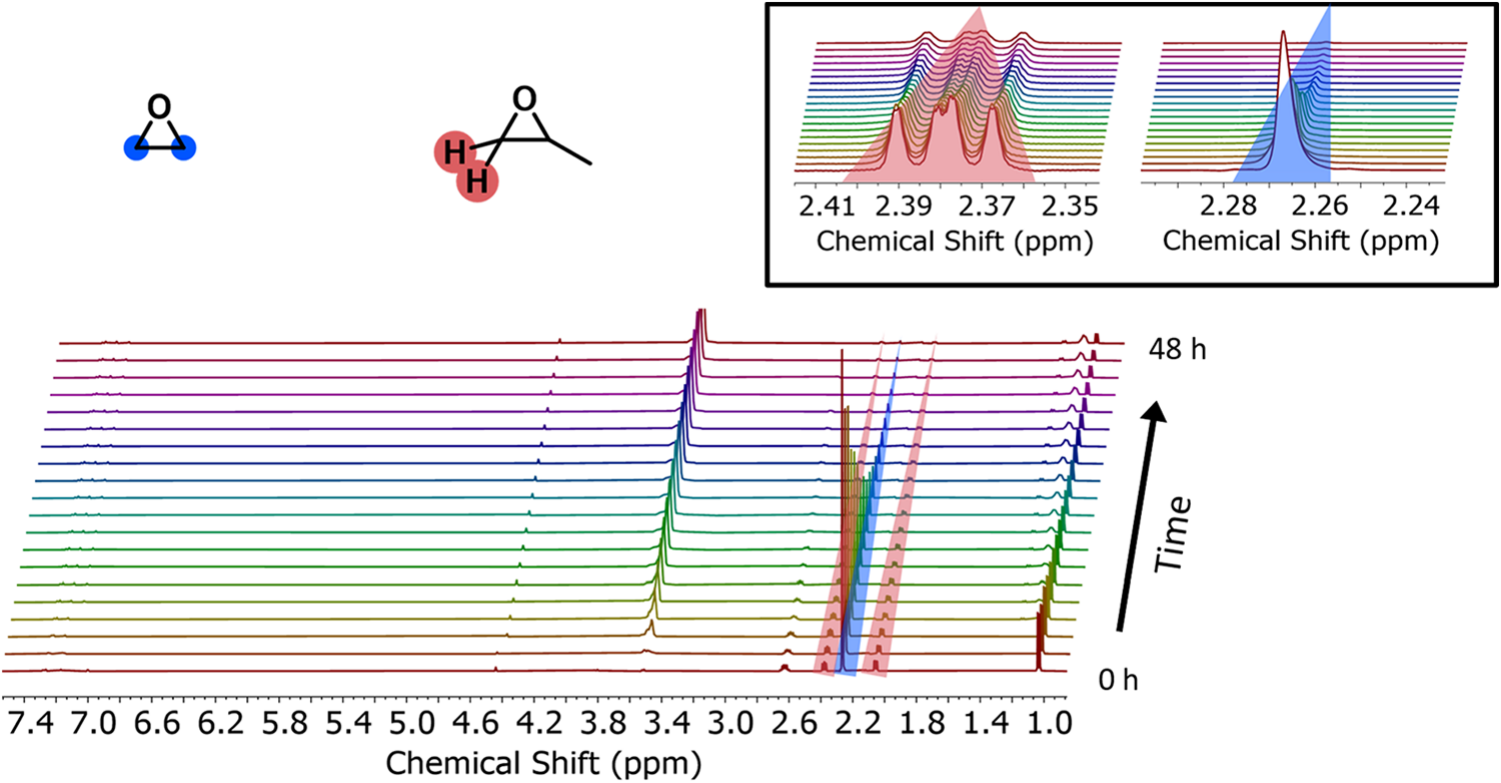
Stacked ^1^H NMR spectra of the copolymerization of EO and PO. Zoom-in shows the decrease of the PO (red) and EO monomer signals (blue). Polymerization temperature 60 °C, solvent: toluene-*d*_8_, 400 MHz. Since spectra were acquired every 2 min, only every 75th spectrum is displayed.

### Comparison of the Reaction Rates

To compare the solvents in terms of reaction rate, pseudo-first-order plots of the online ^1^H NMR kinetics measurements were created (see Figures S60–S66). The apparent rate constant of EO (*k*_app, copo_ (EO)) was determined from the decrease of the monomer signals with time. The following eq 1 leads to the pseudo-first-order plot and the slope yields *k*_app_. It indicates how quickly the reaction progresses. This value was used as a proxy for comparing the reaction conditions.

1 $$\ln\left(\frac{{\left[M\right]}_{0}}{{\left[M\right]}_{t}}\right)=k_{\mathrm{app}}\cdot t$$

Both monomers exhibited linear conversion behavior in DMSO, whereas an induction period was observed for anisole and toluene at all temperatures. Similar behavior was observed in THF for the polymerization of EO in the presence of Li counterions and a phosphazene base by Müller et al.^45^ This induction period is attributed to the so-called “crown ether effect”:^46–48^ after the addition of the first 5–6 monomer units, the slope becomes linear. The potassium ions are more effectively solvated by the growing polyether chain, enhancing charge separation between the ions and the active alkoxide chain end (Figure 2).

**Figure 2:**
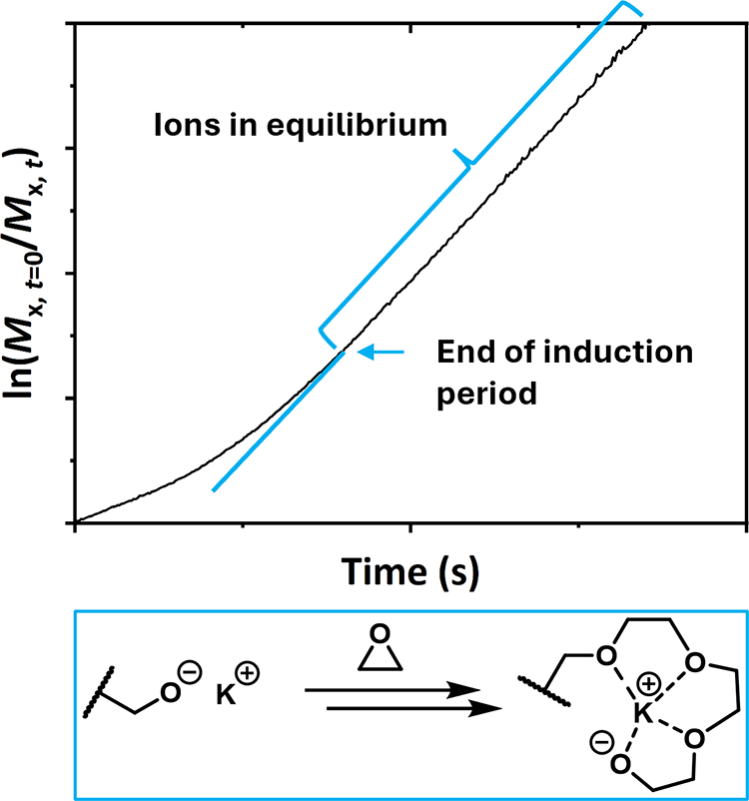
Exemplary pseudo-first-order plot with a visible induction period. The turquoise elongation shows the graph without the induction period. Lower Scheme: Solvation of the counterion (potassium) by the growing polyether chain, referred to as the “Weibull-Törnquist effect”.^49–51^ Reprinted and adapted from *Reference Module in Chemistry* 2016, Penczek, S.; Pretula, J.B., *Ring-Opening Polymerization*, Page 23, Copyright 2016, with permission from Elsevier.^48^

The experiments involving crown ether addition in anisole and toluene displayed linear behavior from the beginning, like that observed in DMSO. However, the reactions in anisole and toluene showed a much smaller slope and consequently a lower reaction rate. Figure 3 highlights the induction period in toluene-*d*_8_, clearly showing the starting phase. Alkoxides are known to aggregate even in polar solvents like THF, and their propagation follows fractional-order kinetics depending on the active chain end concentration. Kazanskii et al. observed that potassium and cesium alkoxides formed trimers, while sodium alkoxides formed tetramers.^52,53^ At the beginning of the reaction, the growing polyether chain complexes with the counterion, shifting the equilibrium from contact ion pairs to solvent-separated ion pairs. This leads to an increase in the propagation rate constant. An increased degree of ion pair dissociation toward free ions is possible.^54^ Therefore, the observed induction period in solvents that are less polar than THF, such as anisole and toluene, is expected. The induction period is not pronounced in our study, as we used a degree of deprotonation of 45%. The aggregation of the potassium alkoxides was already diminished, due to fast proton transfer between alcohols and alkoxides.^28,55,56^ This finding is confirmed by the linear behavior of the polymerization with the addition of [18]­crown-6 (Figure 3, turquois graph). The crown ether complexes the potassium cation, preventing alkoxide aggregation and significantly accelerating the reaction. This enhancement goes beyond the typical “crown ether effect”. We would like to emphasize that all the reactions were first order with respect to monomer after the induction period, as can be seen in Figures S61, S62, and S65.

**Figure 3:**
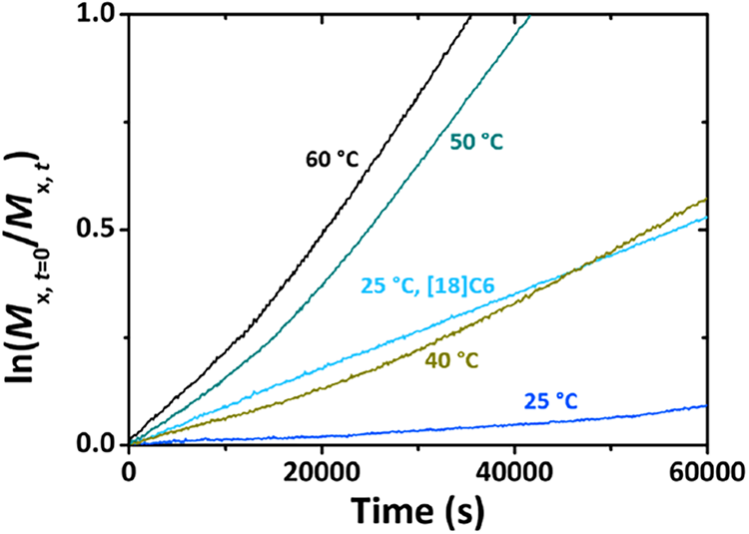
Pseudo-first-order plot of the copolymerization of EO with PO in toluene-*d*_8_ at different conditions obtained by ^1^H NMR kinetics. The enlarged starting period shows the induction of the EO signal. The graphs for PO are omitted for clarity reasons.

The linear regime in the pseudo-first-order plots was analyzed using a linear fit to compare the reaction rates at different conditions. We would also like to emphasize that *k*_app, copo_(EO) is not equal to the propagation constant of EO in homopolymerization but works as a proxy to compare the different conditions. The results are visualized in Figure 4, while the values can be found in Table S1.

**Figure 4:**
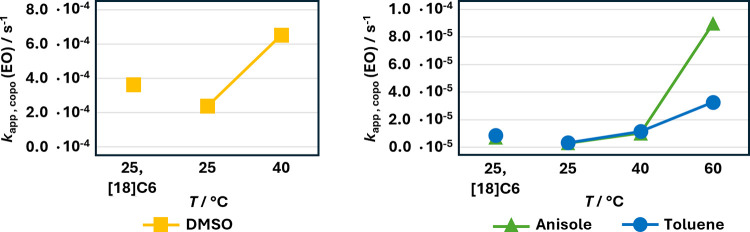
Comparison of the apparent propagation rate constant of EO in the copolymerization with PO. Left: DMSO-*d*_6_. Right: Comparison of toluene-*d*_8_ and anisole. Please note that this does not equal the propagation rate constant *k*_p_ of a homopolymerization.

In DMSO, [18]­crown-6 at 25 °C leads to a significant increase in the reaction rate, despite the already high polarity of the solvent (dielectric constant ε = 47.13).^57^ However, the increase occurs only by a factor of ≈1.5. In contrast, raising the temperature to 40 °C results in a 2.8-fold increase. The reaction rate in toluene (ε = 2.408),^58^ and anisole (ε = 4.3724)^59^ is similarly influenced by adding crown ether at 25 °C. The change in reaction rate with increasing temperature remains consistent until the temperature reaches 60 °C, at which point the reaction rate in anisole becomes higher (2.8-fold) compared to copolymerization in toluene at 60 °C. Kinetic studies in DMSO at 60 °C were omitted, as the AROP of PO under these conditions exhibits extensive chain transfer to monomer, rendering the system unsuitable for accurate determination of reaction rates and reactivity ratios.

### Influence of the Polymerization Conditions on Chain Transfer

A major challenge in the AROP of substituted epoxides is the occurrence of undesirable chain transfer reactions. Proton abstraction from the α-methyl or methylene group of the epoxide moiety leads to the formation of an allyl alkoxide that can act as a new initiator (Scheme 2).^28,38–40^ Chain transfer of alkoxides to monomer impacts the polymerization outcomes by limiting the achievable molar mass, increasing dispersity, and the formation of undesired allylic end groups. Understanding the extent of chain transfer is important with respect to the physical properties and microstructural control of P­(EO-*co*-PO) explored in this study.^28^ Chain transfer reduces the degree of deprotonation, slowing the reaction, and producing undesired allylic chain ends.^18,60–62^ As demonstrated in previous studies, several reaction parameters are known to increase the abundance of proton abstraction from the PO monomer. These parameters include a high degree of deprotonation,^63^ a small size of the counterion (Li^+^ > Na^+^ > K^+^ > Cs^+^),^28,64^ and high target molar masses. Conversely, the addition of crown ether has been shown to reduce the formation of allyl species.^65,66^ It has also been demonstrated that the solvent has an impact on this transfer reaction.^67,68^

**Scheme 2:**
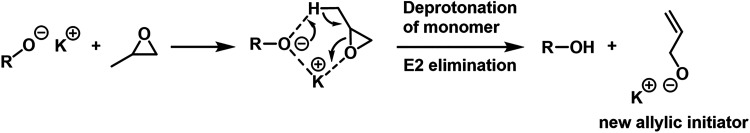
Transfer Reaction of an Active Chain End to the Monomer PO, Resulting in a New Allylic Initiator and a Lower Degree of Deprotonation^a^

a Subsequent isomerization of the double bond to the vinyl species is omitted. Adapted and reprinted from Grobelny et al.,^63^ Wiley © 2016.

Therefore, we also examined the transfer reactions occurring during the copolymerization in our selected solvents (DMSO, anisole, and toluene) and the extent of proton abstraction. The extent of proton abstraction was quantified by assessing the ratio of the integral of allylic protons to the integral of protons from the initiator, 2-(benzyloxy)­ethanol (exemplarily shown in Figure S80). The results are summarized in Table 1.

**Table 1:** Fraction χ of Allylic Species from Active Chain End Transfer to PO at Different Reaction Conditions in an EO/PO Mixture of 40/4 equiv

solvent	*T*/°C	χ allylic species/%
DMSO-*d*_6_	25^a^	7
DMSO-*d*_6_	25	7
DMSO-*d*_6_	40	6
anisole	25^a^	0
anisole	25	0
anisole	40	1
anisole	60	3
toluene-*d*_8_	25^a^	0
toluene-*d*_8_	25	0
toluene-*d*_8_	40	2
toluene-*d*_8_	50	2
toluene-*d*_8_	60	2

a 2 equiv [18]­crown-6 per potassium.

Our study shows that the polar solvent DMSO-*d*_6_ leads to a higher extent of proton abstraction from the PO monomer, consistent with previous findings.^69,70^ Boileau stated that DMSO is known to be excellent for proton abstraction reactions and exhibits low propagation-to-transfer constant ratios.^47^ At 25 and 40 °C, DMSO-*d*_6_ exhibits a stable presence of allylic species (6–7%). This trend underscores the impact of solvent polarity, as polar environments increase the deprotonation potential and transfer reaction abundance.^67^ In contrast, the results for the apolar solvents anisole and toluene-*d*_8_ demonstrate a significantly lower abundance of allylic species. Notably, at 25 °C, no allylic chain ends were detected in anisole or toluene-*d*_8_, strongly indicating that apolar environments suppress proton abstraction. It should be noted that this result applies only to the specific conditions chosen for this study (*T* = 25 °C, EO/PO/I = 40/4/1, [M] = 7 mol/L, [I] = 0.16 mol/L, degree of deprotonation = 45%) and can therefore only be meaningfully compared with polymerizations conducted under identical conditions. However, even at 60 °C, the occurrence of allyl groups remains relatively low in toluene-*d*_8_ (2%) and anisole (3%). These findings highlight that solvent polarity plays a critical role for chain transfer reactions and the formation of allylic chain ends. The apolar solvents anisole and toluene show a significant reduction compared to the polar DMSO. Nevertheless, it should be noted that this positive effect is also accompanied by a significant reduction in polymerization rate. Consequently, depending on the primary focus of the synthesis, apolar solvents may not always be the preferred choice.

Moreover, the data reveal that the addition of crown ether in DMSO-*d*_6_ did not produce the expected reduction in allylic species. Across the experiments conducted at 25 and 40 °C, the observed levels of allylic chain ends remained consistent at 6–7%, indicating that the mitigating effect of crown ether was not as pronounced in DMSO as anticipated. This result suggests that while crown ether is effective at reducing allyl end group formation in apolar solvents by complexing with the counterions and reducing deprotonation, its efficacy in polar solvents like DMSO may be limited.^65^ The data imply that the strong solvation and high polarity of DMSO may overshadow the counterion complexation effect of crown ether, resulting in similar levels of proton abstraction and allylic chain formation, regardless of crown ether addition.

### Reactivity Ratios of EO/PO by *In Situ* ^1^H NMR Copolymerization Kinetics

The highly established homopolymers PEO and PPO exhibit completely different properties. Poly­(ethylene oxide) (PEO) is crystalline and highly hydrophilic, while poly­(propylene oxide) (PPO) structures are amorphous and hydrophobic. Copolymerization of these two monomers affords materials that combine both sets of characteristics, with the specific balance influenced by both the monomer composition due to the polymerization method as shown in previous works.^5,14^ This highlights the critical role of copolymer microstructure in determining the physical properties of the respective copolymer. Thus, gaining a deeper understanding of how fundamental parameters such as polymerization temperature and solvent impact the microstructure is highly relevant. To this end, online kinetics measurements have been conducted to investigate these effects.

Several methods are available to calculate the reactivity ratios from the monomer conversion.^5,26,71–73^ Among those, Beckingham et al. recommended using integrated models over differential models, as they propose greater accuracy.^23^ Furthermore, nonterminal or chain end independent models should be preferred over terminal models if they describe the data with sufficient precision.^5,72^ This principle of relying on the simplest explanation of the data is termed “Ockham′s Razor”.^74^ We compared the data from the online kinetics measurements by applying a terminal model of Meyer and Lowry^73^ and a nonterminal or “ideal” model from Jaacks,^75,76^ both of which are integrated methods. Since the integrated nonterminal BSL model^72^ delivered the very same results as the Jaacks model, we decided to omit these results. Equation 2 includes the respective monomer concentration at time *t* ([M_*x*_]_*t*_) and the initial concentration ([M_*x*_]_0_). The Jaacks fit is plotted as follows.

2 $$\log\left(\frac{{{[M}_{1}]}_{t}}{{{[M}_{1}]}_{0}}\right)=r_{1}\cdot \log\left(\frac{{{[M}_{2}]}_{t}}{{{[M}_{2}]}_{0}}\right)$$

The slope is used to derive *r*_1_. Given the relations *r*_1_·*r*_2_ = 1 (because of the nonterminal nature of the model) and *r*_2_ = 1/*r*_1_ both reactivity ratios can be obtained. The Meyer-Lowry fit is shown in the SI (eq S2), together with both plotted graphs and the simulated composition plots (Figures S1–S59). The results of the kinetics experiments are summarized in Table 2. The Jaacks model affords conclusive reactivity ratios throughout all experiments, with coefficients of determination *R* ^2^ > 0.99 in all cases except for toluene at 25 °C, due to the relatively noisy signal, as shown in Figure S36. The Meyer-Lowry model also gives reasonable results, showing good agreement with the Jaacks model in DMSO. However, for anisole and toluene, the reactivity ratios diverge significantly and show higher errors. In the case of copolymerization in toluene at 25 °C, the fit resulted in no reasonable solution, indicating the weakness of the terminal model toward noise, which leads to overfitting.^5,77^ Since the Jaacks model provided consistent results for all solvents, we focus on this model to explain the data.

**Table 2:** Summarized Results of the Reactivity Ratios Obtained under Different Conditions

		Jaacks			Meyer-Lowry		
solvent	*T*/°C	*r*_PO_	*r*_EO_	*R* ^2^	*r*_PO_	*r*_EO_	NormRes
DMSO-*d*_6_	25^a^	0.32 ± 0.01	3.15 ± 0.01	0.999	0.31 ± 0.01	2.97 ± 0.06	0.006
DMSO-*d*_6_	25	0.31 ± 0.01	3.25 ± 0.01	0.999	0.30 ± 0.01	3.11 ± 0.03	0.004
DMSO-*d*_6_	40	0.32 ± 0.01	3.10 ± 0.01	0.999	0.33 ± 0.01	3.21 ± 0.09	0.001
anisole	25^a^	0.31 ± 0.01	3.26 ± 0.01	0.991	0.83 ± 0.37	5.25 ± 1.43	0.021
anisole	25	0.28 ± 0.01	3.54 ± 0.13	0.995	0.22 ± 0.16	2.87 ± 1.72	0.006
anisole	40	0.28 ± 0.01	3.52 ± 0.01	0.998	0.39 ± 0.01	4.38 ± 0.08	0.037
anisole	60	0.30 ± 0.01	3.32 ± 0.01	0.991	0.32 ± 0.01	3.92 ± 0.14	0.137
toluene-*d*_8_	25^a^	0.33 ± 0.01	3.05 ± 0.01	0.990	0.85 ± 0.10	5.87 ± 0.50	0.062
toluene-*d*_8_	25	0.29 ± 0.01	3.49 ± 0.02	0.958	^b^	^b^	^b^
toluene-*d*_8_	40	0.26 ± 0.01	3.78 ± 0.01	0.999	0.33 ± 0.01	4.59 ± 0.02	0.028
toluene-*d*_8_	50	0.28 ± 0.01	3.62 ± 0.01	0.998	0.33 ± 0.01	4.54 ± 0.01	0.015
toluene-*d*_8_	60	0.31 ± 0.01	3.21 ± 0.01	0.999	0.34 ± 0.01	3.64 ± 0.02	0.029

a 2 equiv [18]­crown-6 per potassium.

b Fit did not result in a reasonable solution.

The kinetics could not be conducted for DMSO at 60 °C due to considerable chain transfer to the monomer at this temperature. The reactivity ratios indicate a gradient structure between the two monomers in all cases. The expected instantaneous composition of an equimolar copolymer is given in Figure 5 as an example. All other composition plots can be found in the SI. At low conversions, the chains mainly incorporate EO, while at high conversions almost only PO is added.

**Figure 5:**
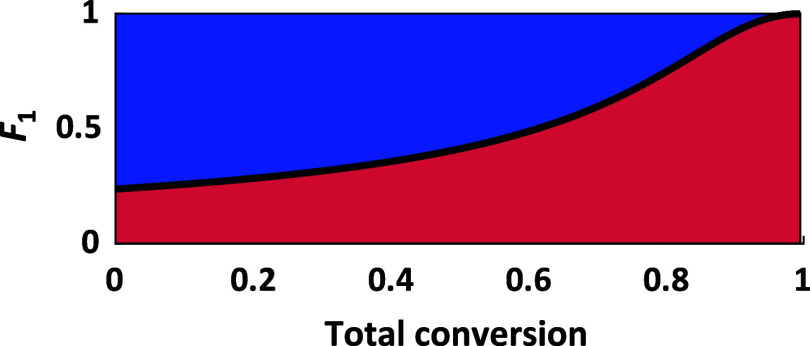
Composition plot of the *in situ* ^1^H NMR copolymerization kinetics study of EO (blue) and PO (red) with a hypothetic equimolar monomer ratio (Solvent: toluene-*d*_8_, 60 °C) showing *r*_PO_ = 0.31, *r*_EO_ = 3.21.

### Effect of [18]­crown-6 on the Reactivity Ratios

Crown ethers are known to accelerate the AROP of epoxides, since they complex the respective counterion, enhancing the reactivity of the alkoxide chain end. For long-chain epoxide monomers, the addition of crown ether often represents a key step to enable polymerization.^78^ Since potassium was used as a counterion in the AROP, [18]­crown-6 was added to the EO/PO copolymerization. The effect of crown ether addition on the copolymerization and specifically the reactivity ratios was investigated at 25 °C. The crown ether caused the reactivity ratios to converge slightly in all cases. Deviations from the values without crown ether are more pronounced for *r*_EO_, due to its larger numerical value, making changes in its reactivity ratio *r*_EO_ more visible. The change observed for the polar solvent DMSO is rather small when comparing the experiment with and without crown ether addition (3%). In anisole, a much less polar solvent than DMSO, the reactivity ratio *r*_EO_ decreases by 8% with the addition of crown ether, equaling *r*_EO_ in DMSO without crown ether. In toluene, the effect is similar, but slightly larger, with a 13% decrease. The addition of crown ether accelerates the reaction rate but has only a minor impact on the reactivity ratios in apolar solvents, with an even smaller effect observed in DMSO.

### Effect of the Temperature on the Reactivity Ratios

The increase in temperature to 40 °C in DMSO results in a more pronounced decrease of *r*_EO_ (*r*_EO_ = 3.10) compared to the addition of crown ether at 25 °C (*r*_EO_ = 3.15). This suggests that the elevated temperature increases the reactivity of the chain end, reducing the disparity between the reactivity of the two monomers. This conclusion is supported by the higher reaction rate, as discussed before. In anisole, *r*_EO_ is similar at 25 and 40 °C but decreases at 60 °C, suggesting it passes through a maximum. A similar pattern is observed in toluene, where *r*_EO_ peaks at 40 °C and decreases even more significantly than in anisole at 60 °C. The intermediate step in toluene at 50 °C underlines this gradual decrease as this bridges *r*_EO_ between 40 and 60 °C. A similar trend was observed for the bulk copolymerization of EO and PO between 70 and 120 °C. The reactivity ratios shifted from *r*_EO_ = 3.0, *r*_PO_ = 0.17 to *r*_EO_ = 1.6, *r*_PO_ = 0.36.^12,18^

These observations underscore the significant influence of both temperature and solvent on the copolymerization behavior of EO and PO, particularly regarding reactivity ratios and the resulting polymer microstructure. Given that such microstructural differences can substantially affect the physical properties of copolymers, it is plausible that variations in polymerization conditions may also translate into measurable changes in macroscopic behavior. A key property in this context is thermoresponsive behavior in aqueous solution. To explore this potential structure–property relationship, we next turn to the thermoresponsive behavior of P­(EO-*co*-PO), with a particular focus on how composition and polymerization conditions influence this property.

### Cloud Point Temperatures by Turbidimetry Measurements

Copolymers of EO and PO are known for their thermoresponsive behavior. They precipitate from aqueous solutions upon heating.^79^ The thermoresponsive nature of the solutions is typically characterized by the cloud point temperature (*T*_cp_). The *T*_cp_ indicates at which temperature a polymer solution transitions from a homogeneous solution to phase separation due to dehydration of the polymer chains.^80^ Below *T*_cp_, P­(EO-*co*-PO) copolymers remain fully soluble in water, whereas exceeding this temperature leads to aggregation and precipitation of the polymers. In one of the earliest investigations, Bailey and Callard reported *T*_cp_ values between 47 and 60 °C for copolymers containing approximately 33 mol % PO monomer, with extrapolation of their results suggesting that aqueous solubility is lost, when the PO content approaches 50 mol %.^14^ However, the absence of detailed information on the molar mass and synthesis conditions restricts broader applicability of these findings. Similar constraints apply to other studies, such as those by Tjerneld et al. and Louai et al., who investigated either commercially available copolymers with unclear synthesis conditions and molar mass or structurally distinct architectures such as three-arm star-shaped polymers.^13,16^ These differences in polymer topology and uncertainty about the synthesis conditions complicate the interpretation of thermoresponsive behavior and impede establishment of general structure–property relationships. Consequently, a systematic investigation across a broad EO/PO composition range, using well-defined linear random copolymers, has been missing in the literature to date.

One major reason for this gap might be the inherent synthetic challenges in the controlled copolymerization of EO and PO, particularly at elevated PO molar fractions. Here the propagation rate slows down dramatically. Under such conditions, the reaction times to full conversion can extend to several weeks, posing a significant obstacle to the efficient synthesis of well-defined copolymers. Furthermore, the increased occurrence of proton abstraction as a side reaction at elevated PO contents complicates precise control and prediction of the molar mass and thereby limits systematic investigation across the full compositional spectrum.^17,81^

To fill this gap, we synthesized a series of well-defined statistical P­(EO-*co*-PO) copolymers in toluene with degrees of polymerization around 45 units, comparable to PEG 2000 (see Table 3). In contrast to the kinetics experiments, triethylene glycol monomethyl ether was used as an initiator instead of 2-(benzyloxy)­ethanol, as its structure is more similar to the polymer backbone and therefore expected to exert less influence on physical properties. The resulting library spans EO contents from 35 to 90 mol %, thereby covering a broad compositional range (Table 3, Entries 2–5). To ensure high monomer conversion across all compositions and to minimize monomer loss, particularly the volatilization of EO and PO during prolonged reaction times, the polymerizations were conducted in sealed reactors under static vacuum using extended reaction times. This approach was critical to reliably obtain copolymers with well-controlled molar masses across the entire EO/PO spectrum. For comparison, a commercially available PPO homopolymer (Table 3, Entry 1) was also included in the study to extend the data set toward the PO-rich end of the spectrum. All polymers exhibit narrow molar mass distributions, as confirmed by SEC analysis (see Figure S92).

**Table 3:** Characterization Data for the Series of Statistical P­(EO-*co*-PO) Copolymers Synthesized in Toluene with Varying mol %_EO_ and the Corresponding Cloud Point Temperature *T*_cp_ in Aqueous Solution

entry	mol %_EO,theo_/%	mol %_EO_ ^a^/%	*t* ^b^ */* h	*T*/°C	*M*_*n*,theo_/g·mol^–1^	*M_n_* ^c^/g·mol^–1^	*M_n_* ^d^/g·mol^–1^	*Đ* ^d^	*T*_cp_ ^e^/°C
1^f^	0	0	^g^	^g^	2700	2800	3200	1.04	11.80 ± 0.08
2	30	35	504	40	2590	2200	2100	1.06	40.10 ± 0.08
3	50	55	432	40	2460	2500	2300	1.06	64.83 ± 0.39
4	70	70	504	40	2340	2400	2200	1.04	91.33 ± 0.39
5	90	90	336	40	2210	2700	2300	1.06	>100

a Determined by ^1^H NMR spectroscopy.

b Experimental reaction time.

c Determined via MALDI-ToF MS measurement (CHCl_3_, DCTB matrix).

d Determined via SEC measurements (DMF, RI signal, PEG calibration).

e Cloud point temperature determined in aqueous solution via turbidimetry for *c* = 5 mg·mL^–1^.

f PPO (2.7 kg·mol^–1^) purchased from Merck KGaA.

g Unknown (purchased compound from Merck KGaA).

The thermoresponsive properties of the synthesized polymers were evaluated by turbidimetry to determine the influence of the variation of the EO-PO ratio on their physical characteristics. Commonly, turbidimetry is employed to determine *T*_cp_. This technique records changes in light transmittance as the temperature increases. When the solution reaches *T*_cp_, concentrated polymer droplets form, scattering light and causing a sudden decrease in transmittance. This effect can be conveniently measured with a standard UV–vis spectrometer equipped with temperature control. *T*_cp_ can be finely tuned by adjusting the hydrophilic–hydrophobic balance of the copolymer chains.^3^ This tuning can be achieved via copolymerization^82^ or end-group alterations,^83^ as well as through physical factors like ionic strength,^16,84^ making *T*_cp_ highly adaptable to meet specific application requirements. *T*_cp_ values are closely related to the polymer microstructure. Therefore, variations in the arrangement of monomer units or end groups may alter intermolecular interactions, thereby impacting the temperature at which phase separation occurs.^8^

The synthesized copolymers, together with a commercially available PPO homopolymer, cover a wide compositional range with EO contents from 0 to 95 mol %, exhibiting *T*_cp_ spanning a broad range from approximately 12 °C to more than 100 °C (see Figure 6 and Table 3). This broad *T*_cp_ range encompasses temperatures that are highly relevant for various aqueous systems and their application.

**Figure 6:**
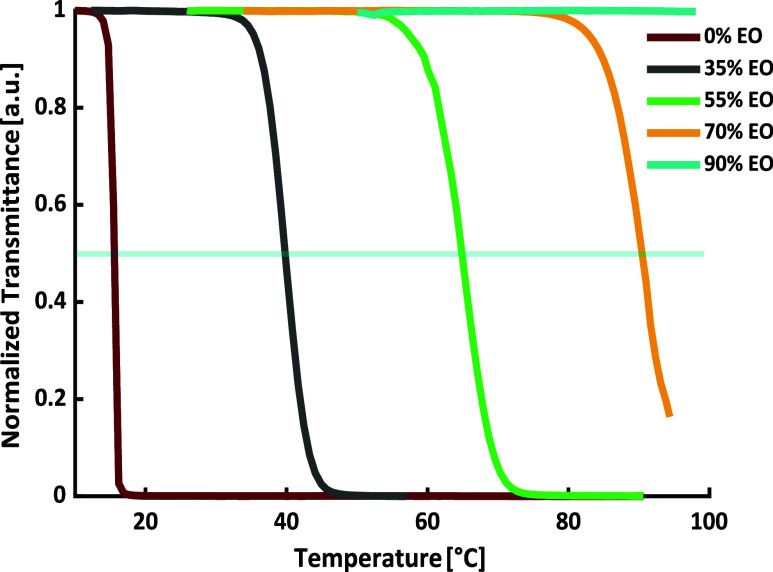
Results of the turbidimetry measurements in aqueous solution (*c* = 5 mg·mL^–1^) of the copolymers of EO and PO obtained in toluene at 40 °C (Table 3), varying EO content. The blue line represents 50% normalized transmittance, which is used to determine the cloud point temperatures.

Notably, the cloud point temperatures show an approximately linear dependence on the EO content (see Figure 7). This relationship offers a straightforward approach to tailoring the thermoresponsive properties of EO-PO copolymers by adjusting their composition. Although small deviations from linearity are present, likely due to slight deviations from the targeted molar mass associated with the synthetic challenges of high PO content copolymers, the overall correlation is strong and provides a reliable basis for prediction (*R* ^2^ = 0.95). All *T*_cp_ measurements were performed in triplicate to ensure reproducibility and reliability of the data. Across all samples, the standard deviations were consistently below ± 1 °C, demonstrating the high precision of the measurements. Due to this minimal variation, error bars are not visible in the graph shown in Figure 7. The empirical fit derived here thus serves as a practical guide for designing EO-PO copolymers with specific cloud point temperatures aligned to targeted applications using eq 3.

3 $$T_{\mathrm{cp}}\left({}^{\circ}C\right)=1.1\cdot \left({\mathrm{mol}\%}_{\mathrm{EO}}\right)+7.8$$

These findings, however, are based on copolymers synthesized under a single set of reaction conditions, namely at 40 °C in toluene, with the reactivity ratios *r*_PO_ = 0.26 and *r*_EO_ = 3.78. When other synthesis conditions are applied, such as different solvents or temperatures, the reactivity ratios change (see Table 2), which in turn presumably slightly modifies the fit parameters in eq 3. In addition, the analysis was carried out for copolymers containing 45 repeating units. Increasing the chain length will likewise affect the fit parameters. Based on the results of our preceding kinetic investigations, these specific conditions are associated with relatively low amounts of allylic species and reasonably high reaction rates, thereby enabling the synthesis of well-defined copolymers with minimal side reactions. Having established that the reaction conditions influence the microstructure of the copolymers, we next aimed to investigate how these structural differences translate into changes in thermoresponsive behavior and bulk physical properties such as crystallinity and melting transitions.

**Figure 7:**
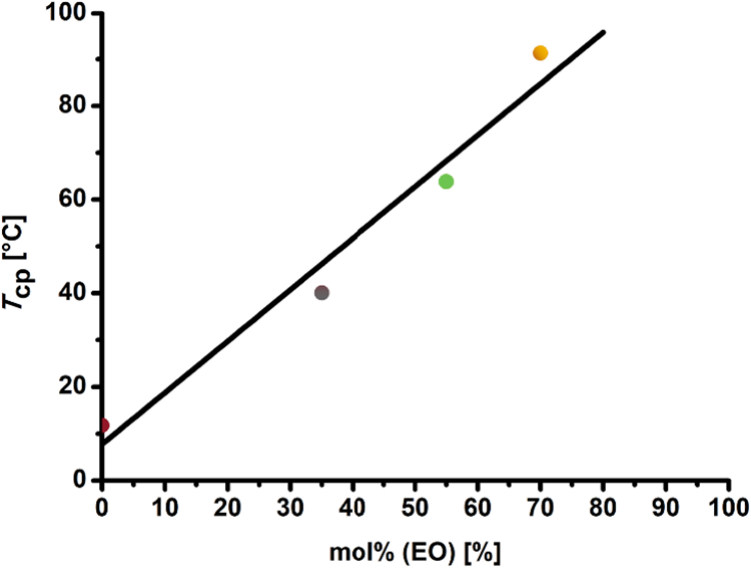
Cloud point temperatures (*T*_cp_) of P­(EO-*co*-PO) copolymers as a function of their ethylene oxide content (mol % EO), based on data from Table 3. The solid line represents a linear regression fit of the experimental data, performed to quantify the correlation between copolymer composition and *T*_cp_. The experimental error (< ± 1 °C) is too small to be visible in the figure.

To examine this question, a second series of P­(EO-*co*-PO) copolymers was synthesized under systematically varied reaction conditions. In addition to evaluating the thermoresponsive behavior in aqueous solution, this set of experiments aimed to investigate how changes in polymerization temperature and solvent affect bulk physical properties such as crystallinity. These characteristics are strongly influenced by the copolymer microstructure, which in turn is shaped by the reactivity ratios of the copolymerization. Thus, correlating reaction conditions with both solution and bulk properties offers a more comprehensive understanding of how subtle variations in synthesis conditions impact the performance of P­(EO-*co*-PO) in practical applications.

### Synthesis of P­(EO-*co*-PO) Copolymers: Impact of Solvent and Reaction Temperature

Despite the widespread use of EO/PO copolymers, we have been unable to find a compilation of the physical properties of the copolyethers, neither in aqueous solution nor in bulk. To explore the potential impact of varying solvents and reaction temperatures on the physical properties, a series of P­(EO-*co*-PO) copolymers was synthesized under meticulously controlled conditions. All copolymers were prepared to have a comparable molar mass (≈5000 g/mol) and EO content of around 70 mol %. Molar mass and comonomer ratio were selected to produce semicrystalline materials with distinct melting endotherms and measurable cloud point temperatures. All other polymerization parameters, except for solvent and reaction temperature, were held constant. The chosen solvents and temperature conditions matched those used in the kinetic studies. However, unlike the kinetics experiments, the initiator applied here was again triethylene glycol monomethyl ether rather than the less polar 2-(benzyloxy)­ethanol. Due to its structural similarity to the polymer backbone, triethylene glycol monomethyl ether was selected to minimize the influence of the initiator on the copolymers’ physical properties. Furthermore, polymerizations at room temperature had to be carried out with the addition of crown ether, as the reaction rates in anisole and toluene were so low at this temperature that achieving molar masses sufficient for the characterization of crystallinity and thermoresponsive properties would not be feasible within a practical time frame.

The initiator salt synthesis was conducted in accordance with the protocol for the kinetic measurements, involving heating of triethylene glycol monomethyl ether with KO^*t*^Bu, followed by overnight solvent removal. However, unlike in the kinetic experiments, both EO and PO monomers were introduced under static vacuum, while the reaction mixture was maintained at −78 °C in an ethanol/liquid nitrogen bath. Additionally, these polymerizations were performed in a reaction flask rather than an NMR tube, with a degree of deprotonation of 90% to reduce the reaction time. Upon completion, the polymerizations were terminated.

The reaction times were 72 h for copolymerizations in toluene above 40 °C and DMSO. From preliminary experiments it was known that copolymerizations in apolar solvents at lower temperatures required almost 12 days to obtain full conversion. This included an extra reaction time of 2–4 days to ensure completion due to safety precautions. The resulting polymers were purified by dialysis against *Milli*-*Q* water, followed by characterization using NMR spectroscopy, SEC, and MALDI-ToF mass spectrometry. The synthesized copolymers are summarized in Table 4. The obtained ^1^H NMR spectra (Figures S86–S91), SEC traces (Figure S93) and mass spectra (Figures S99–S104) are given in the SI.

**Table 4:** Characterization Data for the Series of Statistical P­(EO-*co*-PO) Copolymers and the Corresponding Cloud Point Temperature *T*_cp_ in Aqueous Solution

entry	mol %_EO_ ^a^/%	solvent	*T*/°C	Δ*r* ^b^	*M*_n_ ^c^/g·mol^–1^	*M*_n_ ^d^/g·mol^–1^	*Đ* ^d^	*T*_cp_ ^e^/°C
1	72	toluene^f^	25	2.72	4600	4200	1.11	76
2	75	toluene	40	3.52	4500	4200	1.09	86
3	73	toluene	50	3.34	4900	4300	1.07	80
4	72	toluene	60	2.90	4900	4400	1.06	78
5	72	anisole	40	3.24	4900	4200	1.05	79
6	72	DMSO	40	2.78	4700	4200	1.05	78

a Determined by ^1^H NMR spectroscopy.

b Δ*r* = *r*_EO_–*r*_PO_.

c Determined via MALDI-ToF MS measurement (CHCl_3_, DCTB matrix).

d Determined via SEC measurements (DMF, RI signal, PEG calibration).

e Cloud point temperature determined in aqueous solution via turbidimetry for *c* = 5 mg·mL^–1^.

f 2 equiv [18]­crown-6 per potassium.

The molar mass of the copolymers could not be determined by ^1^H NMR spectroscopy due to the overlap of the initiator’s methyl group signal with signals from the copolyether backbone. SEC measurements underestimated the molar mass compared to values obtained from MALDI-ToF MS analysis. This discrepancy arises because SEC, a relative method calibrated with PEG standards, does not account for the reduced hydrodynamic volume caused by the incorporation of the PO comonomer, leading to lower calculated molar masses. In contrast, MALDI-ToF MS analysis provided molar masses that were consistent with the target values of approximately 5000 g/mol. The comonomer ratio was calculated from the ratio of the copolymer backbone signal and the signal of the methyl group stemming from the PO units (eq S1).

The cloud point temperatures of all synthesized P­(EO-*co*-PO) copolymers were investigated. The temperature-dependent transmittance of the polymer solution in *Milli*-*Q* water with a concentration of 5 mg·mL^–1^ was determined. The *T*_cp_ is defined as the temperature at which the normalized transmittance decreases to 50%. Figures S105 and S106 illustrate the transmittance vs temperature profiles for the temperature and solvent variation series, respectively. The synthesized copolymers are summarized in Table 4 along with their cloud point temperatures *T*_cp_.

### Influence of Polymerization Temperature on *T*_cp_

A comparison of the copolymers synthesized in toluene at different temperatures (72–75 mol %_EO_) shows that decreasing polymerization temperatures from 60 to 40 °C led to an increase in the cloud point temperature from 78 to 86 °C (see Table 4, Entries 1–4, and Figure 8).

**Figure 8:**
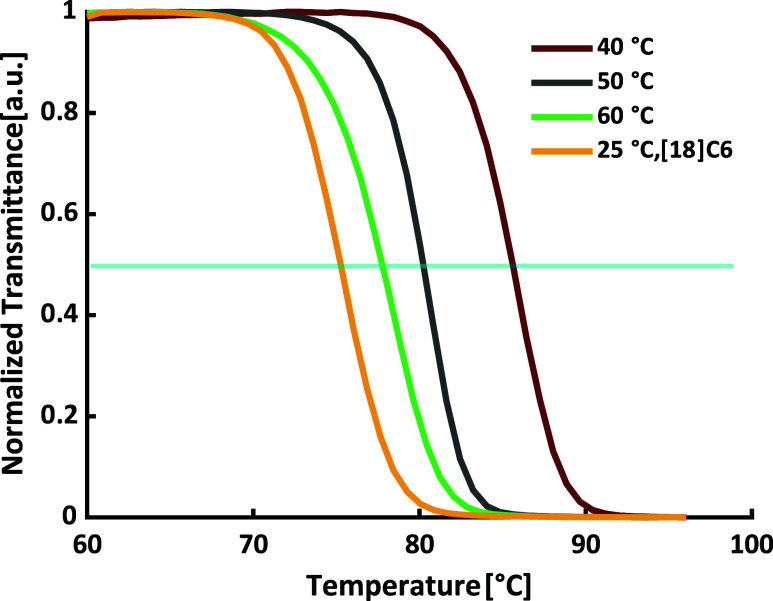
Results of the turbidimetry measurements in aqueous solution (*c* = 5 mg·mL^–1^) of the polymers obtained from toluene at 25 °C with [18]­crown-6, 40, 50, and 60 °C. The blue line represents 50% normalized transmittance, which is used to determine the cloud point temperatures.

Obviously, the polymerization temperature has a significant influence on the thermoresponsive behavior of P­(EO-*co*-PO) copolymers of the same composition. This is due to the microstructure of the copolymers. As determined via online kinetics, the polymerization temperature affects the reactivity ratios of the copolymerization, altering the monomer gradient.

The nature of this compositional gradient is reflected by the difference in the reactivity ratios as a proxy (Δ*r* = *r*_EO_ – *r*_PO_). The observed increase in *T*_cp_ in our data indicates enhanced solubility in water, demonstrating that the copolymers with a steeper gradient synthesized in this study dissolve more readily than those with a gradual composition gradient (Figure 9).

**Figure 9:**
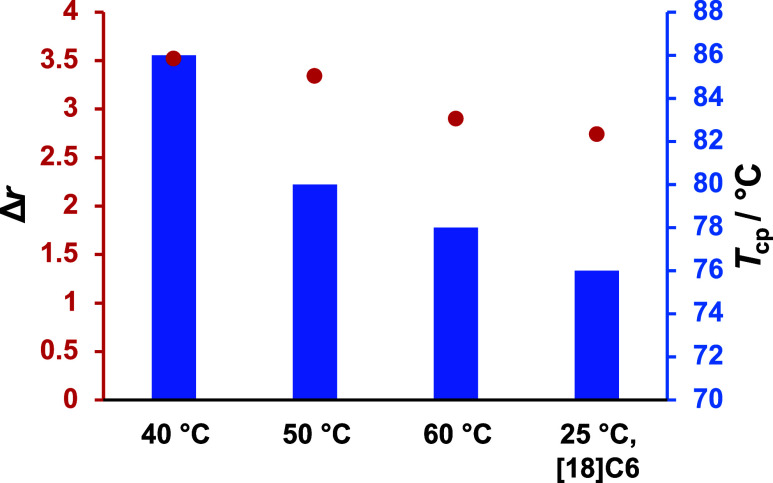
Cloud point temperature (*T*_cp_, blue bars) and reactivity ratio difference (Δ*r*, red line) of P­(EO-*co*-PO) copolymers synthesized at various polymerization temperatures in toluene. The right column represents a polymerization conducted at room temperature in toluene with the addition of crown ether, while all other polymerizations were performed in pure toluene without crown ether.

This is explained by the clustering of EO units in EO-dominated chain segments formed early in the copolymerization, which are highly hydrophilic. Consequently, polymers synthesized at lower temperatures likely possess a sharper gradient, resulting in more distinct EO-rich segments and thereby higher aqueous solubility compared to those synthesized at elevated temperatures. The results of the online kinetics measurements correlate with this observation. The reactivity ratios for polymerizations conducted at 40 °C in toluene exhibit a greater disparity (*r*_PO_ = 0.26, *r*_EO_ = 3.78, Δ*r* = 3.52, Entry 2 in Table 4) than those for polymerizations at 60 °C (*r*_PO_ = 0.31, *r*_EO_ = 3.21, Δ*r* = 2.90, Entry 4 in Table 4), which aligns with a steeper compositional gradient in the first case. The reactivity ratio difference for the copolymer synthesized at 25 °C with crown ether (*r*_PO_ = 0.33, *r*_EO_ = 3.05, Δ*r* = 2.72, Table 4, Entry 1) is the smallest compared to those synthesized in toluene at 40–60 °C, which also results in reduced aqueous solubility.

The reactivity ratios do not exhibit as pronounced a difference as typically observed in carbanionic polymerizations in apolar solvents.^85^ However, the variations in gradient, as anticipated, significantly influence the physical properties, particularly the aqueous solubility of the copolymers.

### Influence of Solvent on *T*_cp_

An examination of the cloud point temperatures for polymers synthesized in different solvents at 40 °C strongly suggests that solvent choice plays a significant role in influencing copolymer properties (Figure S106). The *T*_cp_ values increase by 8 °C from the copolymer synthesized in the polar solvent DMSO (78 °C, Table 4, Entry 6) to the one synthesized in the apolar solvent toluene (86 °C, Table 4, Entry 2), indicating that solvent polarity also influences the solubility behavior of the copolymers (Figure 10). This trend is further supported by the reactivity ratios: in DMSO (*r*_PO_ = 0.32, *r*_EO_ = 3.10, Δ*r* = 2.78), the reactivity ratios exhibit a smaller difference than those observed in toluene (*r*_PO_ = 0.26, *r*_EO_ = 3.78, Δ*r* = 3.52), which corresponds to a softer compositional gradient in DMSO and a more segmented structure when prepared in toluene. The *T*_cp_ of the copolymer synthesized in anisole (79 °C, Table 4, Entry 5) is similar to that of the copolymer synthesized in DMSO, despite the moderate difference in the reactivity ratios. This suggests the existence of a threshold below which differences in reactivity ratios have a minor effect. Another possible explanation could be residual solvent impurity in the copolymers synthesized with anisole, leading to a lower *T*_cp_, but all materials were dialyzed and analyzed, which is why we strongly decline this hypothesis. However, further investigation is subject of a follow-up study.

**Figure 10:**
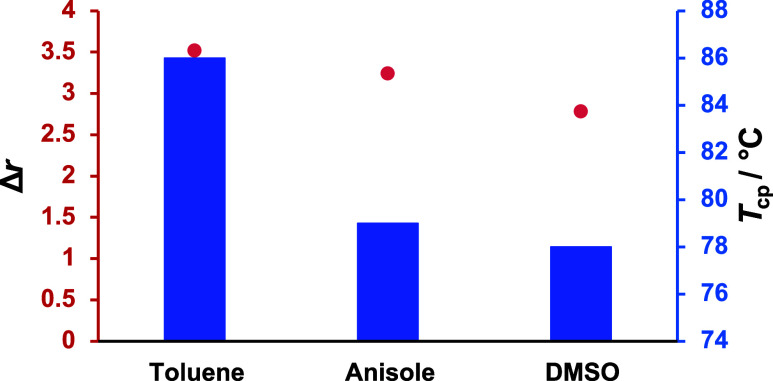
Cloud point temperature (*T*_cp_, blue bars) and reactivity ratio difference (Δ*r*, red line) of P­(EO-*co*-PO) copolymers synthesized at 40 °C in different solvents (≈72 mol % EO, toluene, anisole, DMSO).

### Melting Temperature and Enthalpy by DSC Measurements

Thermal properties were examined by differential scanning calorimetry (DSC) measurements. The corresponding DSC curves are shown in the SI (Figures S107–S108). Longer sequences of EO units along the copolymer backbone are expected to enhance not only the solubility of the copolymers in water but also their crystallinity. This increased crystallinity should, in turn, result in higher melting temperatures and higher melting enthalpies.

The copolymers exhibited similar thermal properties, with glass transition temperatures (*T*_g_) around −70 °C, regardless of the copolymerization conditions. The melting endotherms were broad, with maxima near 10 °C, approximately 55 K lower than that of a corresponding PEG homopolymer.^28^ This behavior is typical of many gradient copolymers, as the crystalline PEG sequences in the P­(EO-*co*-PO) copolymers are disrupted by PO comonomer units in the copolymer chains.^86–88^ The different copolymerization conditions listed in Table 5 are associated with a compositional gradient, as reflected by the difference in reactivity ratios (Δ*r* = *r*_EO_ - *r*_PO_). However, the melting points (*T*_m_) and melting enthalpies (Δ*H*) remain nearly identical, indicating that the observed variations in reactivity ratios have a minor influence on the physical bulk properties of the copolymers. It is important to emphasize that the differences in reactivity ratios are small, and these results align with expectation. Notably, all copolymers exhibited a PEG melting point despite the incorporation of 30 mol % PO comonomer. This is attributed to the gradient microstructure. In pronounced contrast, related glycidyl ethers exhibit random or near-random incorporation when copolymerized with EO in AROP (*r*_EO_ ≈ *r*_GE_ ≈ 1),^29,42,89–92^ leading to a random distribution of comonomer units along the polyether backbone. As a result, the typically crystalline PEG domains are prevalently disrupted, reducing crystallinity as the comonomer content increases. EO copolymers with glycidyl ether comonomer contents of approximately 15–20 mol % become fully amorphous.^43,89^ This highlights the influence of reactivity ratios on the thermal properties of copolymers, although the differences observed in this study were too small to produce pronounced effects.

**Table 5:** Characterization Data for the Series of Statistical P­(EO-*co*-PO) Copolymers and the Corresponding Thermal Properties

entry	mol %_EO_ ^a^/%	solvent	*T*/°C	Δ*r* ^b^	*M*_n_ ^c^/g·mol^–1^	*M*_n_ ^d^/g·mol^–1^	*Đ* ^d^	*T*_g_ ^e^/°C	*T*_m_ ^f^/°C	Δ*H* ^g^/J·g^–1^
1	72	toluene^h^	25	2.72	4600	4200	1.11	–69	5	38
2	75	toluene	40	3.52	4500	4200	1.09	–69	13	42
3	73	toluene	50	3.34	4900	4300	1.07	–69	10	40
4	72	toluene	60	2.90	4900	4400	1.06	–70	7	39
5	72	anisole	40	3.24	4900	4200	1.05	–70	8	39
6	72	DMSO	40	2.78	4700	4200	1.05	–70	6	38

a Determined by ^1^H NMR spectroscopy.

b Δ*r* = *r*_EO_–*r*_PO_.

c Determined via MALDI-ToF MS measurement (CHCl_3_, DCTB matrix).

d Determined via SEC measurements (DMF, RI signal, PEG calibration).

e Glass transition temperature.

f Melting temperature.

g Melting enthalpy.

h 2 equiv [18]­crown-6 per potassium.

## Conclusion

Statistical copolymers of EO and PO are of crucial importance for many industrial applications, ranging from polyols for polyurethanes to foam stabilizers and dispersants. Although they are often prepared under solvent-free conditions in industry, the influence of solvents and temperature variation is relevant for several specialty applications. This study aimed at elucidating the effects of solvent choice, temperature, and crown ether addition on the copolymerization of ethylene oxide (EO) and propylene oxide (PO), as well as the resulting physical properties of P­(EO-*co*-PO) copolymers with a comonomer ratio of 70/30 mol % (EO/PO) and a molar mass of ≈5000 g/mol. The potential changes in the physical properties due to different copolymerization conditions were expected to be clearly visible. We investigated the copolymerization by online ^1^H NMR kinetics measurements in DMSO, anisole, and toluene at temperatures ranging from 25 to 60 °C, as well as in the presence of [18]­crown-6 at 25 °C. The highest reaction rates were observed in DMSO, while anisole and toluene exhibited lower, similar rates under most polymerization conditions. Notably, at 60 °C, the reaction rate in anisole was 2.8 times higher than in toluene. For copolymerizations conducted in anisole and toluene, an induction period was observed, attributed to the Weibull-Törnquist or “crown ether” effect, which was eliminated upon adding [18]­crown-6. NMR spectroscopy indicated that the transfer reaction of active chains to PO monomer was significantly higher in DMSO than in toluene and anisole. This lower abundance of copolymer chains initiated by allylic species in the latter solvents is advantageous, however, it is accompanied by the drawback of a reduced reaction rate, especially at low temperatures. This leads to reaction times of up to 12 days in toluene and anisole.

Reactivity ratios in bulk copolymerization of EO and PO were reported as *r*_EO_ = 2.8 and *r*_PO_ = 0.25.^17^ Our online kinetics measurements in DMSO, toluene and anisole revealed higher reactivity ratios for EO in the range of *r*_EO_ = 3.05–3.78 and *r*_PO_ = 0.33–0.26, depending on the copolymerization conditions. In DMSO, we observed copolymerization to produce less pronounced gradient structures compared to anisole and toluene at 25 and 40 °C. With temperature increase the reactivity ratio differences decrease in all solvents. In toluene, reactivity ratio differences initially increase from 25 to 40 °C before converging at higher temperatures. The addition of [18]­crown-6 slightly reduces the difference in reactivity ratios for all solvents at 25 °C. Thermal analysis of the copolymers revealed a broad melting endotherm with a maximum at around 10 °C. Differences in the reactivity ratios had no significant effect on the thermal properties. The P­(EO-*co*-PO) copolymers exhibited a lower melting point compared to a corresponding PEG homopolymer (65 °C).^28^ The gradient comonomer structure leads to EO-rich segments capable of crystallization. Copolymers were synthesized in toluene at various temperatures (40, 50, 60, and 25 °C with [18]­crown-6), and aqueous solubility was assessed via cloud point measurements. A decreased difference in reactivity ratios correlated with a slightly lower cloud point, which can be explained by fewer EO-rich segments in the softer gradient structure responsible for solubility. A similar trend in cloud point reduction was observed in copolymers prepared at 40 °C in all solvents studied.

The results demonstrate that selected reaction conditions significantly impact the physical properties of the polyether copolymers, primarily due to variations in the gradient, as expressed by the reactivity ratios. While the influence of reaction conditions on reactivity ratios may not be predominant, they represent an effective tool for tuning material properties. The parameters studied here – solvent, temperature, and, in part, crown ether addition – constitute some of the possible variables. Future research might further explore the effects of various solvents and solvent mixtures, as well as different degrees of deprotonation. The results underscore the critical role of reaction conditions in shaping the physical properties of P­(EO-*co*-PO) copolymers.

## Supplementary Material



## Notes

Additional experimental procedures, ^1^H NMR, evaluation data for ^1^H NMR copolymerization kinetics, and additional characterization data (SEC, MALDI-ToF, DSC, Turbidimetry) (PDF)
