# Supplementary material for: Reaction Temperature and Solvent Influence Reactivity Ratios in the Copolymerization of Ethylene Oxide and Propylene Oxide
Source: Macromolecules. 2025 Dec 12;58(24):13300–13. doi: 10.1021/acs.macromol.5c02759 (PMC12752706; doi:10.1021/acs.macromol.5c02759)
Supplement: Supplementary file 1 [file ma5c02759_si_001.pdf]

Supporting Information

Reaction Temperature and Solvent Influence Reactivity Ratios in the Copolymerization of Ethylene Oxide and Propylene Oxide

Milena S. Hesse[1], Gregor M. Linden[1], Holger Frey[1,*]

[1]Department of Chemistry, Johannes Gutenberg University Mainz, Duesbergweg 10-14, 55128 Mainz, Germany.

*hfrey@uni-mainz.de

Table of Contents

Experimental Section 2

Reagents 2

Instrumentation 2

Polymerization Procedures 4

Data of copolymerization kinetics 7

Size exclusion chromatography 41

^{1}H NMR Spectroscopy 45

Size exclusion chromatography 51

MALDI-ToF MS Spectrometry 53

Turbidimetry measurements 58

DSC measurements 59

References 61

Experimental Section

Reagents

Ethylene oxide (EO) was procured from *Air Liquide*. Deuterated solvents were purchased from *Deutero GmbH* (Germany). Potassium *tert*-Butanolate (KO^tBu), propylene oxide (PO), 2-(Benzyloxy)ethanol (>98%, GC), and triethylene glycol monomethyl ether were purchased from *TCI GmbH (Germany)* while DMSO and toluene (99% purity over molecular sieve) as well as anisole and [18]crown-6 ([18]C6) were purchased from *Acros/Fisher scientific*. The [18]C6 was taken from a stock solution in dry benzene.

Instrumentation

Nuclear Magnetic Resonance (NMR) Spectroscopy

All ^1H-NMR spectroscopic measurements were performed with a *Bruker Avance II 400* at a frequency of 400 MHz while the kinetic measurements were performed with a *Bruker Avance III HD 400* at a frequency of 400 MHz. The chemical shifts are all reported as ppm in relation to the proton signals of the deuteride solvents. Analysis of all spectra was performed with *MestReNova 14.3.3* from *Mestrelab Research*. When kinetics in anisole (non-deuterated) were measured, the lock was turned off. Shim was performed with *TopShim* (gradient shim) using the protons of the anisole methoxy group.

Size Exclusion Chromatography (SEC)

SEC characterization was performed using an *Agilent 1100 series* system, equipped with an UV-detector (254 nm) and RI-detector. *N*,*N*-dimethylformamide (DMF) was utilized as eluent, with 1 g/L LiBr. The applied HEMA 300/100/40 Å column cascade was heated by a column oven, set to 50 °C. The measurements were performed with a flow rate of 1 ml/min. One drop of internal standard (toluene) was added to the samples. Poly(ethylene oxide) standards from *Polymer Standard Service* (*PSS*) were used for calibration. Data were recorded and processed using the software *PSS WinGPC Unichrom*.

Matrix-assisted Laser Desorption Ionization Time-of-Flight (MALDI-ToF) Mass Spectrometry

MALDI-ToF MS measurements were carried out at a *Bruker autoflex maX MALDI-TOF/TOF* using a smartbeam-II solid state laser with a wavelength of 337 nm. The potassium salt of triflouroacetic acid (KTFA, ≥99%, HPLC-grade, *Sigma Aldrich*) and *trans*-2-[3-(4-*tert*-Butylphenyl)-2-methyl-2-propenylidene]malononitrile (DCTB, >98%, *TCI GmbH (Germany)*) were utilized as ionization salt and matrix, respectively. For sample preparation the polymers were dissolved in chloroform at 10 mg/mL. 20 µL of this solution were combined with 20 µL of a 10 mg/mL solution of the matrix in chloroform. 5 µL of a 0.1 M solution of the salt in methanol were added and 1 µL of the resulting mixture was

spotted onto a *MTP 384* ground steel target plate. The solvents were allowed to evaporate completely before the measurement.

Differential Scanning Calorimetry (DSC)

The differential scanning calorimetry measurements were carried out with a *DSC250* device from *TA Instruments*. For this purpose, 10–15 mg of the polymers were weighed in *Tzero* aluminum pans. Before the actual measurement, the sample was first heated to 90 °C and recrystallized at a cooling rate of 3 °C/min to obtain an ordered structure of the polymer. The measurements were then carried out over a temperature range of -90 °C to 70 °C at a heating rate of 10 °C/min. The data obtained was analyzed using the second heating curve by *Trios* software from *TA Instruments*.

Turbidimetry

A *Jasco V-730* spectrometer was used for turbidimetry measurements. The polymers were dissolved in *Milli-Q*® water (5 mg/mL) and 0.7 mL of this solution were transferred to a glass cuvette. The transmission of this solution was then determined over a temperature range of 60 °C to 100 °C using a heating rate of 1 °C/min at a wavelength of λ = 600 nm. The data was obtained using *JASCO spectra manager* version 2.

Polymerization Procedures

Synthesis of statistical P(EO-*co*-PO) copolymers

The following section provides a representative synthesis procedure for the Poly(EO-*co*-PO) copolymers. For the synthesis of statistical P(EO-*co*-PO) copolymers, 1 eq. of triethylene glycol monomethyl ether (0.14 mg, 0.14 mL, 0.9 mmol) was dissolved in a mixture of 2 mL of benzene and 1 mL of THF. This solution was added to an anionic flask in Ar counterflow. Afterwards, 0.9 eq. of KO^tBu (0.89 mg, 0.8 mmol) dissolved in 2 mL of THF and 2 drops of *Milli-Q*® water were added as well. This mixture was frozen using a liquid nitrogen bath and static vacuum conditions were established. Thereafter, the mixture was heated to 60 °C and stirred for 1 hour. The potassium salt of the initiator triethylene glycol monomethyl ether was formed under removal of the solvents and *tert*-butanol in vacuo overnight. In the crown ether containing polymerizations, 1.8 eq. of [18]crown-6 (420 mg, 1.6 mmol) dissolved in benzene were added together with KO^tBu before heating and drying azeotropically.

The initiator salt was dissolved in the respective amount of solvent (DMSO, anisole, toluene) to obtain an initiator concentration of 0.18 M. PO (1753 mg, 2.10 mL, 30.2 mmol), dried over CaH_2 and freshly distilled, was added to the flask under static vacuum conditions and cooling with liquid nitrogen bath. EO (3102 mg, 3.20 mL, 70.4 mmol) was condensed under static vacuum conditions as well by employing an ethanol/liquid nitrogen bath at -78 °C. The polymerization mixture was slowly heated to the respective polymerization temperature (25 °C, 40 °C, 50 °C, 60 °C).

After full monomer conversion, as determined by ^1H NMR spectroscopy, the polymerization was terminated by adding 4 eq. of acetic acid per initiator dissolved in 10 mL diethyl ether and 2 mL toluene. Precipitated salt was filtered using *Celite*® and a por 4 frit and the crude polymer was isolated by removal of the solvents under vacuum at 40 °C. In a final purification step, remaining impurities were removed by dialysis against *Milli-Q*® water using a dialysis membrane (*Spectrum Labs*) with a molecular weight cut-off of 1 kDa. The purified polymer was dried by lyophilization.

The proportions of the respective monomers in the copolymer were determined using ^1H NMR spectroscopy. The ratio of the integrals of the copolymer backbone (3.65–3.30 ppm) and the methyl group of propylene oxide (1.12–1.02 ppm) were used for this purpose following the method of Booth *et al.*[1] The integral of the methyl group was set to 3 protons.

$$\chi_{\mathrm{EO}} = \frac{I_{\mathrm{Backbone}} - 3}{I_{\mathrm{Backbone}} + 1} \tag{S1}$$

It should be mentioned here that the signals of the initiator shift into the backbone signal of the polymer. However, since the initiator is composed of EO units, its contribution was included in the EO content of the polymer.

Investigation of copolymerization kinetics by *in situ* ^{1}H NMR analysis of PO and EO

For copolymerization of PO and EO during an online kinetics experiment, a *Norell S-500-VT-7* sealable NMR tube with a Teflon stopcock was employed. The initiator salt potassium 2-(benzyloxy)ethanolate was prepared in a fivefold batch in a Schlenk flask equipped with a magnetic stirrer in the following manner.

1 eq. of 2-(benzyloxy)ethanol (15 mg, 14 mL, 0.09 mmol) was dissolved in a mixture of 2 mL benzene and 1 mL THF. This solution was added to a Schlenk flask in Ar counterflow. Afterwards, 0.45 eq. of KO^{t}Bu (5 mg, 0.04 mmol) dissolved in 2 mL of THF and 2 drops of *Milli-Q*® water were added as well. This mixture was frozen using a liquid nitrogen bath and static vacuum conditions were established. Thereafter, the mixture was heated to 60 °C and stirred for 1 hour. The partially deprotonated initiator salt potassium 2-(benzyloxy)ethanolate was formed under the removal of the solvents in vacuo overnight.

In the crown ether containing kinetics, 2 eq. of [18]crown-6 were added together with KO^{t}Bu before heating and drying azeotropically.

The initiator salt was dissolved in the respective amount of solvent (2 mL) (DMSO-d_6, anisole, toluene-d_8). Anisole was not deuterated. EO (40 eq., 173 mg, 0.17 ml, 3.9 mmol) was condensed under static vacuum conditions into the NMR tube by employing an acetone/liquid nitrogen bath at -78 °C. PO (4 eq., 23 mg, 0.03 mL, 0.39 mmol), dried over CaH_2 and freshly distilled, and one-fifth of the initiator stock solution with a concentration of 0.16 mol/L was added under Ar counterflow while still cooling. The mixture was subjected to three freeze-pump-thaw cycles to remove residual Ar before being inserted into the preheated NMR spectrometer at the respective temperature. No sample spinning was applied, in the case of deuterated solvents one spectrum was recorded to determine the receiver gain. In the case of anisole, one spectrum was recorded to shim, and a second spectrum was recorded to determine the receiver gain. Online kinetics were conducted with measuring times between 10 and 92 hours with one scan every one to three minutes. After the kinetics, the copolymers underwent SEC

analysis. It is important to note that the kinetic studies conducted using toluene-d_8 as the solvent at 25 °C were monitored offline. In this approach, the polymerization was fully carried out in the kinetic tube under analogous conditions. However, instead of continuous online monitoring throughout the entire polymerization process, the polymerization tube was periodically transferred to the NMR spectrometer for analysis every 24 to 48 hours. This offline monitoring method was chosen because the extended duration of the polymerization made continuous *in situ* tracking impractical.

The respective chemical shift of the PO and EO Epoxide signal was tracked to calculate the individual monomer consumption. PO has three epoxide signals that can be utilized for this purpose. Whenever possible, we used the signal at around 2.38 ppm of the methylene group. When this signal merged with another signal, for example from EO, we utilized the methine proton. The software NIREVAL, created by Frey *et al.*,[2] was utilized to analyze the normalized monomer consumption. The equation for the Jaacks fit can be found in the main manuscript. The equation for the Meyer-Lowry equation with the restriction $r_1 \neq 1$, $r_2 \neq 1$ is as follows:[3]

$$\frac{M}{M_0} = \left(\frac{f_1}{f_{1,0}}\right)^{\frac{r_2}{1-r_2}} \cdot \left(\frac{1-f_1}{1-f_{1,0}}\right)^{\frac{r_1}{1-r_1}} \cdot \left(\frac{f_{1,0} - \frac{1-r_2}{2-r_1-r_2}}{f_1 - \frac{1-r_2}{2-r_1-r_2}}\right)^{\frac{1-r_1 r_2}{(1-r_1)\cdot(1-r_2)}} \tag{S2}$$

With f_1 as the fraction of monomer 1 from the whole unreacted monomers:

$$f_1 = \frac{[\mathrm{M}_1]}{[\mathrm{M}_1] + [\mathrm{M}_2]} \tag{S3}$$

Caveat: EO is a highly flammable and toxic gas, we recommend handling it only by trained researchers. We experienced breakage of the sealable NMR tubes from time to time when it was subjected to liquid nitrogen. We strongly recommend using an acetone/liquid nitrogen cooling bath of -78 °C.

Data of copolymerization kinetics

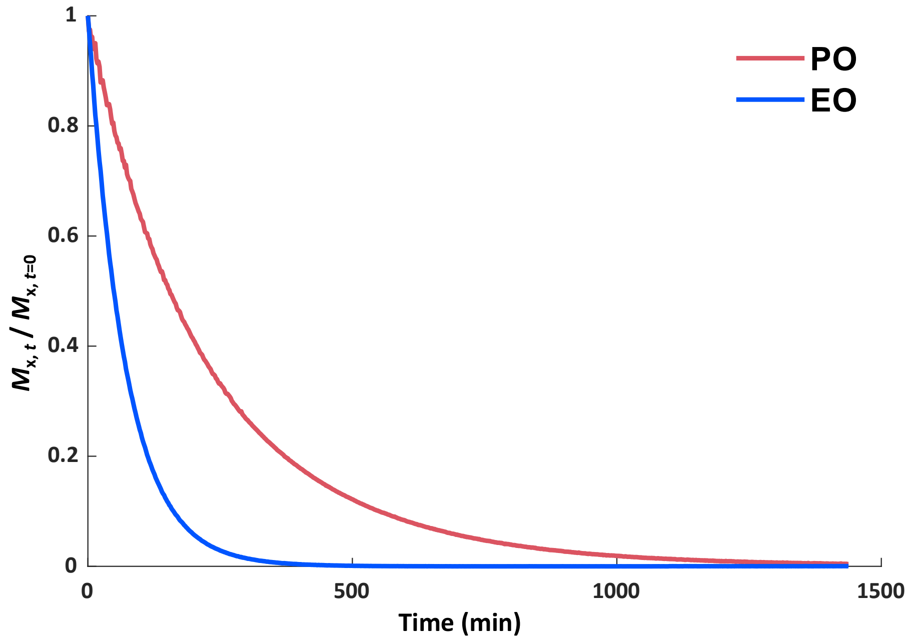

Figure S1: Time-conversion plot of the *in situ* ^{1}H NMR copolymerization kinetic study of EO with PO. (Solvent: DMSO-d_6, 25 °C).

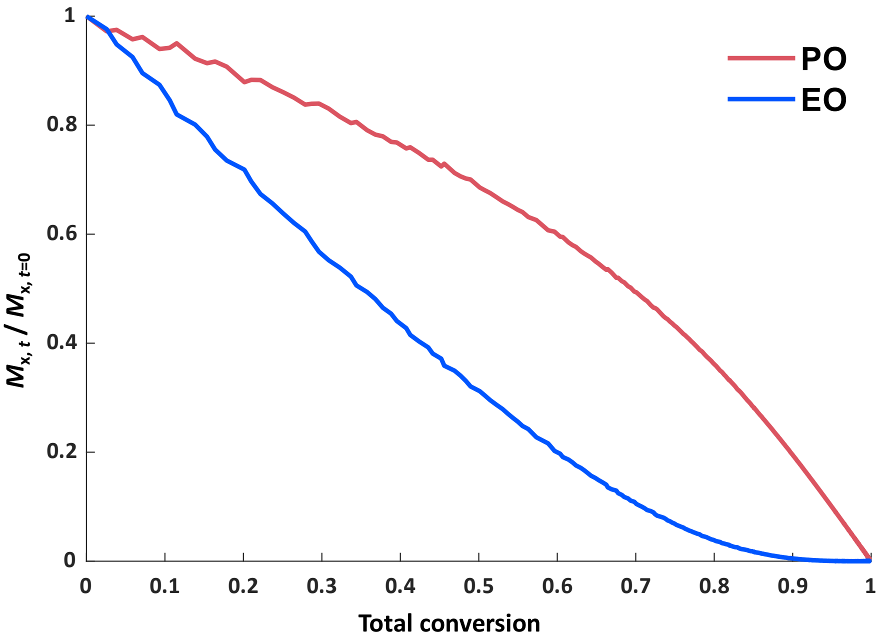

Figure S2: Individual versus total conversion of the *in situ* ^{1}H NMR copolymerization kinetic study of EO with PO. (Solvent: DMSO-d_6, 25 °C).

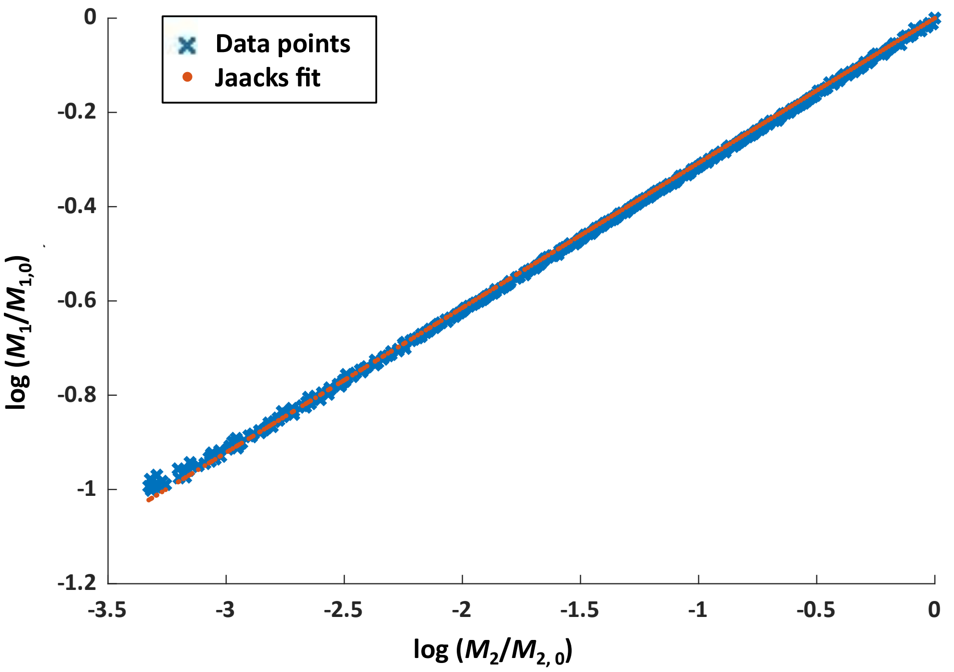

Figure S3: Jaacks fit of the *in situ* ^{1}H NMR copolymerization kinetic study of EO with PO. (Solvent: DMSO-d_6, 25 °C).

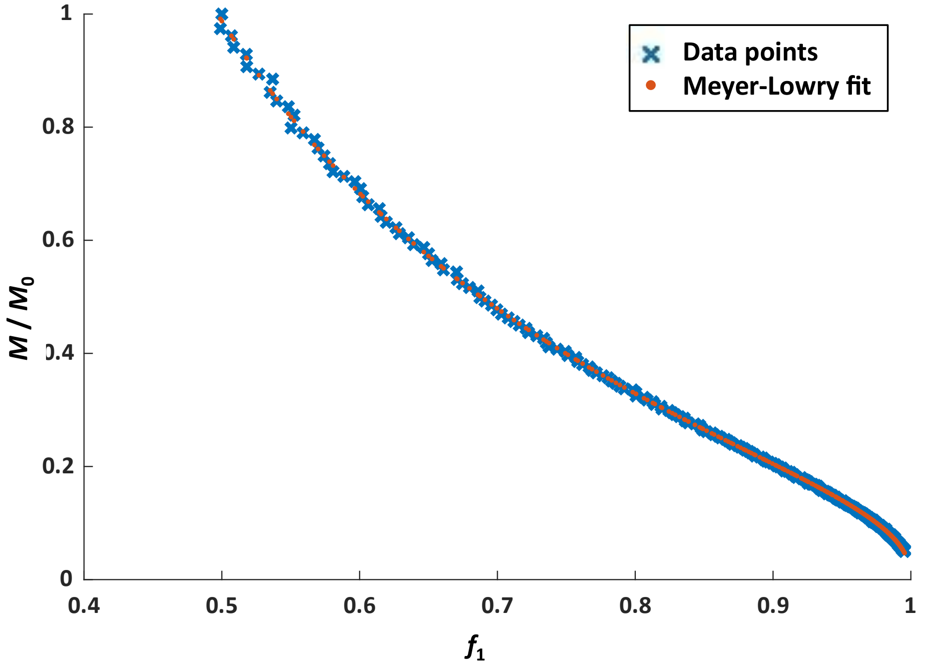

Figure S4: Meyer-Lowry fit of the *in situ* ^{1}H NMR copolymerization kinetic study of EO with PO. (Solvent: DMSO-d_6, 25 °C).

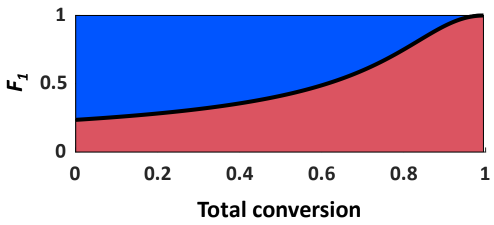

Figure S5: Composition plot of the *in situ* ^{1}H NMR copolymerization kinetic study of EO (blue) with PO (red) with a hypothetical equimolar monomer ratio (Solvent: DMSO-d_6, 25 °C) with *r*(PO)=0.31, *r*(EO)=3.25.

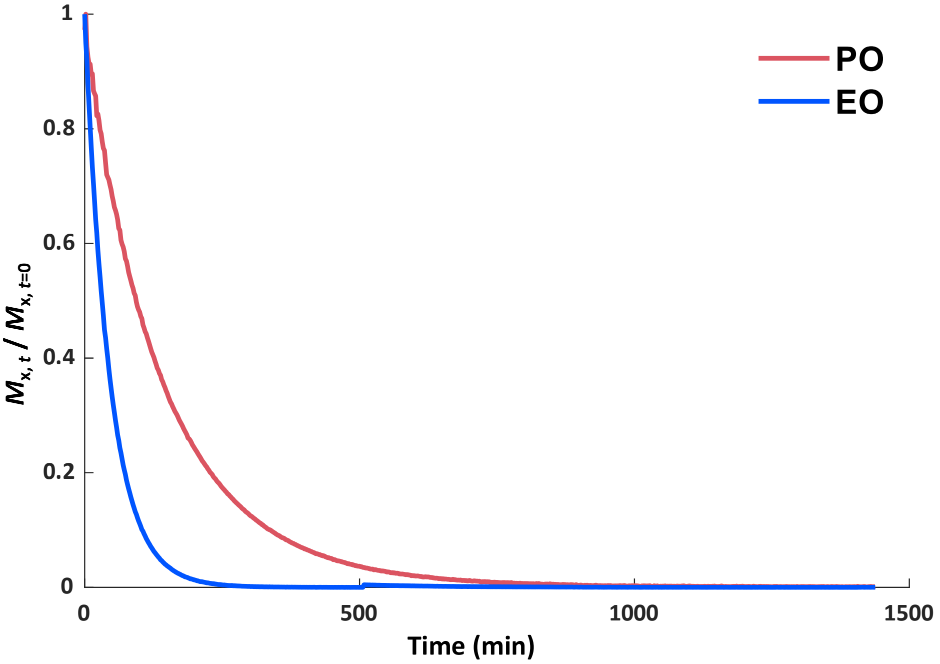

Figure S6: Time-conversion plot of the *in situ* ^{1}H NMR copolymerization kinetic study of EO with PO. (Solvent: DMSO-d_6, 25 °C, addition of [18]crown-6).

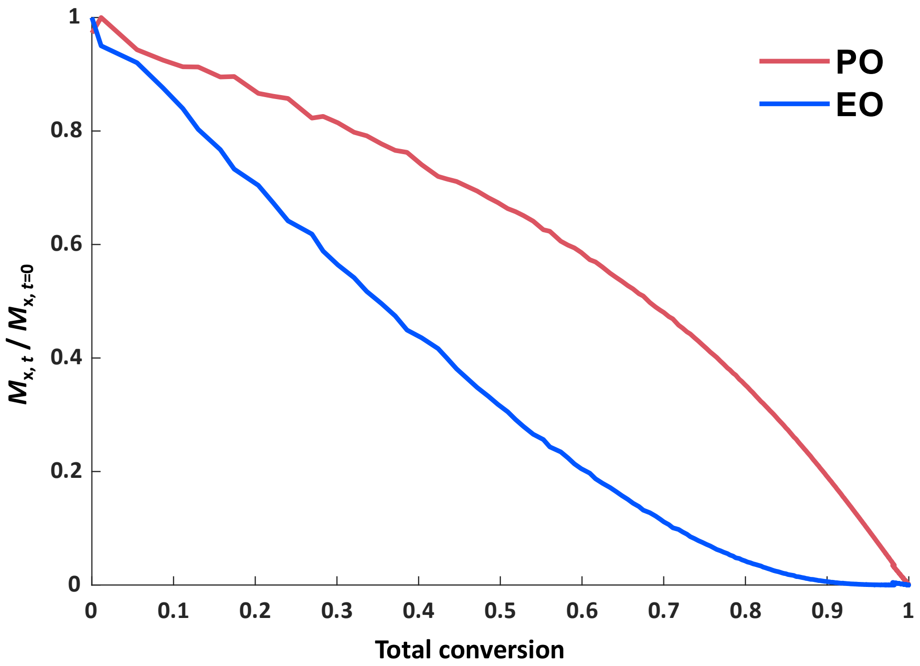

Figure S7: Individual versus total conversion of the *in situ* ^{1}H NMR copolymerization kinetic study of EO with PO. (Solvent: DMSO-d_6, 25 °C, addition of [18]crown-6).

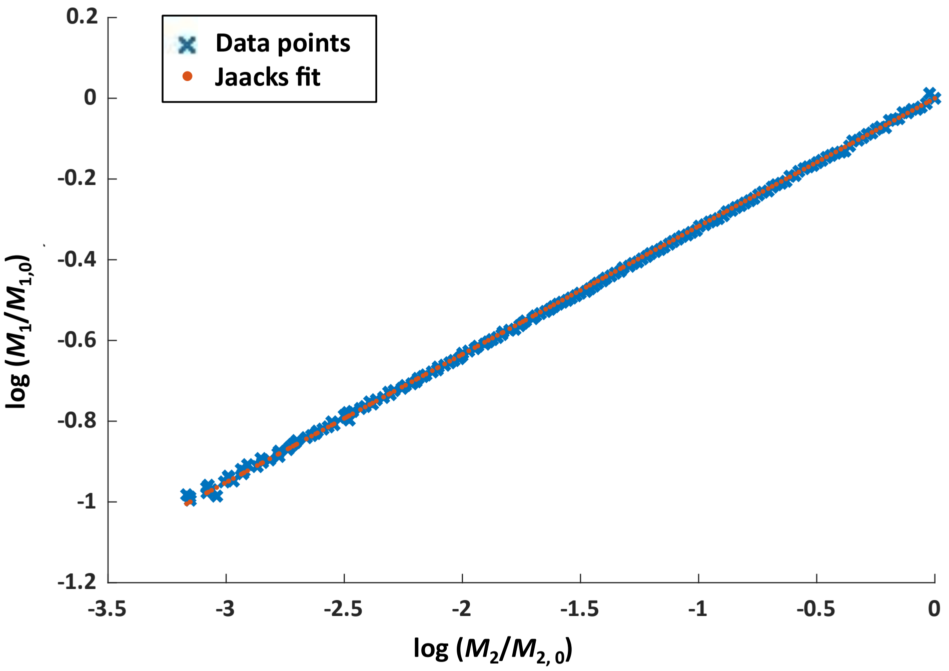

Figure S8: Jaacks fit of the *in situ* ^{1}H NMR copolymerization kinetic study of EO with PO. (Solvent: DMSO-d_6, 25 °C, addition of [18]crown-6).

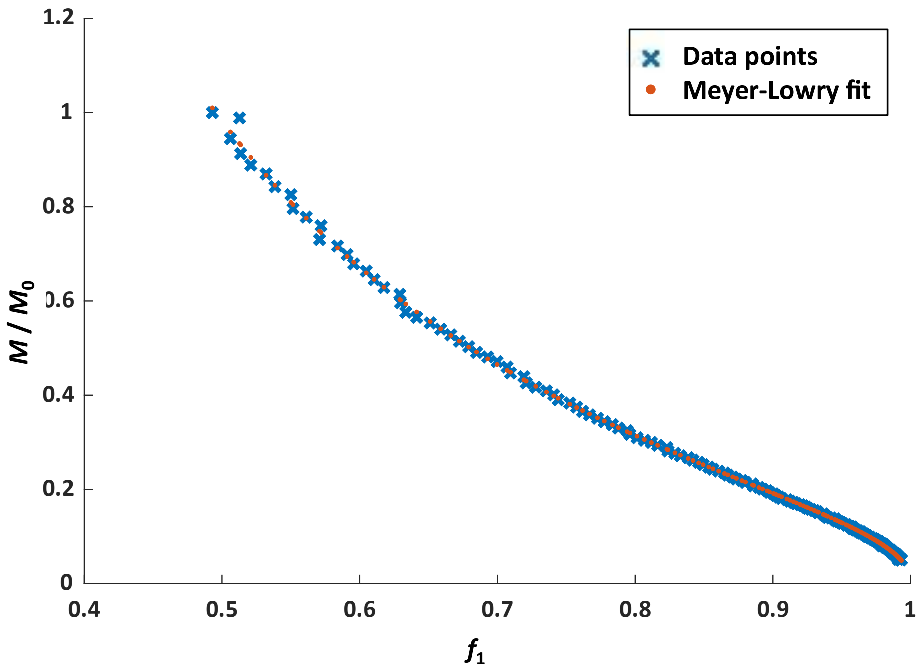

Figure S9: Meyer-Lowry fit of the *in situ* ^{1}H NMR copolymerization kinetic study of EO with PO. (Solvent: DMSO-d_6, 25 °C, addition of [18]crown-6).

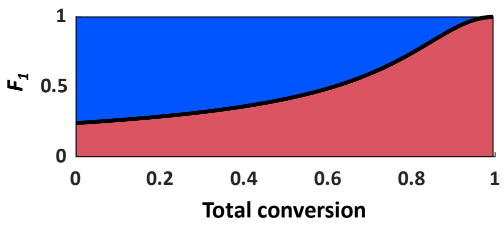

Figure S10: Composition plot of the *in situ* ^{1}H NMR copolymerization kinetic study of EO (blue) with PO (red) with a hypothetical equimolar monomer ratio (Solvent: DMSO-d_6, 25 °C, addition of [18]crown-6) with *r*(PO)=0.32, *r*(EO)=3.15.

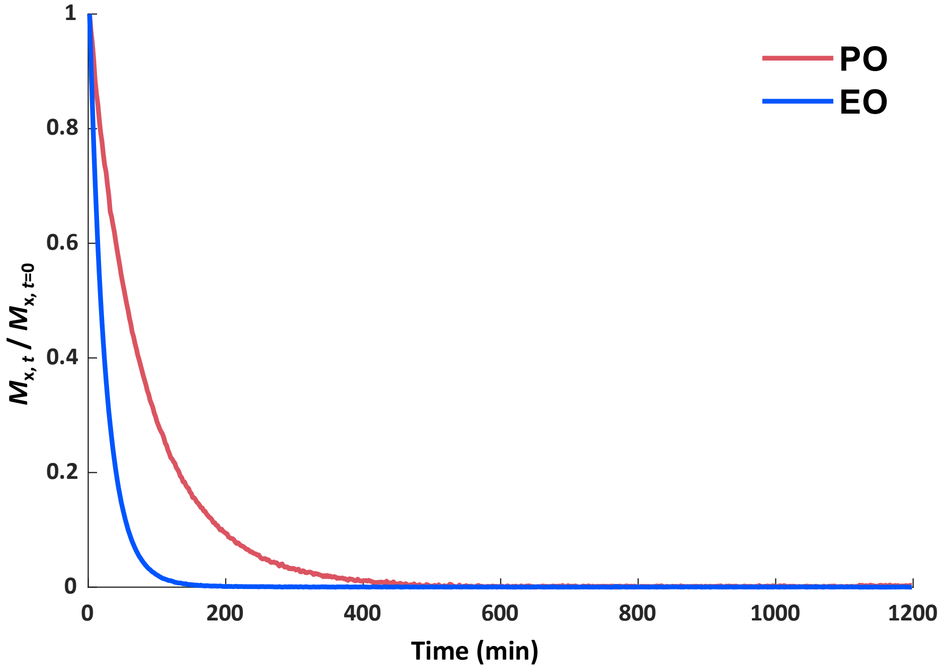

Figure S11: Time-conversion plot of the *in situ* ^{1}H NMR copolymerization kinetic study of EO with PO. (Solvent: DMSO-d_6, 40 °C).

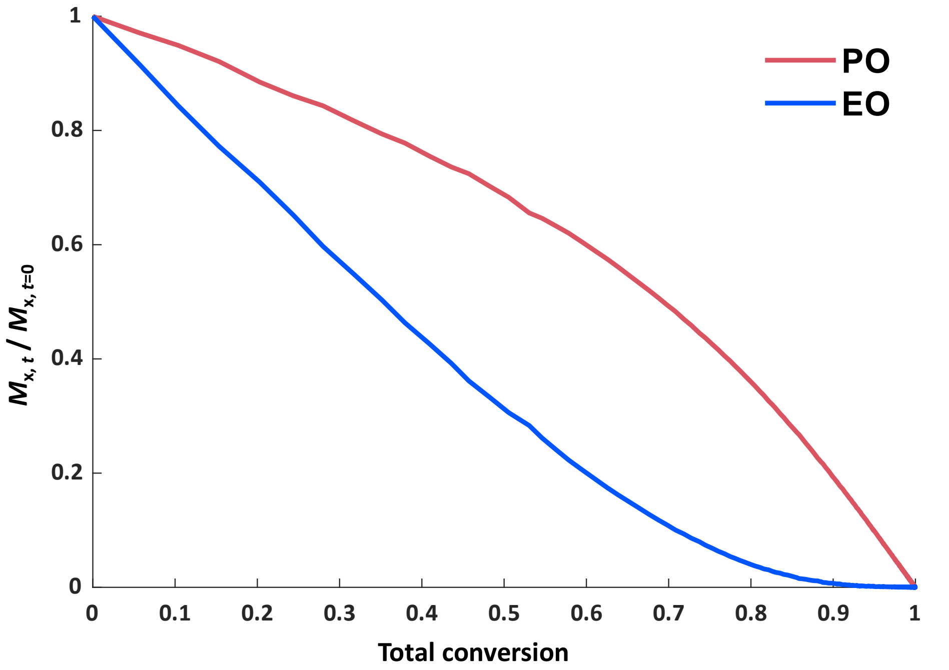

Figure S12: Individual versus total conversion of the *in situ* ^{1}H NMR copolymerization kinetic study of EO with PO. (Solvent: DMSO-d_6, 40 °C).

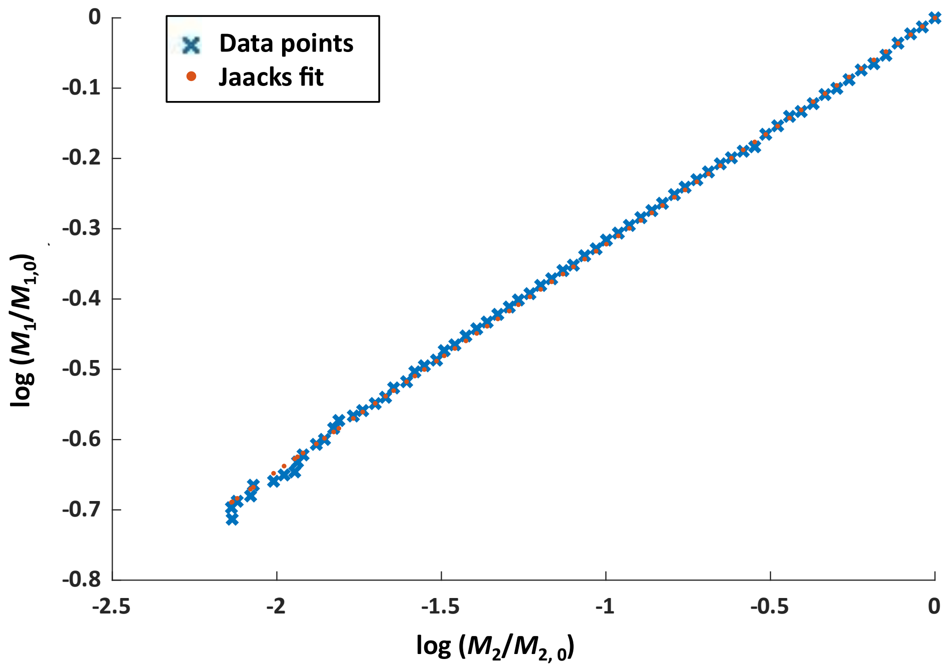

Figure S13: Jaacks fit of the *in situ* ^{1}H NMR copolymerization kinetic study of EO with PO. (Solvent: DMSO-d_6, 40 °C).

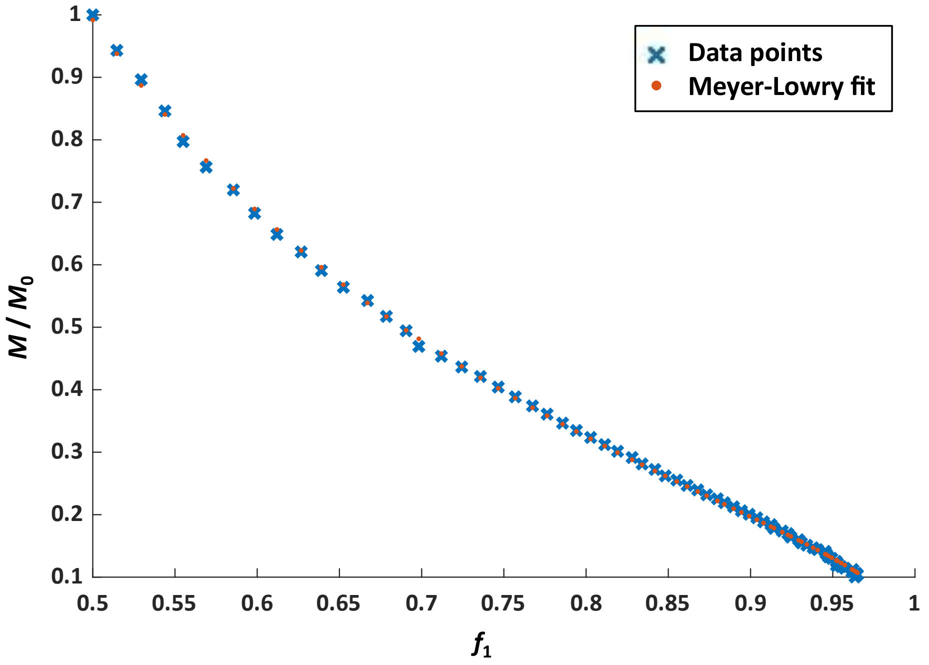

Figure S14: Meyer-Lowry fit of the *in situ* ^{1}H NMR copolymerization kinetic study of EO with PO. (Solvent: DMSO-d_6, 40 °C).

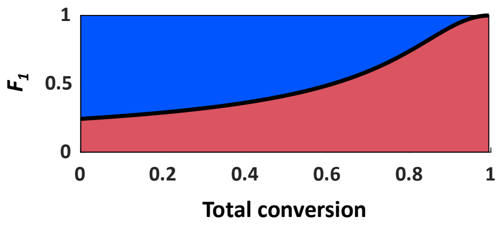

Figure S15: Composition plot of the *in situ* ^{1}H NMR copolymerization kinetic study of EO (blue) with PO (red) with a hypothetical equimolar monomer ratio (Solvent: DMSO-d_6, 40 °C) with *r*(PO)=0.32, *r*(EO)=3.10.

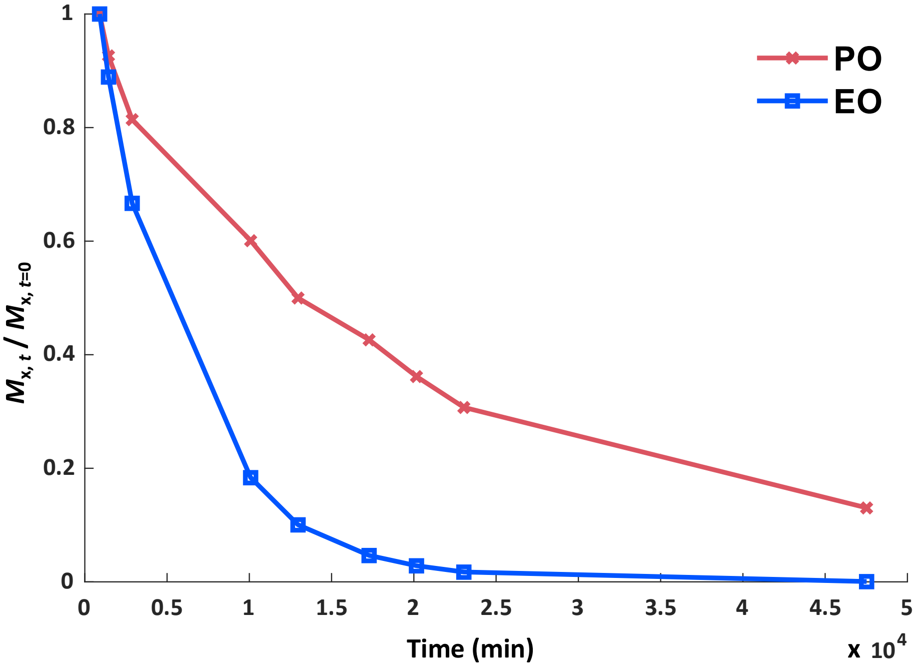

Figure S16: Time-conversion plot of the *in situ* ^{1}H NMR copolymerization kinetic study of EO with PO. (Solvent: Anisole, 25 °C). Data were acquired during an offline ^{1}H NMR kinetic experiment.

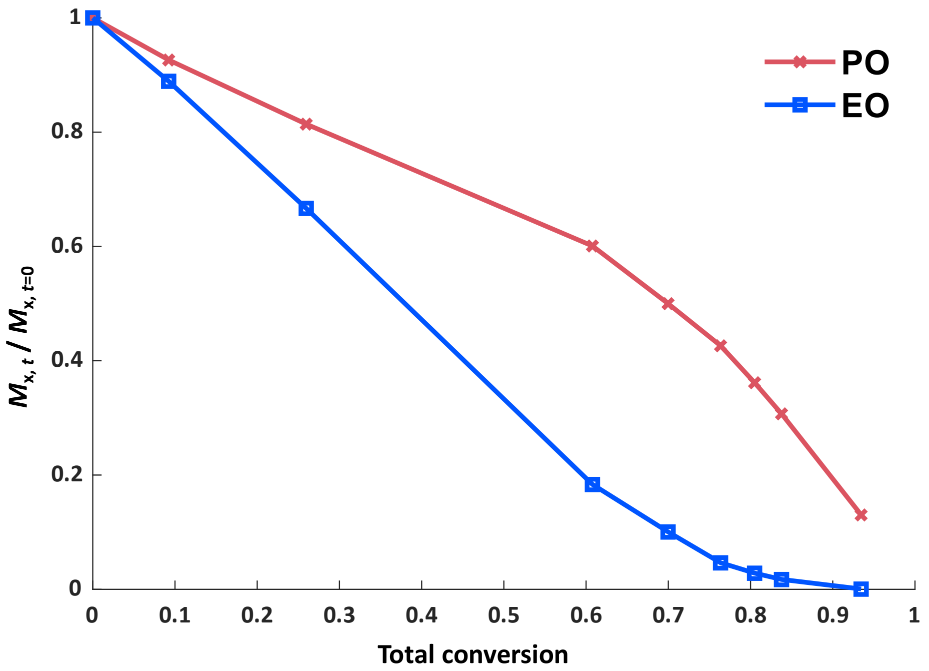

Figure S17: Individual versus total conversion of the *in situ* ^{1}H NMR copolymerization kinetic study of EO with PO. (Solvent: Anisole, 25 °C). Data were acquired during an offline ^{1}H NMR kinetic experiment.

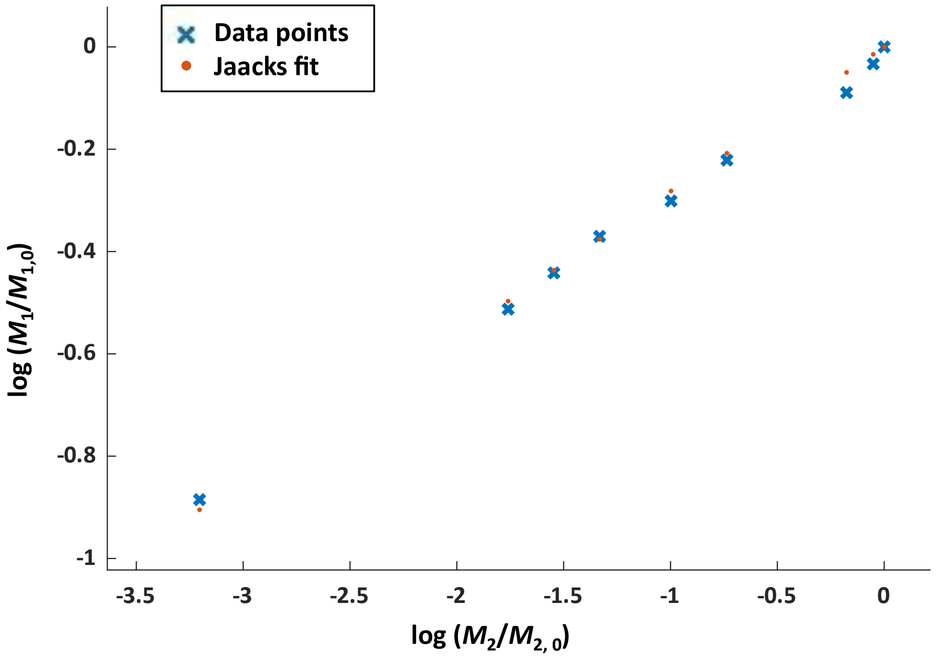

Figure S18: Jaacks fit of the *in situ* ^{1}H NMR copolymerization kinetic study of EO with PO. (Solvent: Anisole, 25 °C). Data were acquired during an offline ^{1}H NMR kinetic experiment.

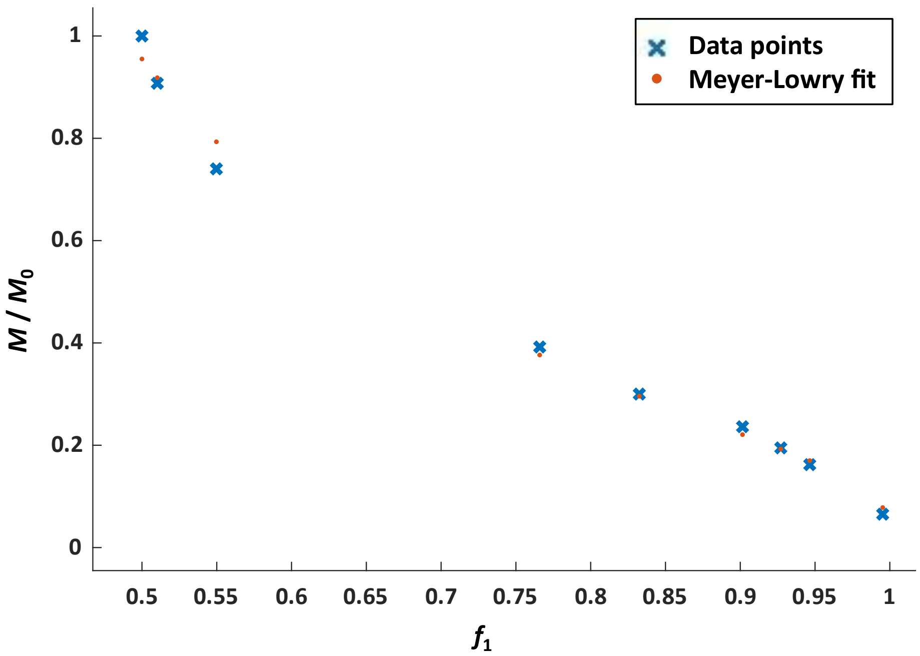

Figure S19: Meyer-Lowry fit of the *in situ* ^{1}H NMR copolymerization kinetic study of EO with PO. (Solvent: Anisole, 25 °C). Data were acquired during an offline ^{1}H NMR kinetic experiment.

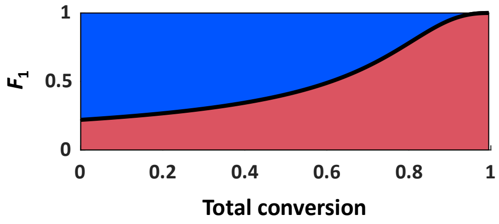

Figure S20: Composition plot of the *in situ* ^{1}H NMR copolymerization kinetic study of EO (blue) with PO (red) with a hypothetical equimolar monomer ratio (Solvent: Anisole, 25 °C) with *r*(PO)=0.28, *r*(EO)=3.54.

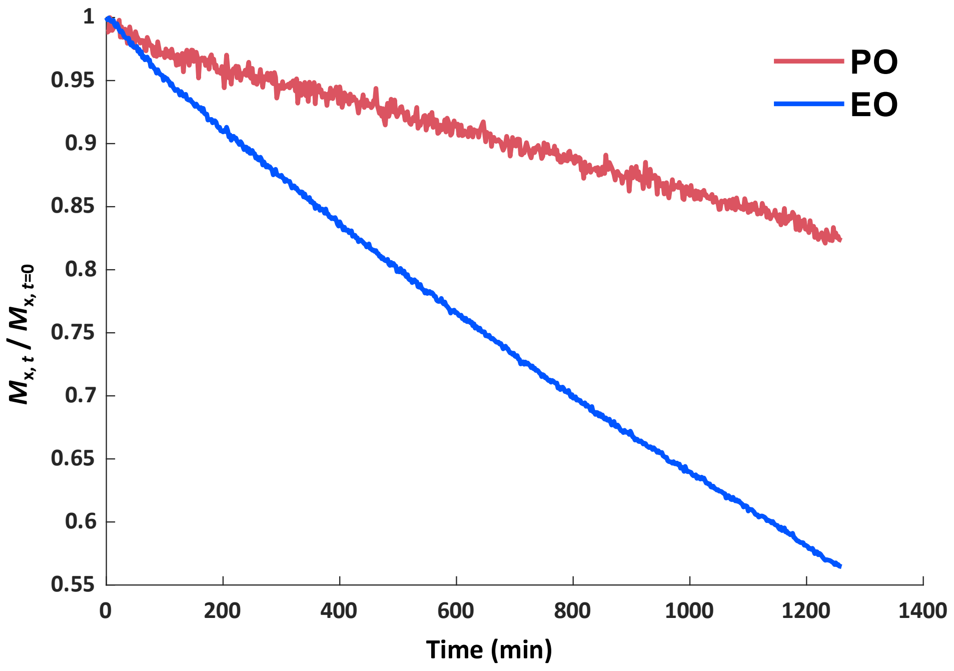

Figure S21: Time-conversion plot of the *in situ* ^{1}H NMR copolymerization kinetic study of EO with PO. (Solvent: Anisole, 25 °C, addition of [18]crown-6).

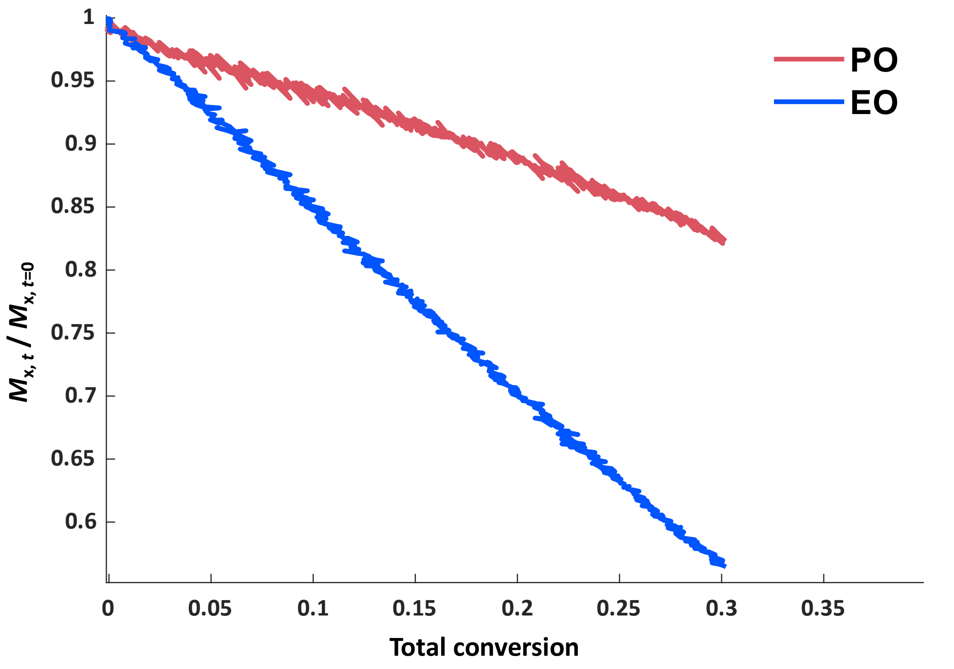

Figure S22: Individual versus total conversion of the *in situ* ^{1}H NMR copolymerization kinetic study of EO with PO. (Solvent: Anisole, 25 °C, addition of [18]crown-6).

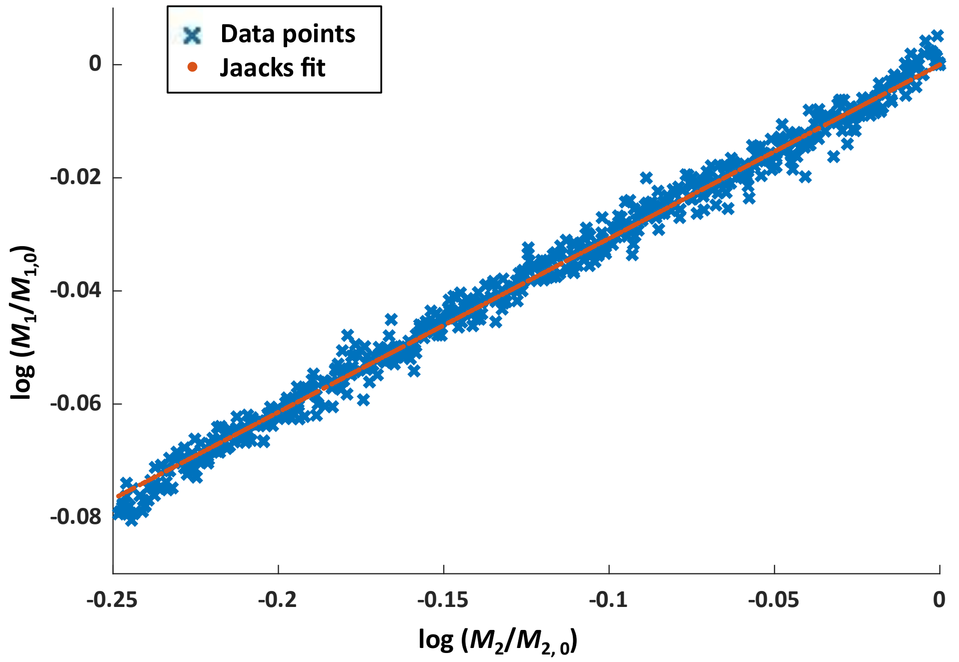

Figure S23: Jaacks fit of the *in situ* ^{1}H NMR copolymerization kinetic study of EO with PO. (Solvent: Anisole, 25 °C, addition of [18]crown-6).

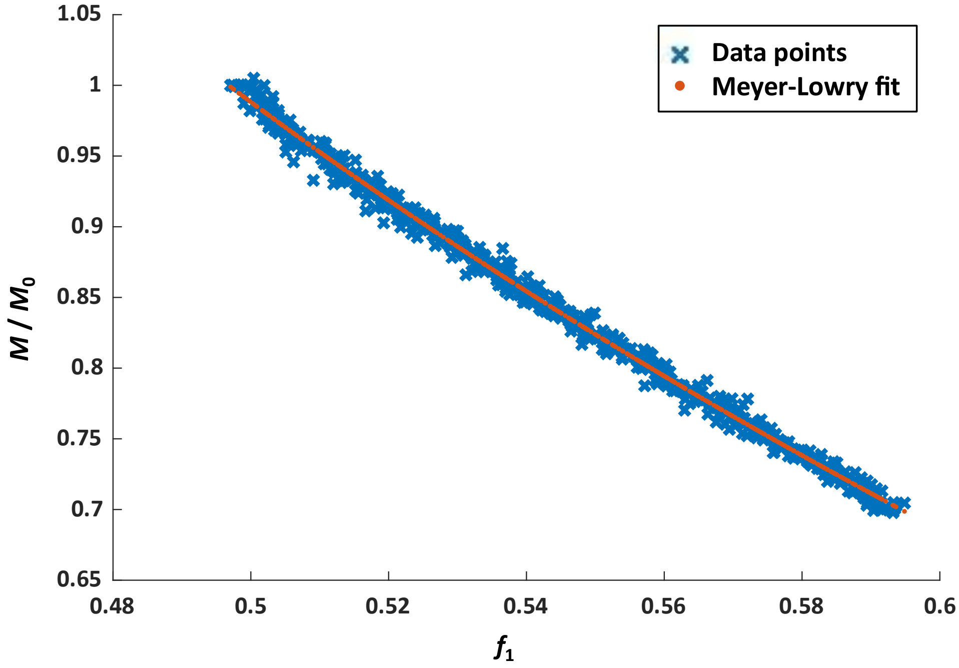

Figure S24: Meyer-Lowry fit of the *in situ* ^{1}H NMR copolymerization kinetic study of EO with PO. (Solvent: Anisole, 25 °C, addition of [18]crown-6).

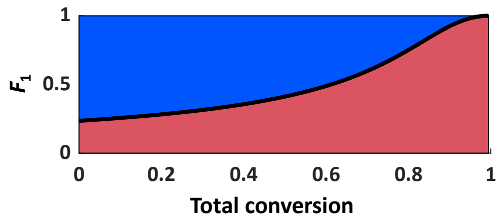

Figure S25: Composition plot of the *in situ* ^{1}H NMR copolymerization kinetic study of EO (blue) with PO (red) with a hypothetical equimolar monomer ratio (Solvent: Anisole, 25 °C, addition of [18]crown-6) with *r*(PO)=0.31, *r*(EO)=3.26.

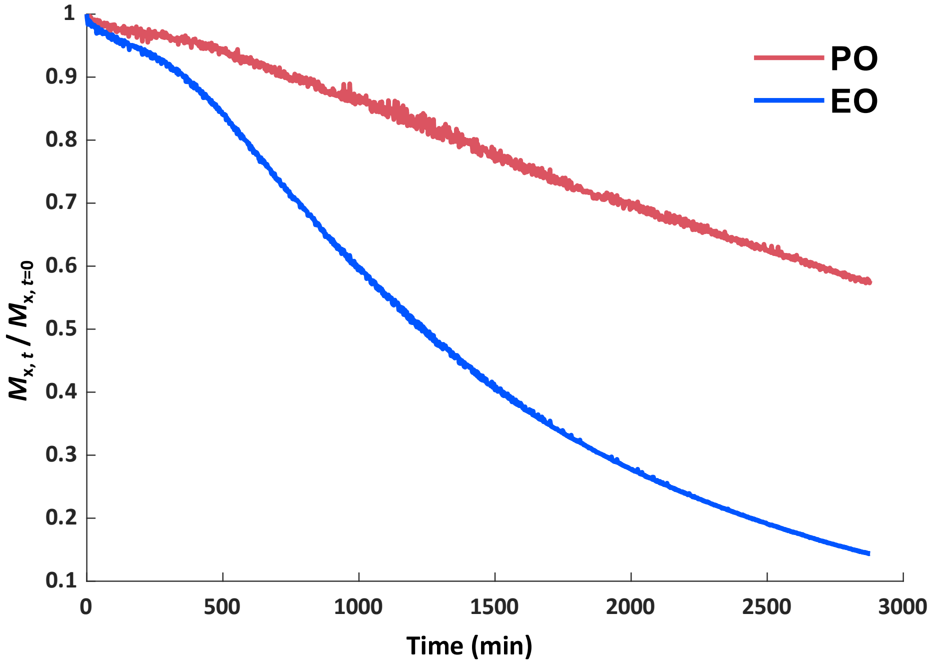

Figure S26: Time-conversion plot of the *in situ* ^{1}H NMR copolymerization kinetic study of EO with PO. (Solvent: Anisole, 40 °C).

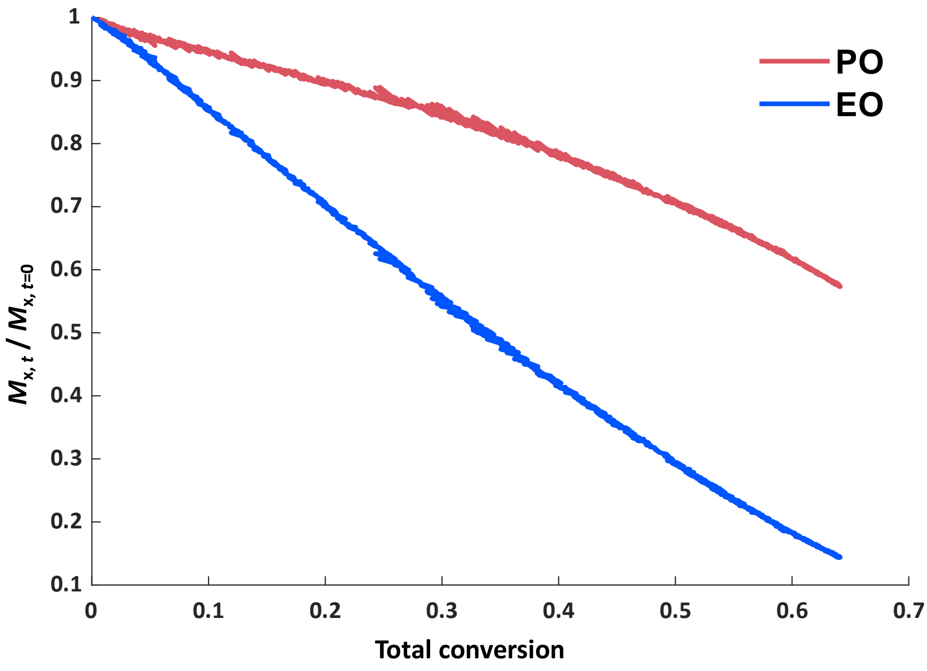

Figure S27: Individual versus total conversion of the *in situ* ^{1}H NMR copolymerization kinetic study of EO with PO. (Solvent: Anisole, 40 °C).

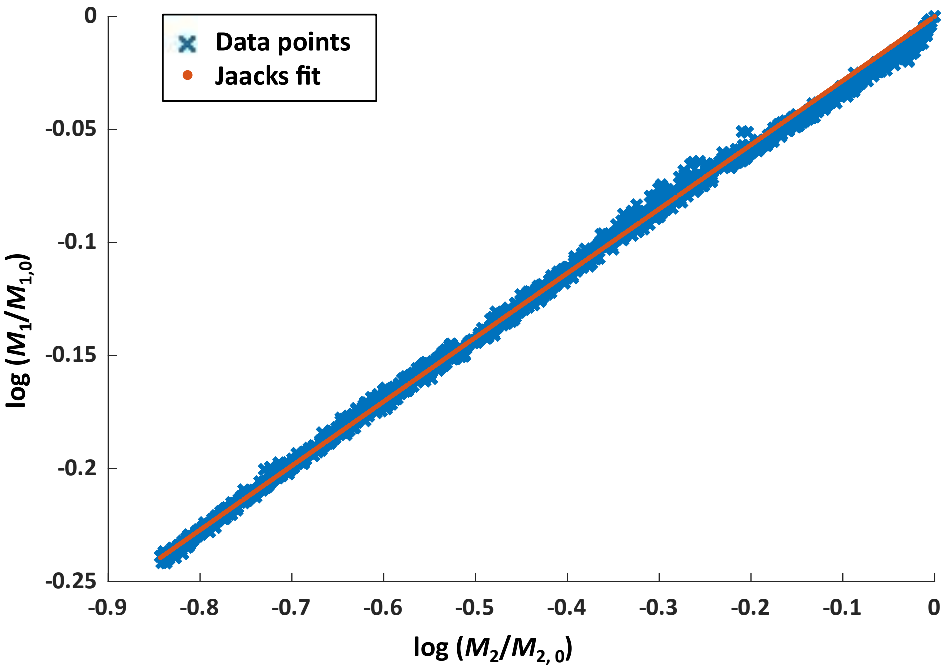

Figure S28: Jaacks fit of the *in situ* ^{1}H NMR copolymerization kinetic study of EO with PO. (Solvent: Anisole, 40 °C).

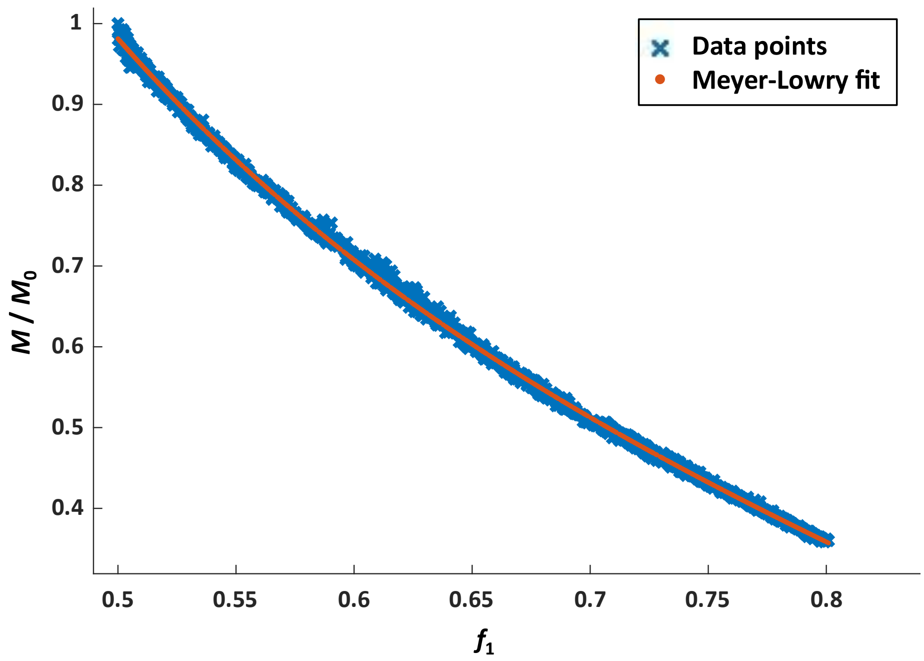

Figure S29: Meyer-Lowry fit of the *in situ* ^{1}H NMR copolymerization kinetic study of EO with PO. (Solvent: Anisole, 40 °C).

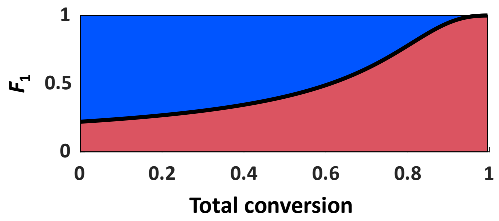

Figure S30: Composition plot of the *in situ* ^{1}H NMR copolymerization kinetic study of EO (blue) with PO (red) with a hypothetical equimolar monomer ratio (Solvent: Anisole, 40 °C) with *r*(PO)=0.28, *r*(EO)=3.52.

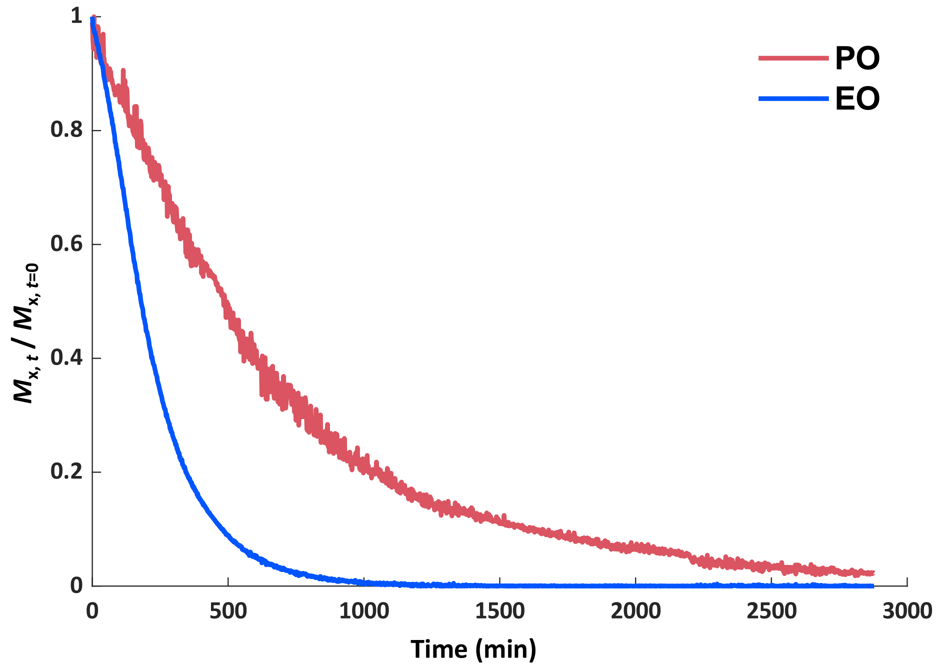

Figure S31: Time-conversion plot of the *in situ* ^{1}H NMR copolymerization kinetic study of EO with PO. (Solvent: Anisole, 60 °C).

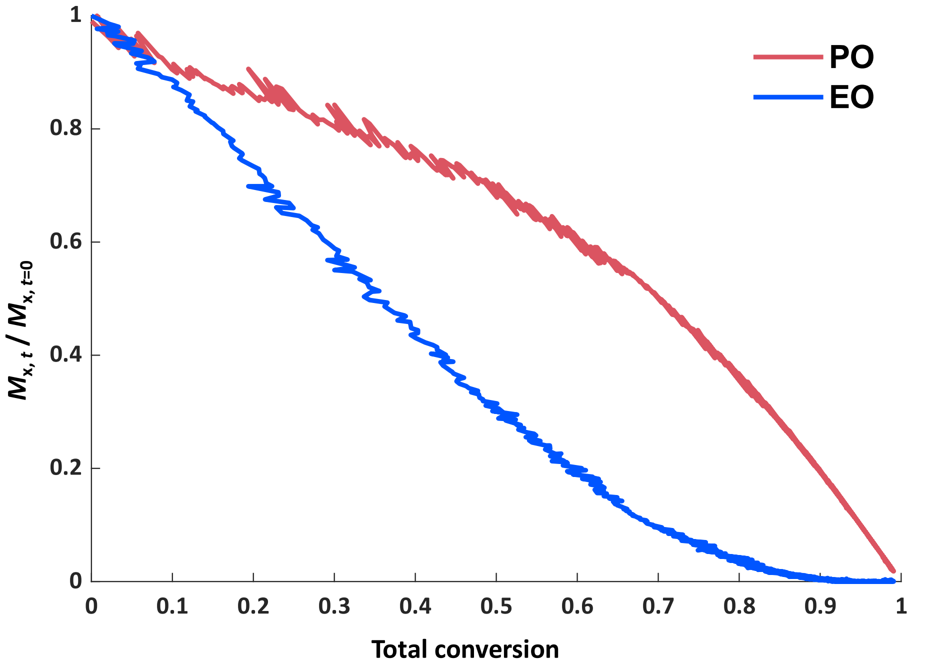

Figure S32: Individual versus total conversion of the *in situ* ^{1}H NMR copolymerization kinetic study of EO with PO. (Solvent: Anisole, 60 °C).

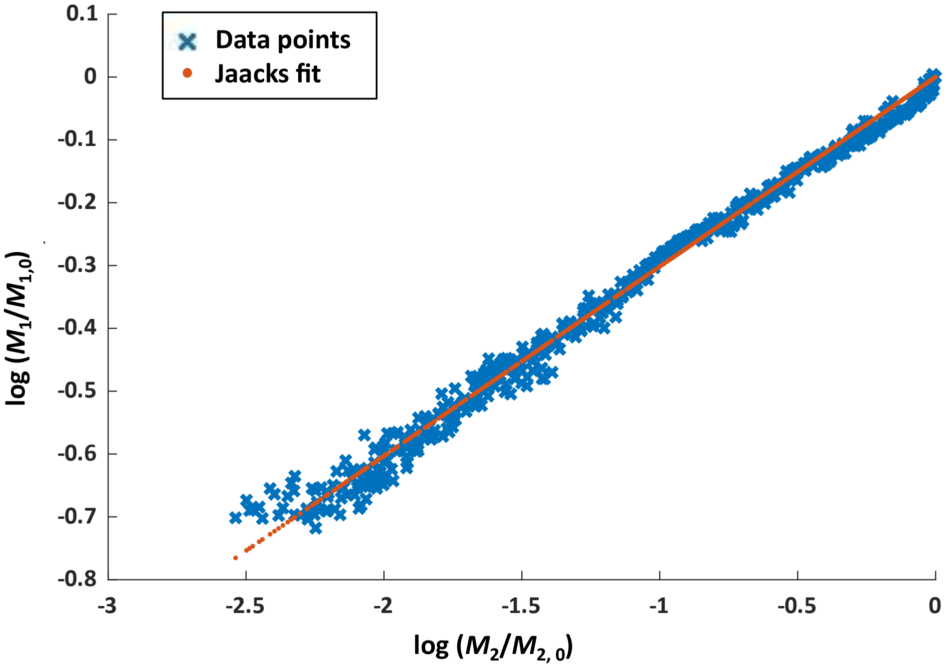

Figure S33: Jaacks fit of the *in situ* ^{1}H NMR copolymerization kinetic study of EO with PO. (Solvent: Anisole, 60 °C).

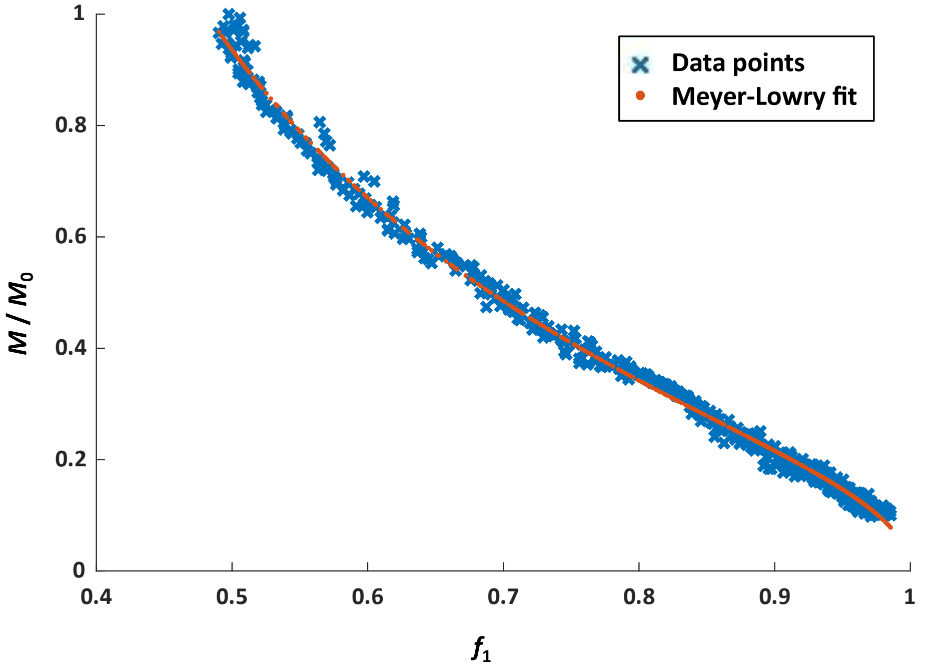

Figure S34: Meyer-Lowry fit of the *in situ* ^{1}H NMR copolymerization kinetic study of EO with PO. (Solvent: Anisole, 60 °C).

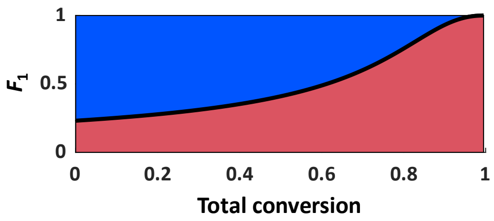

Figure S35: Composition plot of the *in situ* ^{1}H NMR copolymerization kinetic study of EO (blue) with PO (red) with a hypothetical equimolar monomer ratio (Solvent: Anisole, 60 °C) with *r*(PO)=0.30, *r*(EO)=3.32.

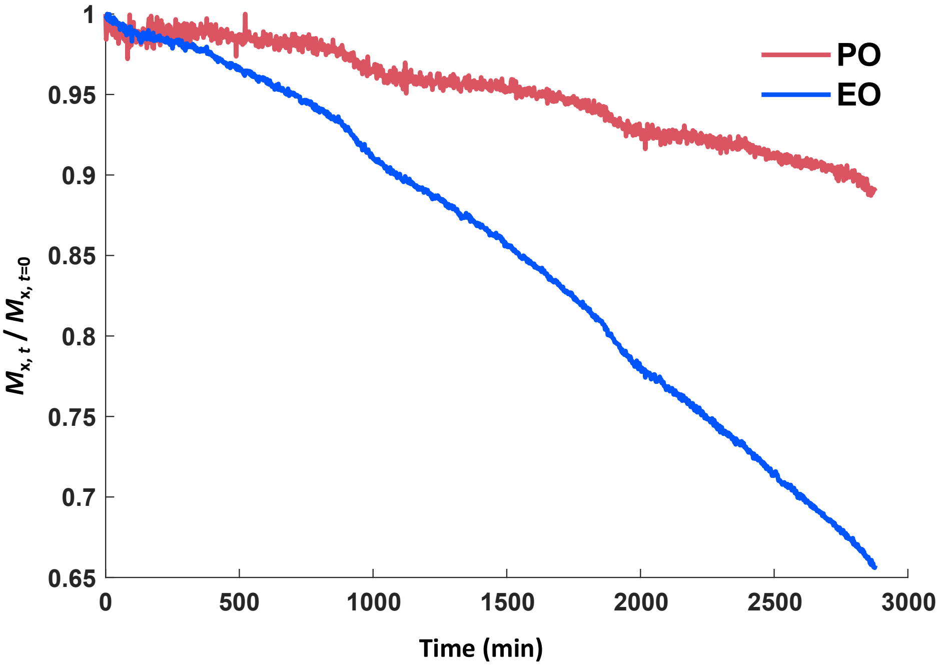

Figure S36: Time-conversion plot of the *in situ* ^{1}H NMR copolymerization kinetic study of EO with PO. (Solvent: Toluene-d_8, 25 °C).

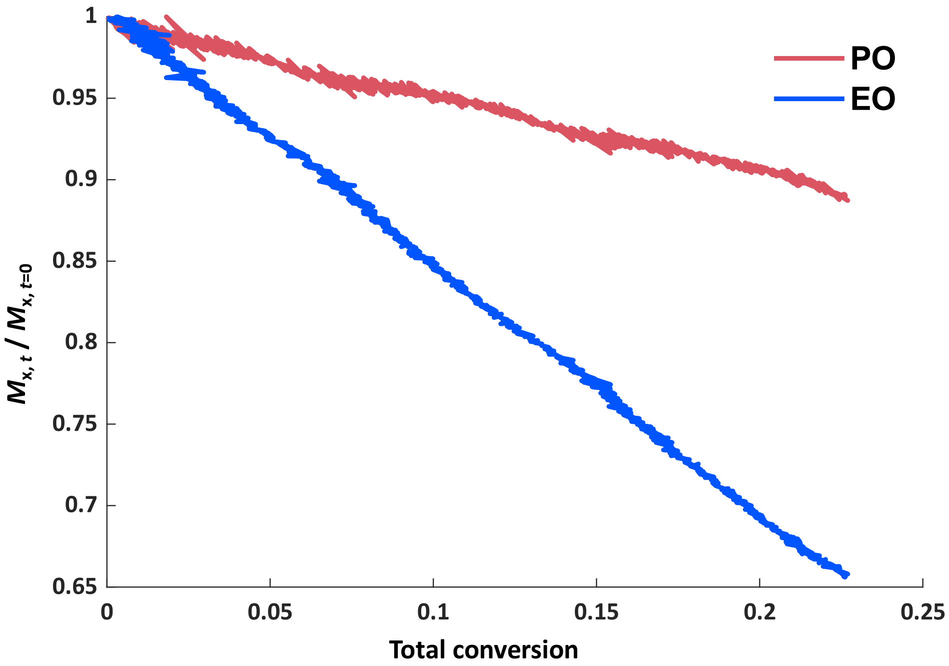

Figure S37: Individual versus total conversion of the *in situ* ^{1}H NMR copolymerization kinetic study of EO with PO. (Solvent: Toluene-d_8, 25 °C).

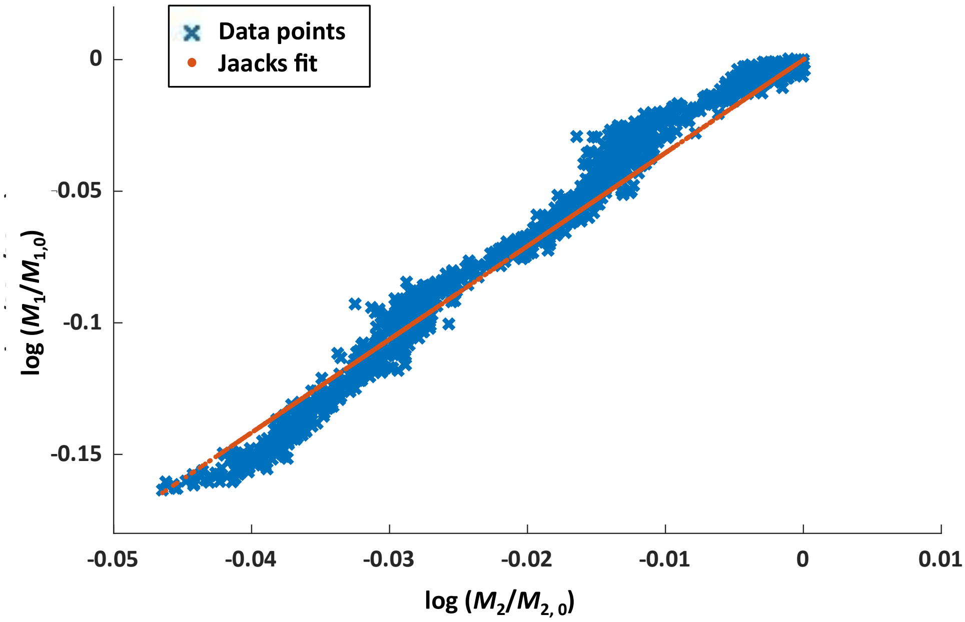

Figure S38: Jaacks fit of the *in situ* ^{1}H NMR copolymerization kinetic study of EO with PO. (Solvent: Toluene-d_8, 25 °C).

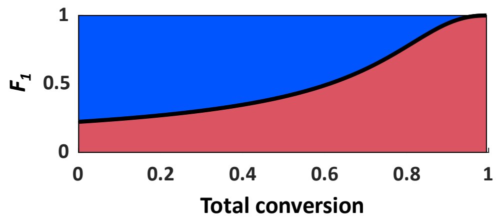

Figure S39: Composition plot of the *in situ* ^{1}H NMR copolymerization kinetic study of EO (blue) with PO (red) with a hypothetical equimolar monomer ratio (Solvent: Toluene-d_8, 25 °C) with *r*(PO)=0.29, *r*(EO)=3.49.

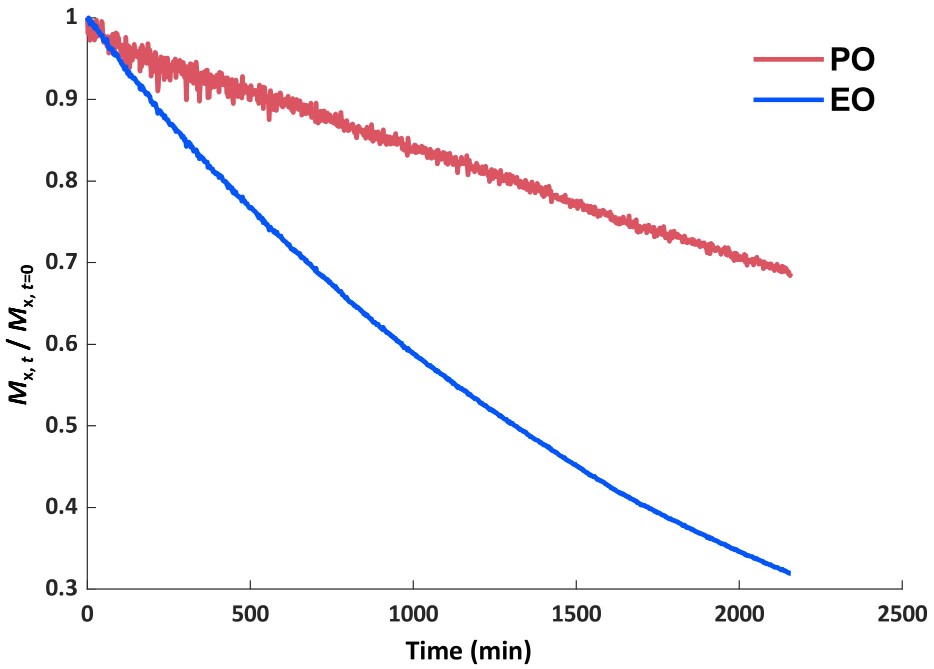

Figure S40: Time-conversion plot of the *in situ* ^{1}H NMR copolymerization kinetic study of EO with PO. (Solvent: Toluene-d_8, 25 °C, addition of [18]crown-6).

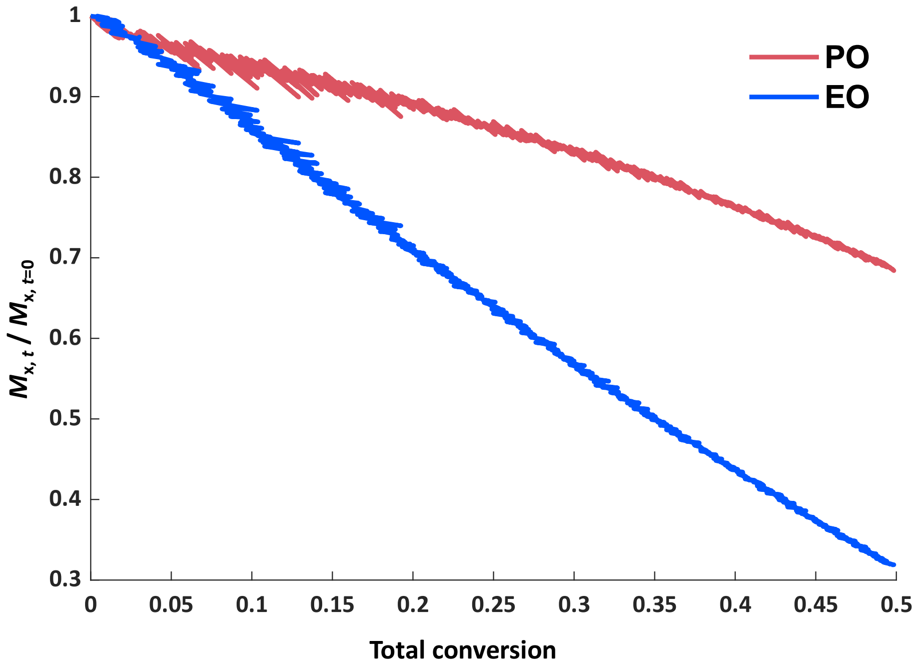

Figure S41: Individual versus total conversion of the *in situ* ^{1}H NMR copolymerization kinetic study of EO with PO. (Solvent: Toluene-d_8, 25 °C, addition of [18]crown-6).

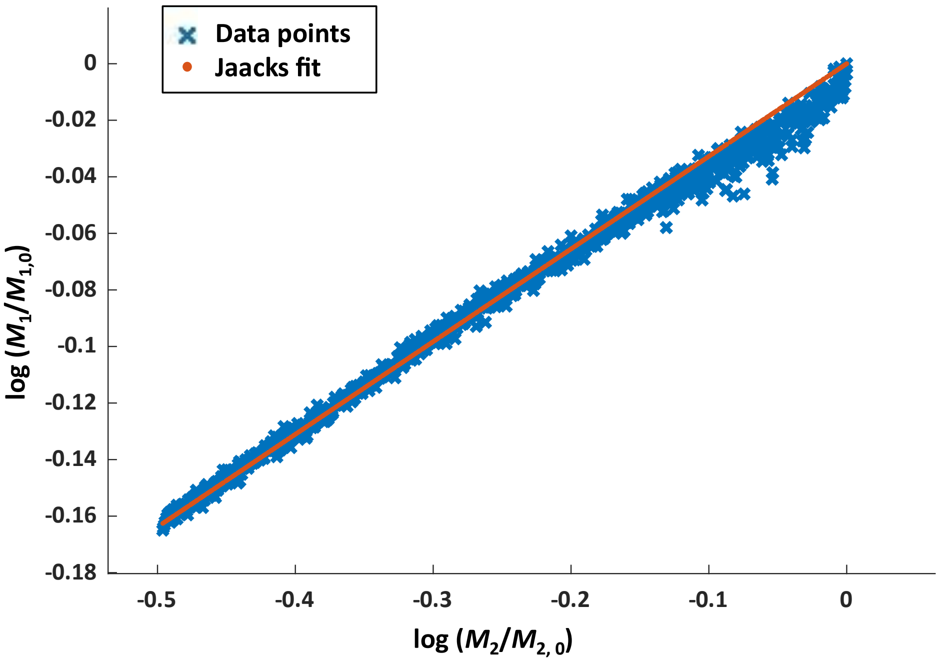

Figure S42: Jaacks fit of the *in situ* ^{1}H NMR copolymerization kinetic study of EO with PO. (Solvent: Toluene-d_8, 25 °C, addition of [18]crown-6).

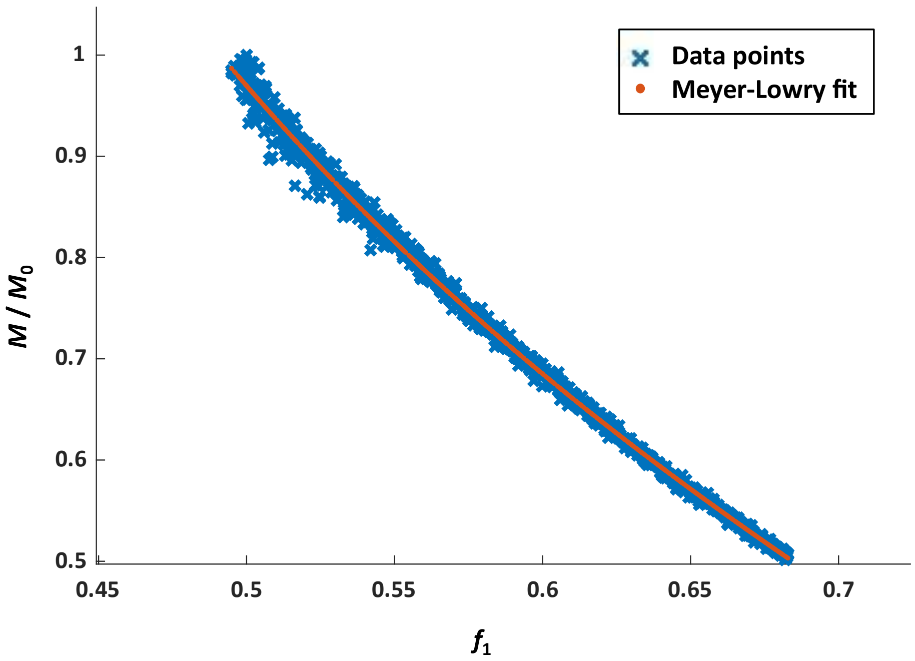

Figure S43: Meyer-Lowry fit of the *in situ* ^{1}H NMR copolymerization kinetic study of EO with PO. (Solvent: Toluene-d_8, 25 °C, addition of [18]crown-6).

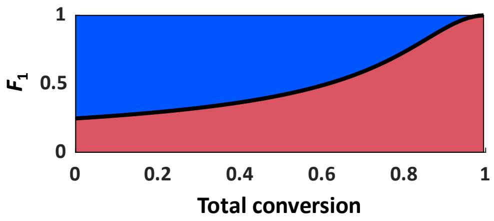

Figure S44: Composition plot of the *in situ* ^{1}H NMR copolymerization kinetic study of EO (blue) with PO (red) with a hypothetical equimolar monomer ratio (Solvent: Toluene-d_8, 25 °C, addition of [18]crown-6) with *r*(PO)=0.33, *r*(EO)=3.05.

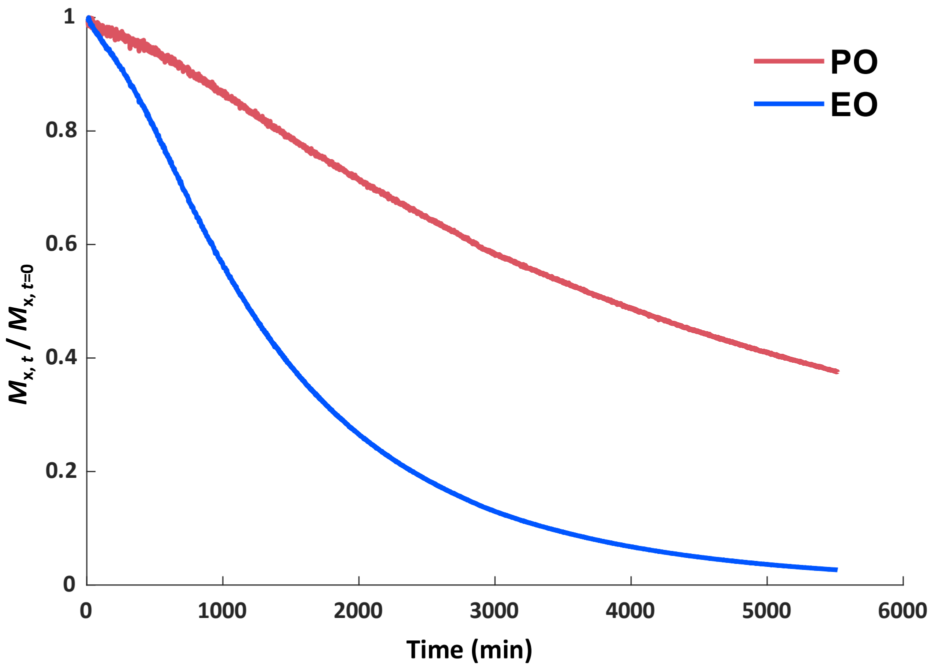

Figure S45: Time-conversion plot of the *in situ* ^{1}H NMR copolymerization kinetic study of EO with PO. (Solvent: Toluene-d_8, 40 °C).

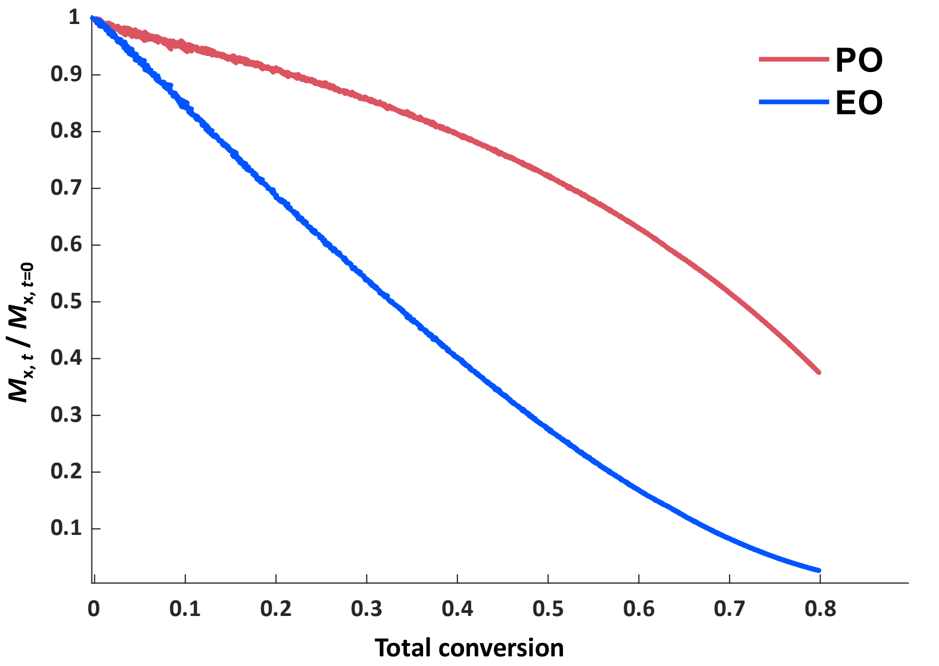

Figure S46: Individual versus total conversion of the *in situ* ^{1}H NMR copolymerization kinetic study of EO with PO. (Solvent: Toluene-d_8, 40 °C).

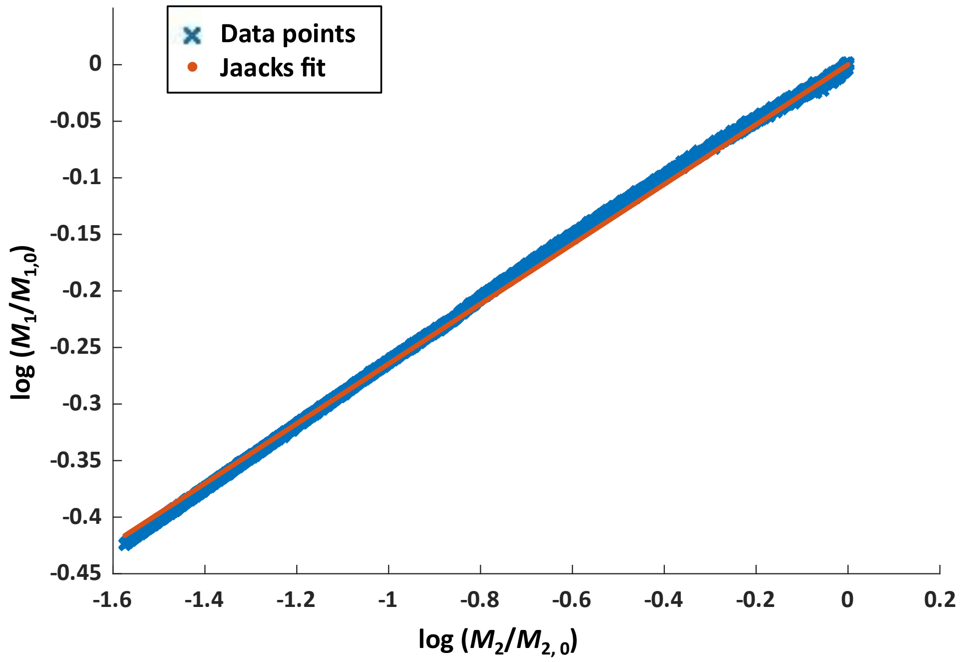

Figure S47: Jaacks fit of the *in situ* ^{1}H NMR copolymerization kinetic study of EO with PO. (Solvent: Toluene-d_8, 40 °C).

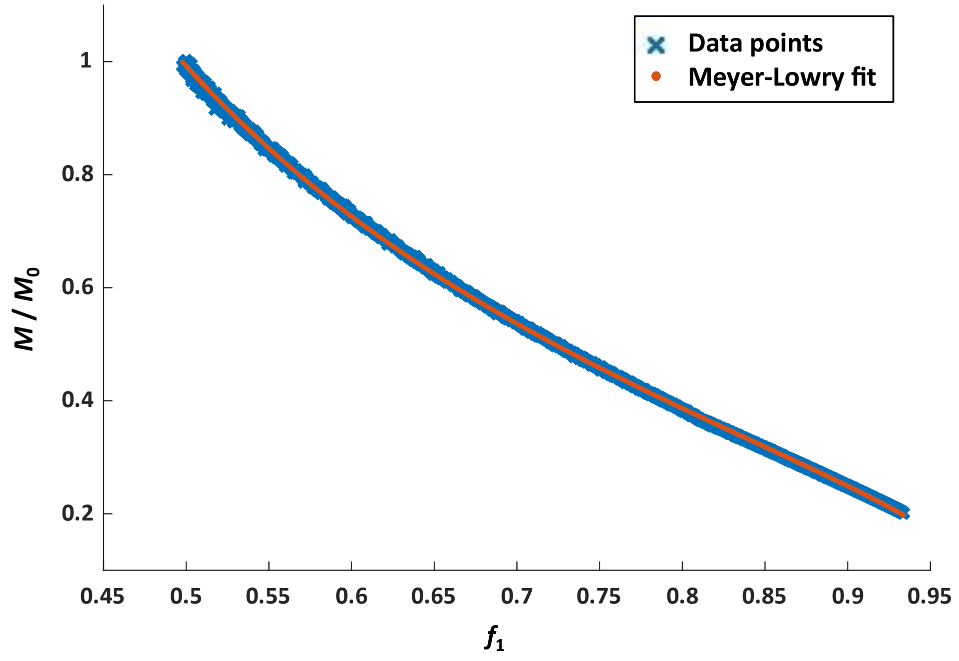

Figure S48: Meyer-Lowry fit of the *in situ* ^{1}H NMR copolymerization kinetic study of EO with PO. (Solvent: Toluene-d_8, 40 °C).

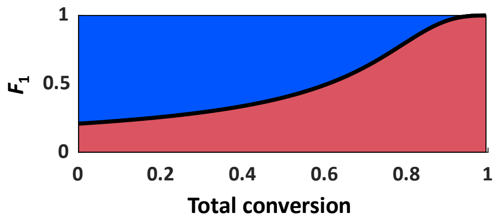

Figure S49: Composition plot of the *in situ* ^{1}H NMR copolymerization kinetic study of EO (blue) with PO (red) with a hypothetical equimolar monomer ratio (Solvent: Toluene-d_8, 40 °C) with *r*(PO)=0.26, *r*(EO)=3.78.

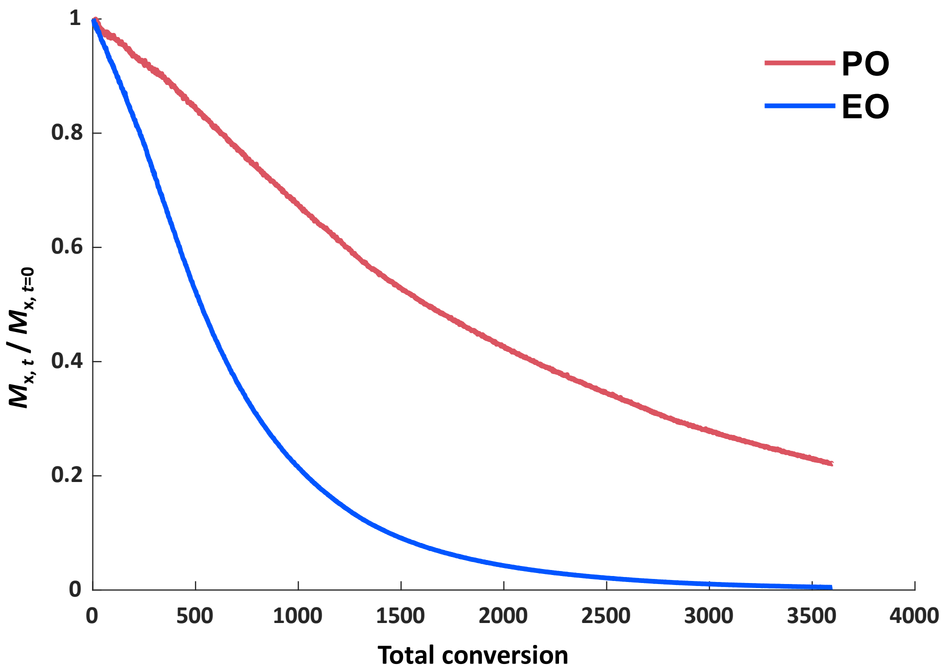

Figure S50: Time-conversion plot of the *in situ* ^{1}H NMR copolymerization kinetic study of EO with PO. (Solvent: Toluene-d_8, 50 °C).

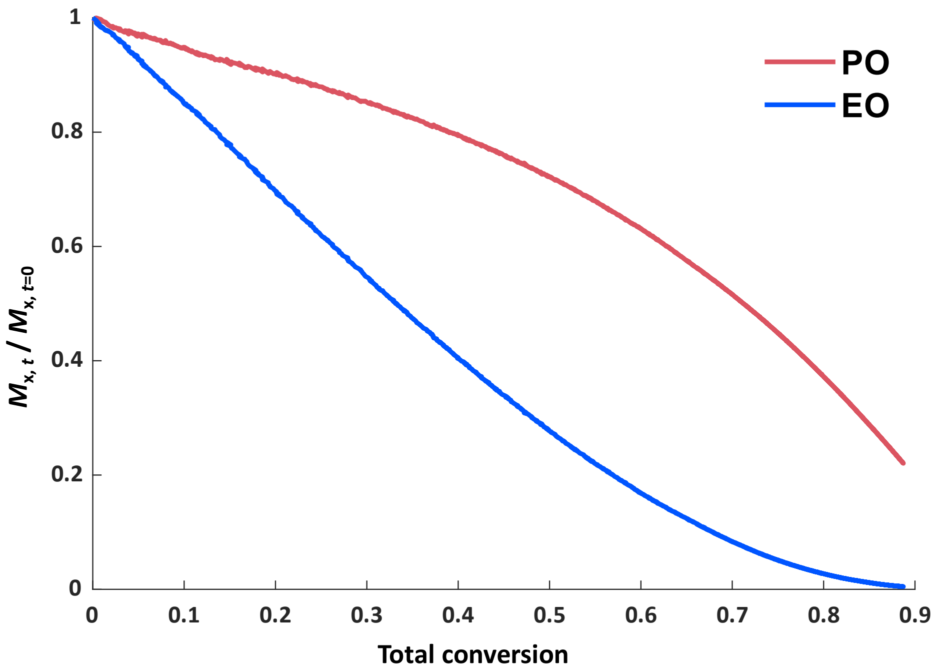

Figure S51: Individual versus total conversion of the *in situ* ^{1}H NMR copolymerization kinetic study of EO with PO. (Solvent: Toluene-d_8, 50 °C).

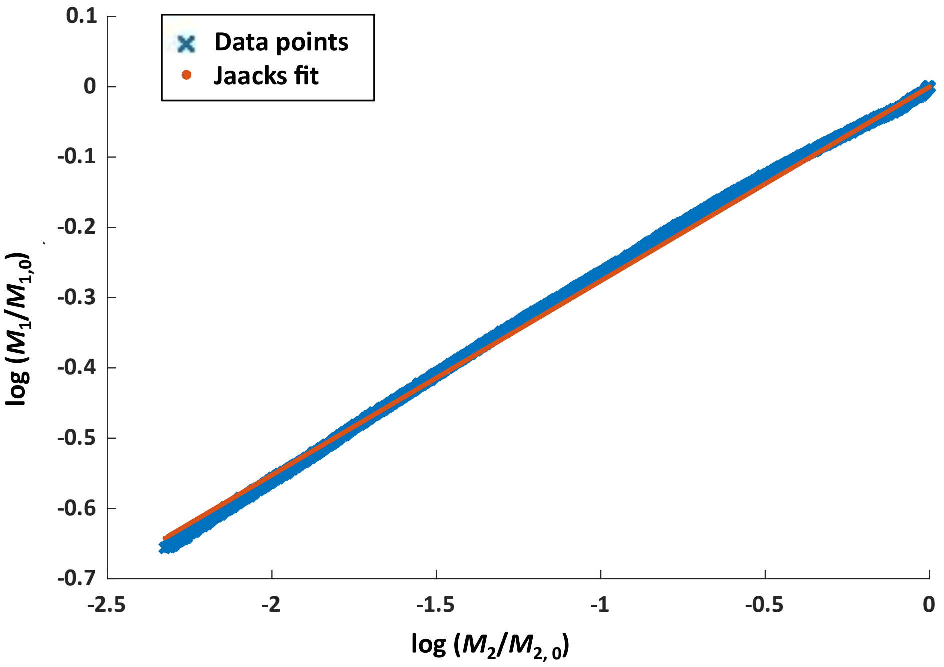

Figure S52: Jaacks fit of the *in situ* ^{1}H NMR copolymerization kinetic study of EO with PO. (Solvent: Toluene-d_8, 50 °C).

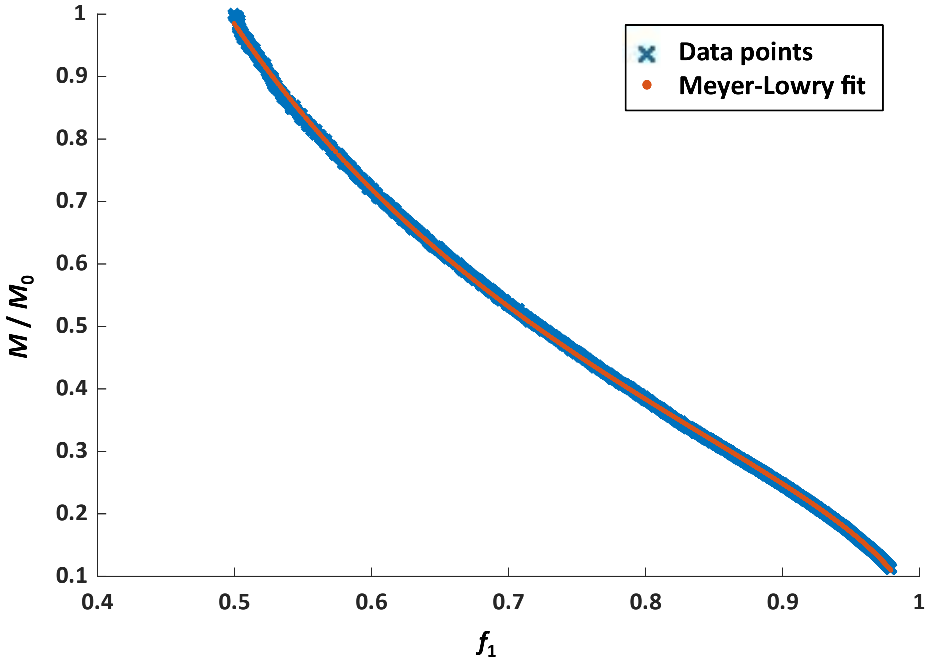

Figure S53: Meyer-Lowry fit of the *in situ* ^{1}H NMR copolymerization kinetic study of EO with PO. (Solvent: Toluene-d_8, 50 °C).

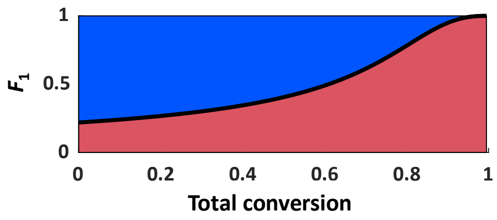

Figure S54: Composition plot of the *in situ* ^{1}H NMR copolymerization kinetic study of EO (blue) with PO (red) with a hypothetical equimolar monomer ratio (Solvent: Toluene-d_8, 50 °C) with *r*(PO)=0.28, *r*(EO)=3.62.

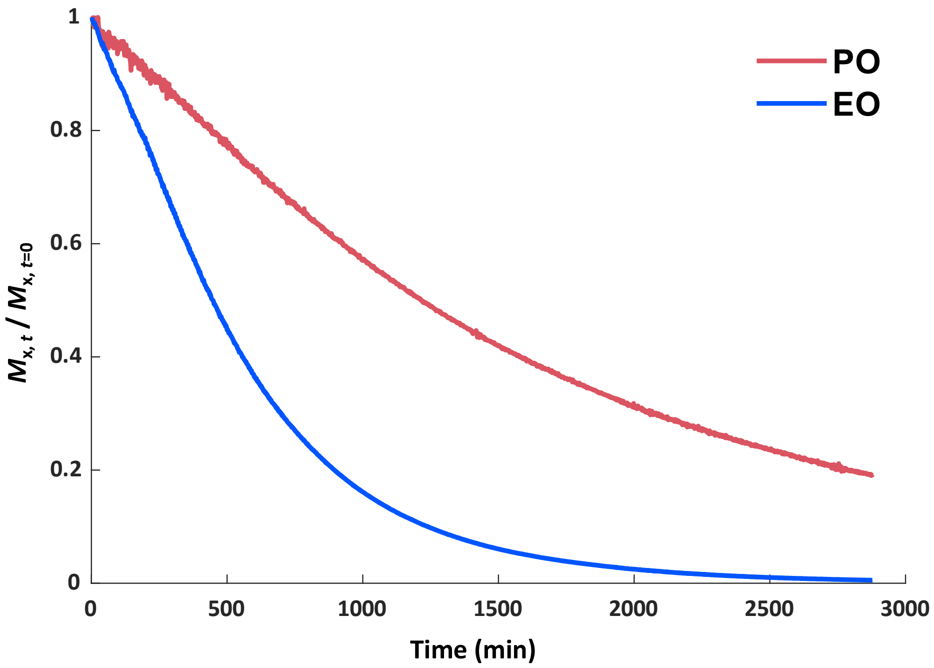

Figure S55: Time-conversion plot of the *in situ* ^{1}H NMR copolymerization kinetic study of EO with PO. (Solvent: Toluene-d_8, 60 °C).

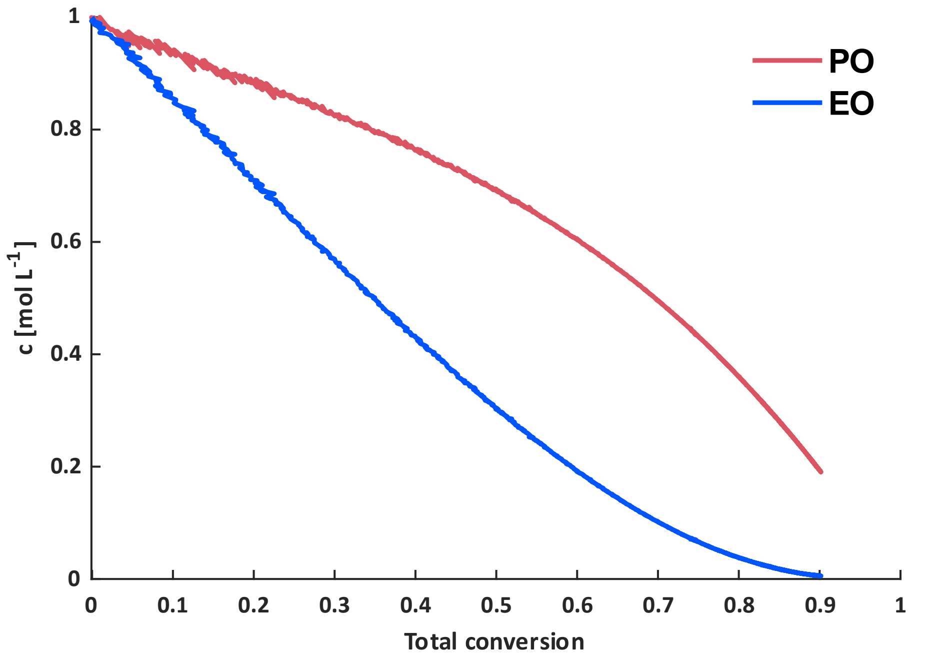

Figure S56: Individual versus total conversion of the *in situ* ^{1}H NMR copolymerization kinetic study of EO with PO. (Solvent: Toluene-d_8, 60 °C).

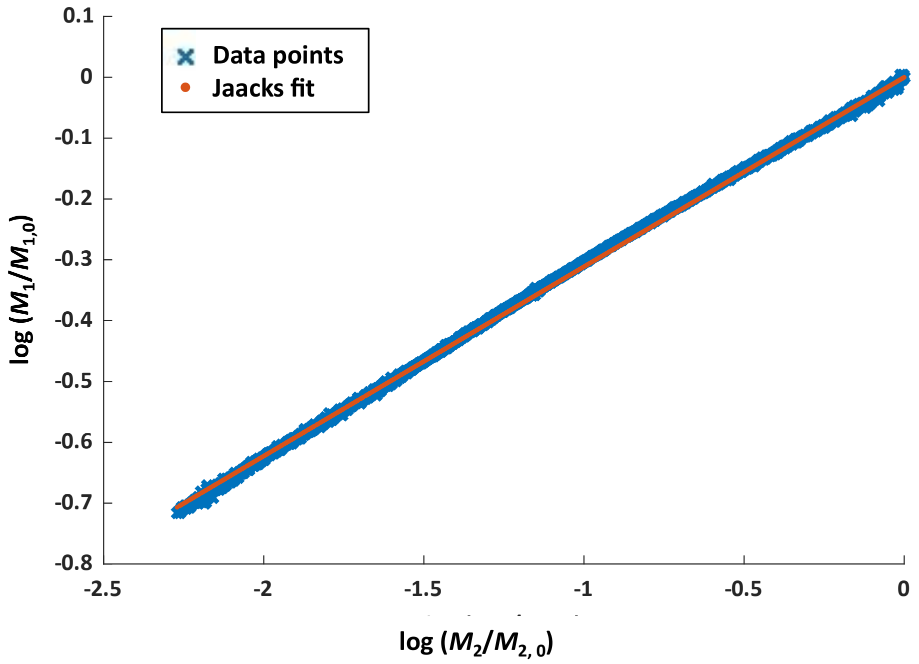

Figure S57: Jaacks fit of the *in situ* ^{1}H NMR copolymerization kinetic study of EO with PO. (Solvent: Toluene-d_8, 60 °C).

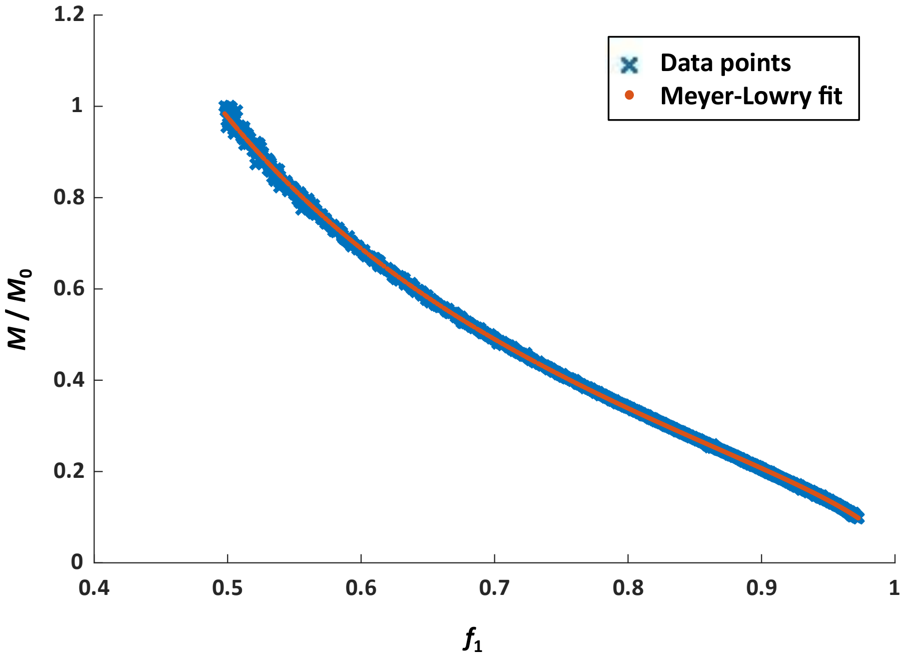

Figure S58: Meyer-Lowry fit of the *in situ* ^{1}H NMR copolymerization kinetic study of EO with PO. (Solvent: Toluene-d_8, 60 °C).

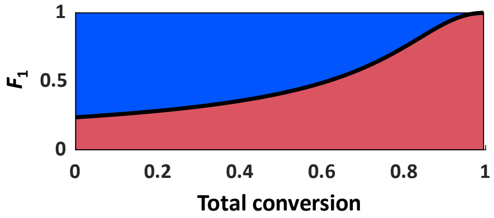

Figure S59: Composition plot of the *in situ* ^{1}H NMR copolymerization kinetic study of EO (blue) with PO (red) with a hypothetical equimolar monomer ratio (Solvent: Toluene-d_8, 60 °C) with *r*(PO)=0.31, *r*(EO)=3.21.

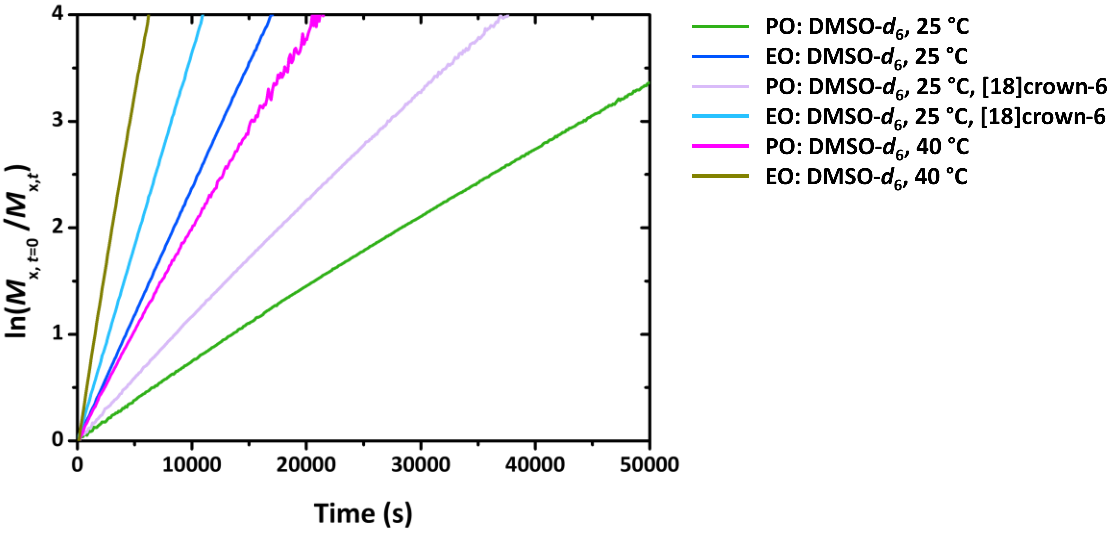

Figure S60: Pseudo-first-order plot of the copolymerization of EO with PO in DMSO at different conditions obtained by ^{1}H NMR kinetics.

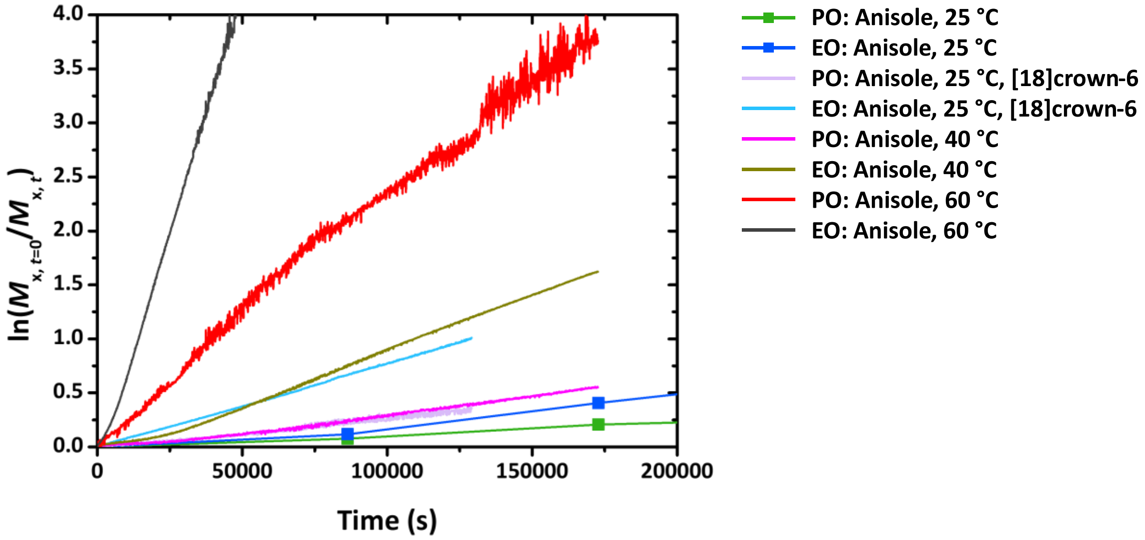

Figure S61: Pseudo-first-order plot of the copolymerization of EO with PO in Anisole at different conditions obtained by ^{1}H NMR kinetics. Anisole measurements at 25 °C without [18]crown-6 were acquired offline, data points are marked by squares.

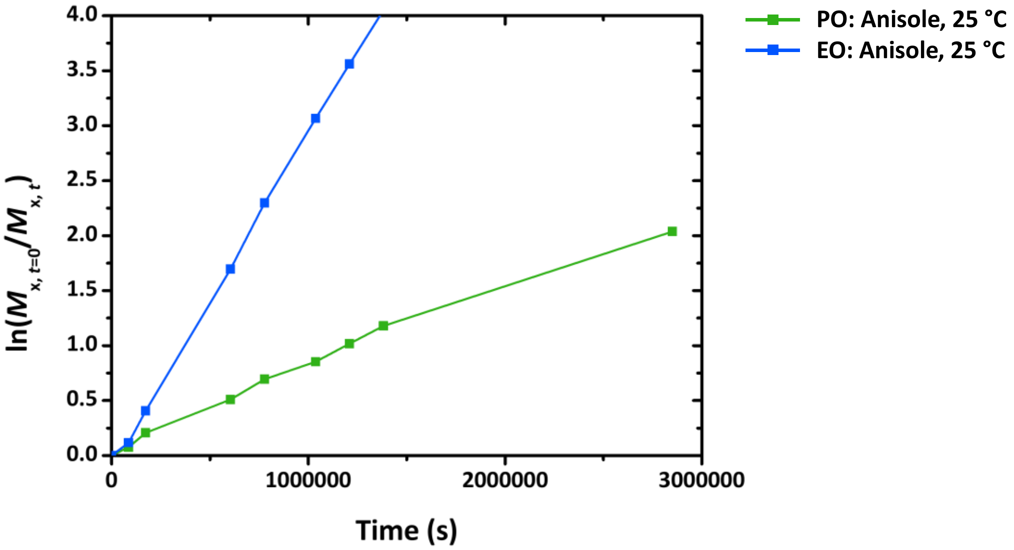

Figure S62: Pseudo-first-order plot of the copolymerization of EO with PO in Anisole at 25 °C obtained by offline NMR kinetics. Data points are marked by squares.

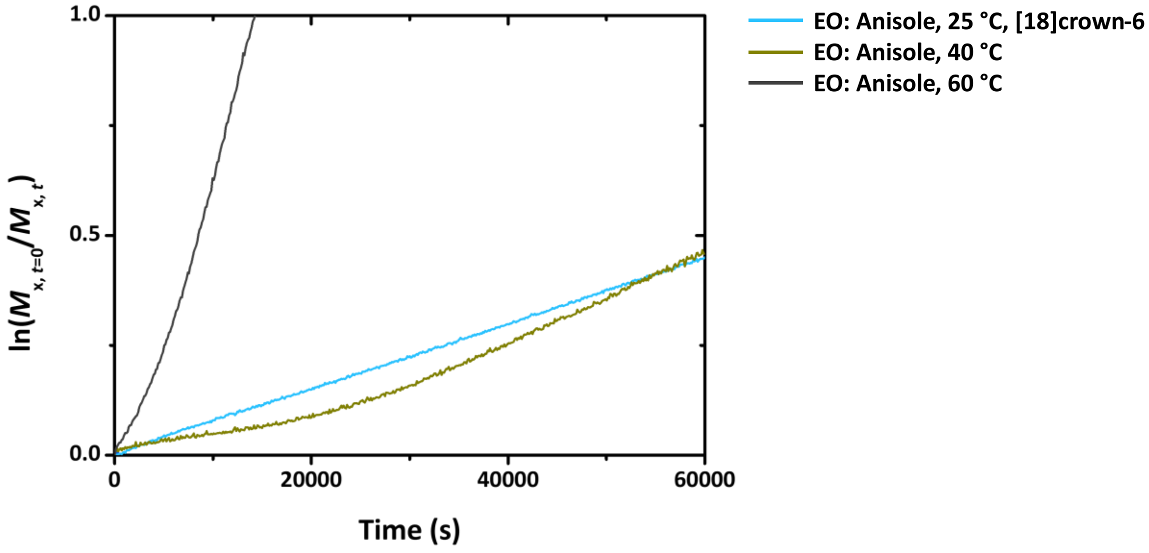

Figure S63: Enlargement of the pseudo-first-order plot of the copolymerization of EO with PO in Anisole at different conditions obtained by ^{1}H NMR kinetics. The enlarged start period shows the induction of the EO signal. The PO graphs are omitted for clarity reasons. The polymerization condition of Anisole at 25 °C without [18]crown-6 was omitted for scale reasons and is displayed in the following graph.

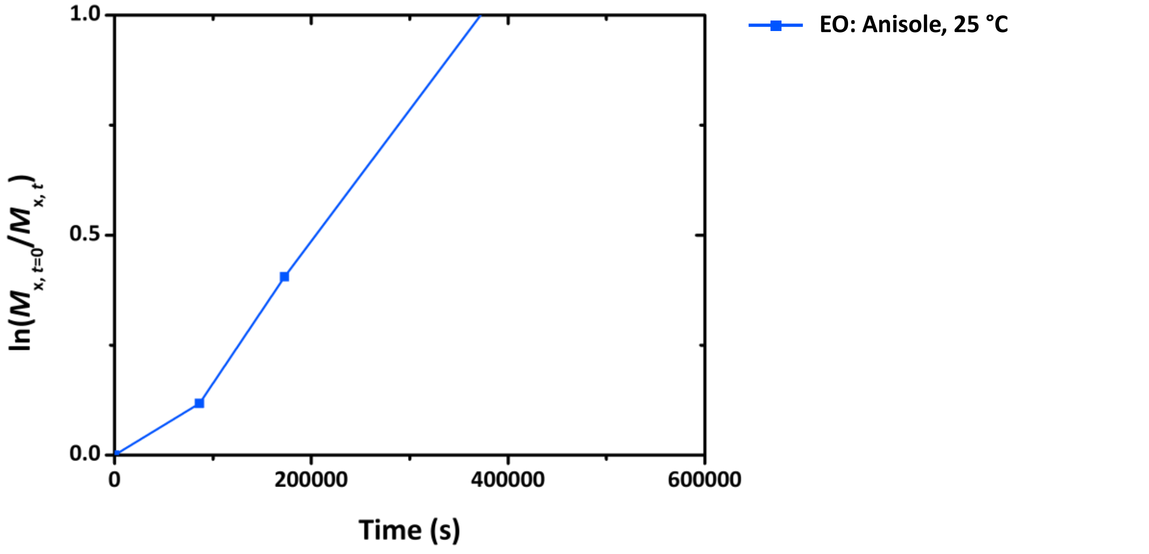

Figure S64: Enlargement of the pseudo-first-order plot of the copolymerization of EO with PO in Anisole at 25 °C obtained by offline kinetics. The enlarged start period shows the induction of the EO signal. The PO graph is omitted for clarity reasons.

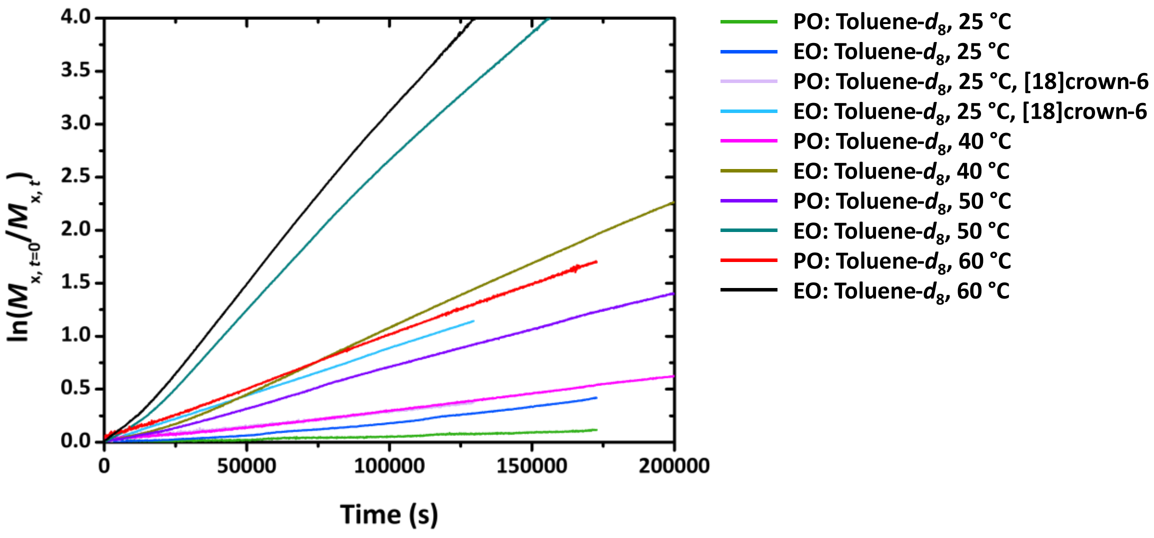

Figure S65: Pseudo-first-order plot of the copolymerization of EO with PO in Toluene-d_8 at different conditions obtained by ^{1}H NMR kinetics.

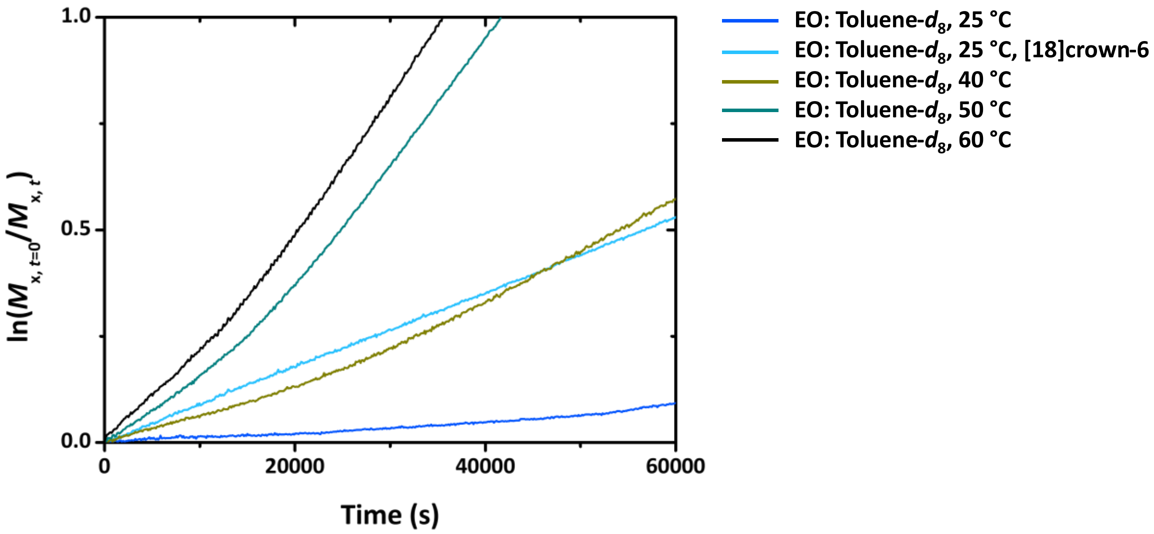

Figure S66: Enlargement of the pseudo-first-order plot of the copolymerization of EO with PO in Toluene-d_8 at different conditions obtained by ^{1}H NMR kinetics. The enlarged start period shows the induction of the EO signal. The PO graphs are omitted for clarity reasons.

Table S1: Apparent propagation constant of EO in the copolymerization ($k_{app, copo}$) from the ^{1}H NMR kinetics.

Solvent	T / °C	$k_{app, copo}$ (EO) / (10^{-4} s^{-1})
DMSO-d_6	25[a]	3.63 ± 0.01
DMSO-d_6	25	2.37 ± 0.01
DMSO-d_6	40	6.53 ± 0.03
Anisole	25[a]	0.0741 ± 0.0001
Anisole	25	0.0305 ± 0.0003
Anisole	40	0.103 ± 0.001
Anisole	60	0.899 ± 0.002
Toluene-d_8	25[a]	0.0885 ± 0.0001
Toluene-d_8	25	0.0334 ± 0.0001
Toluene-d_8	40	0.116 ± 0.001
Toluene-d_8	50	0.260 ± 0.001
Toluene-d_8	60	0.327 ± 0.001

[a]2 eq. [18]crown-6 per potassium.

Size exclusion chromatography

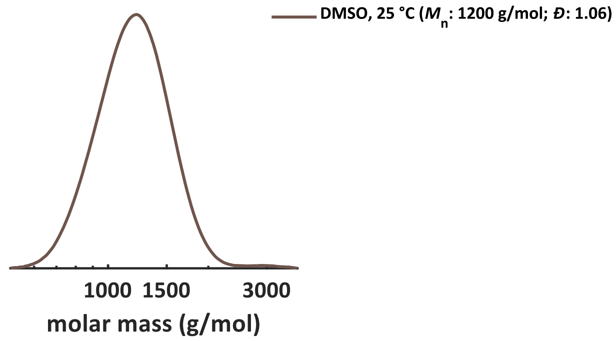

Figure S67: SEC of the copolymer obtained after ^{1}H NMR kinetics (eluent: DMF with 1 g/L LiBr, RI detector, calibration: PEG). Reaction conditions are in the graph's description.

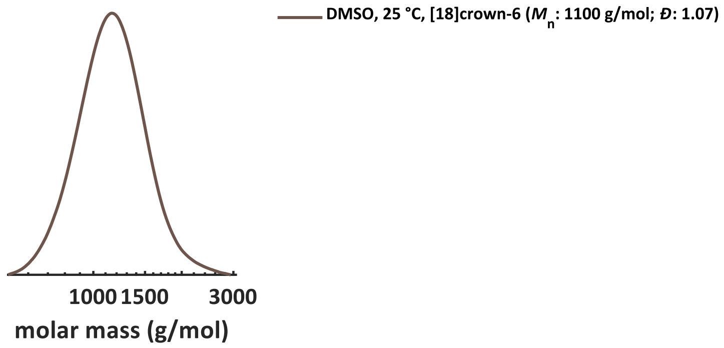

Figure S68: SEC of the copolymer obtained after ^{1}H NMR kinetics (eluent: DMF with 1 g/L LiBr, RI detector, calibration: PEG). Reaction conditions are in the graph's description.

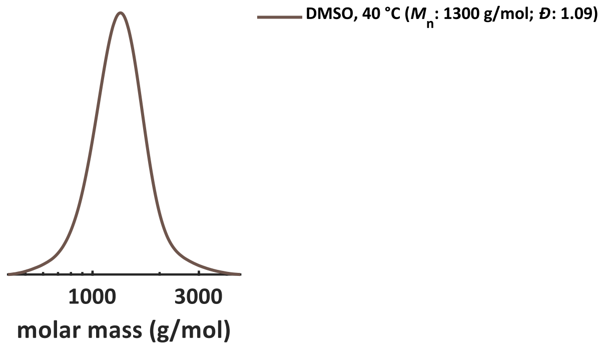

Figure S69: SEC of the copolymer obtained after ^{1}H NMR kinetics (eluent: DMF with 1 g/L LiBr, RI detector, calibration: PEG). Reaction conditions are in the graph's description.

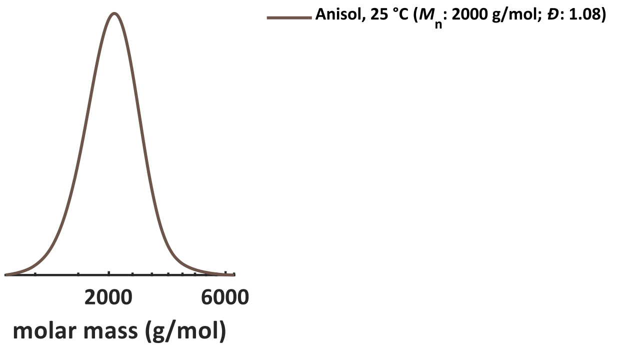

Figure S70: SEC of the copolymer obtained after ^{1}H NMR kinetics (eluent: DMF with 1 g/L LiBr, RI detector, calibration: PEG). Reaction conditions are in the graph's description.

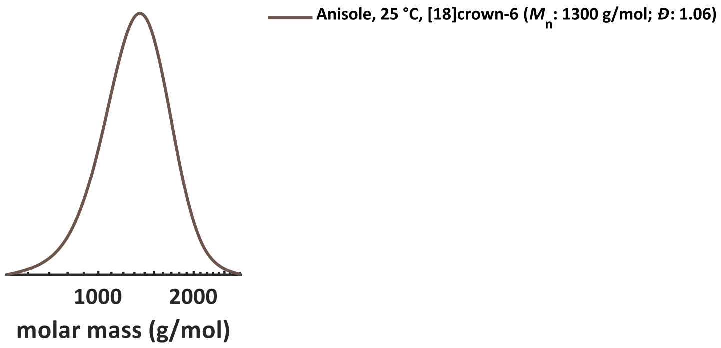

Figure S71: SEC of the copolymer obtained after ^{1}H NMR kinetics (eluent: DMF with 1 g/L LiBr, RI detector, calibration: PEG). Reaction conditions are in the graph's description.

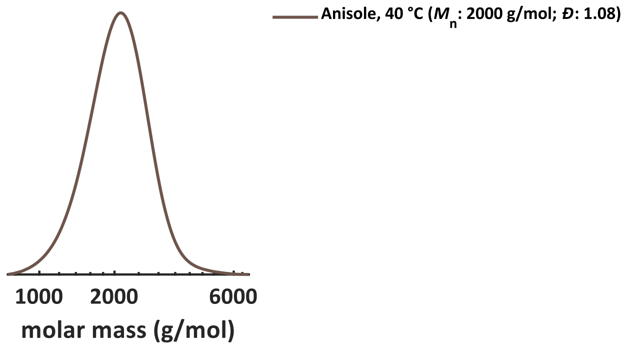

Figure S72: SEC of the copolymer obtained after ^{1}H NMR kinetics (eluent: DMF with 1 g/L LiBr, RI detector, calibration: PEG). Reaction conditions are in the graph's description.

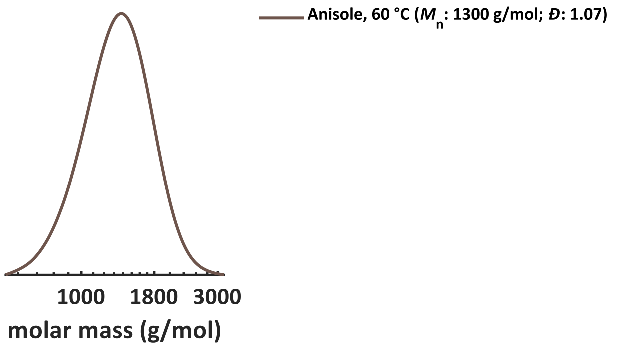

Figure S73: SEC of the copolymer obtained after ^{1}H NMR kinetics (eluent: DMF with 1 g/L LiBr, RI detector, calibration: PEG). Reaction conditions are in the graph's description.

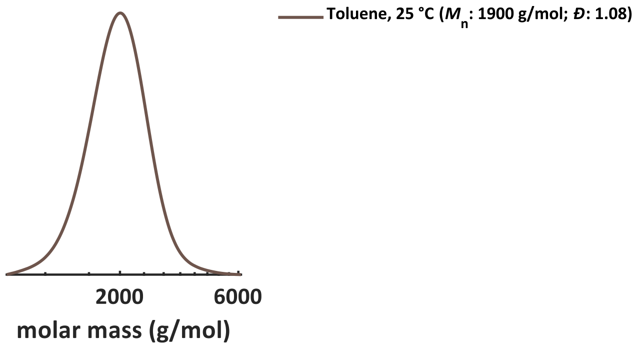

Figure S74: SEC of the copolymer obtained after ^{1}H NMR kinetics (eluent: DMF with 1 g/L LiBr, RI detector, calibration: PEG). Reaction conditions are in the graph's description.

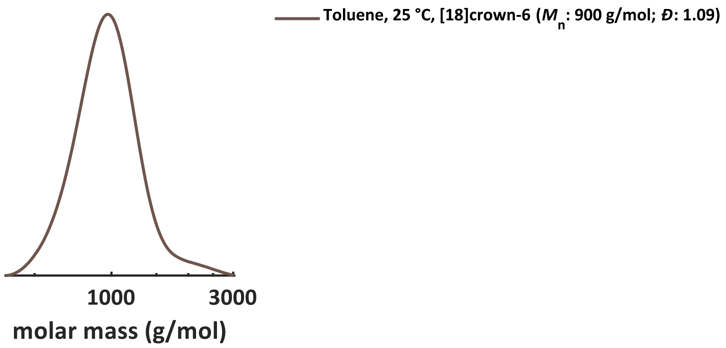

Figure S75: SEC of the copolymer obtained after ^{1}H NMR kinetics (eluent: DMF with 1 g/L LiBr, RI detector, calibration: PEG). Reaction conditions are in the graph's description.

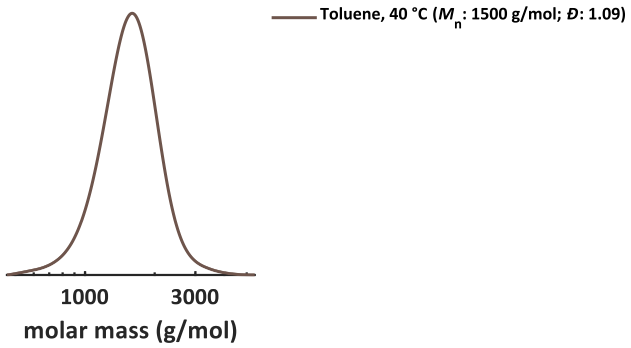

Figure S76: SEC of the copolymer obtained after ^{1}H NMR kinetics (eluent: DMF with 1 g/L LiBr, RI detector, calibration: PEG). Reaction conditions are in the graph's description.

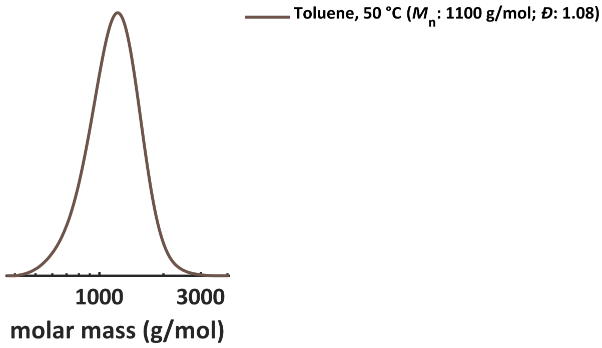

Figure S77: SEC of the copolymer obtained after ^{1}H NMR kinetics (eluent: DMF with 1 g/L LiBr, RI detector, calibration: PEG). Reaction conditions are in the graph's description.

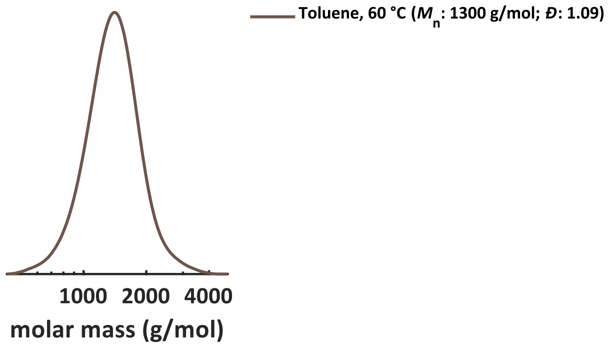

Figure S78: SEC of the copolymer obtained after ^{1}H NMR kinetics (eluent: DMF with 1 g/L LiBr, RI detector, calibration: PEG). Reaction conditions are in the graph's description.

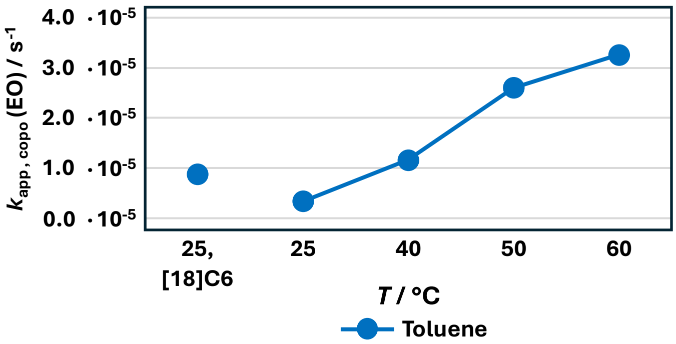

Figure S79: Propagation constant of EO in the copolymerization with PO. Please note that this does not equal a propagation constant k_p of a homopolymerization.

^{1}H NMR Spectroscopy

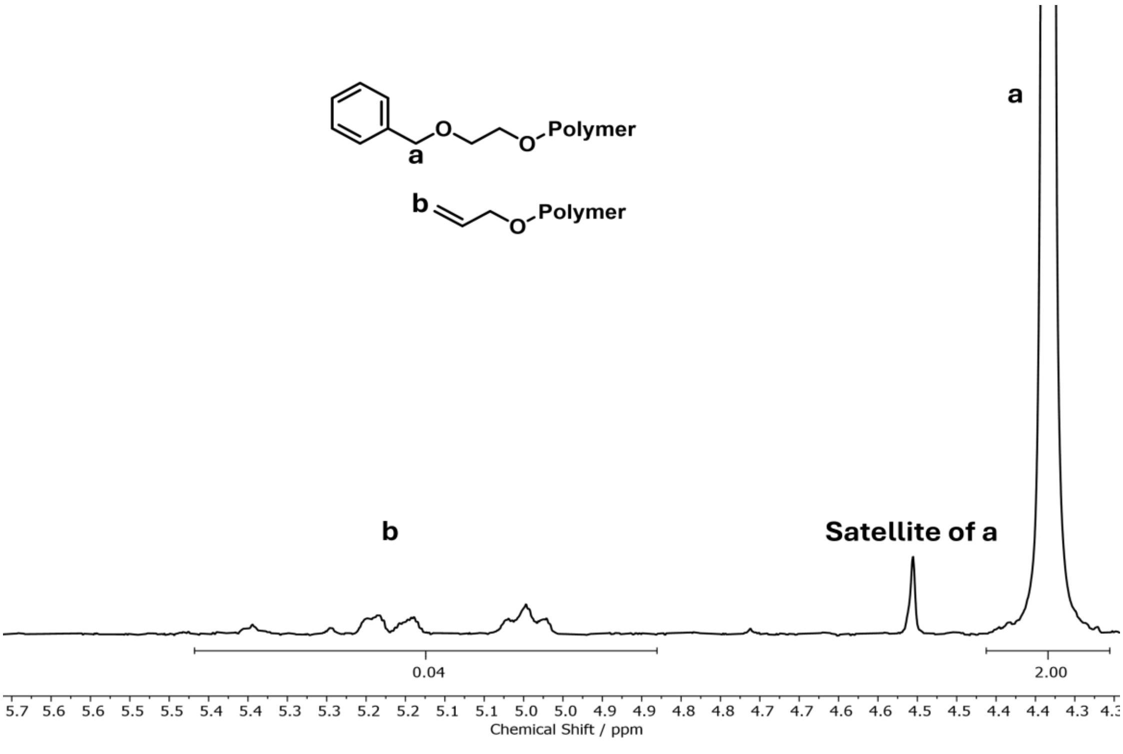

Figure S80: Section of the ^{1}H NMR Spectrum (400 MHz, Toluene-d_8) of the P(EO-*co*-PO) copolymer obtained in toluene-d_8 at 40 °C used for the calculation of the ratio of proton abstraction in relation to desired initiation.

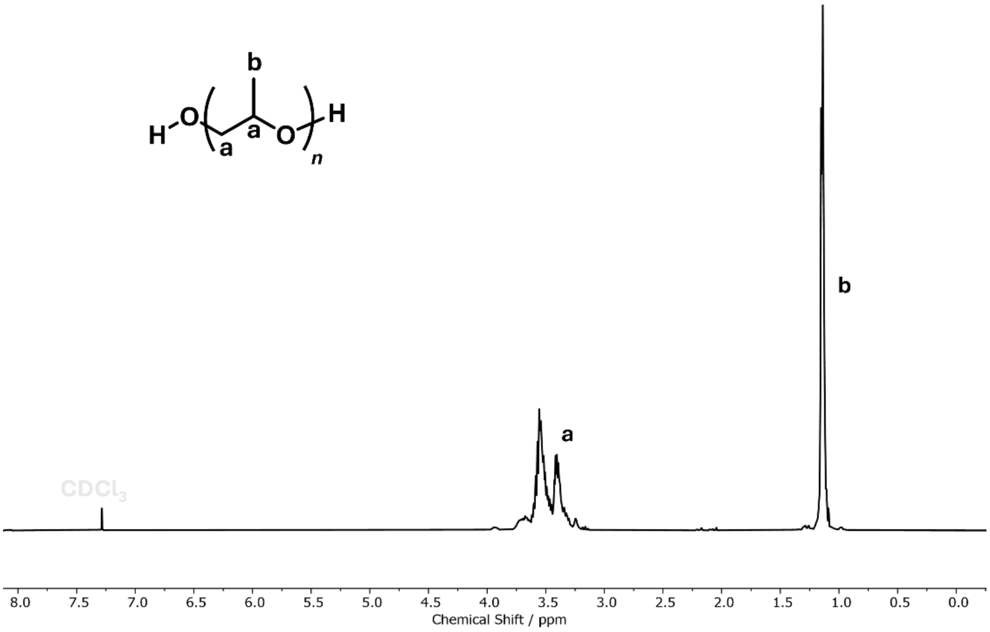

Figure S 81: ^{1}H NMR Spectrum (400 MHz, $CDCl_3$) of the commercial PPO homopolymer obtained from Merck KGaA.

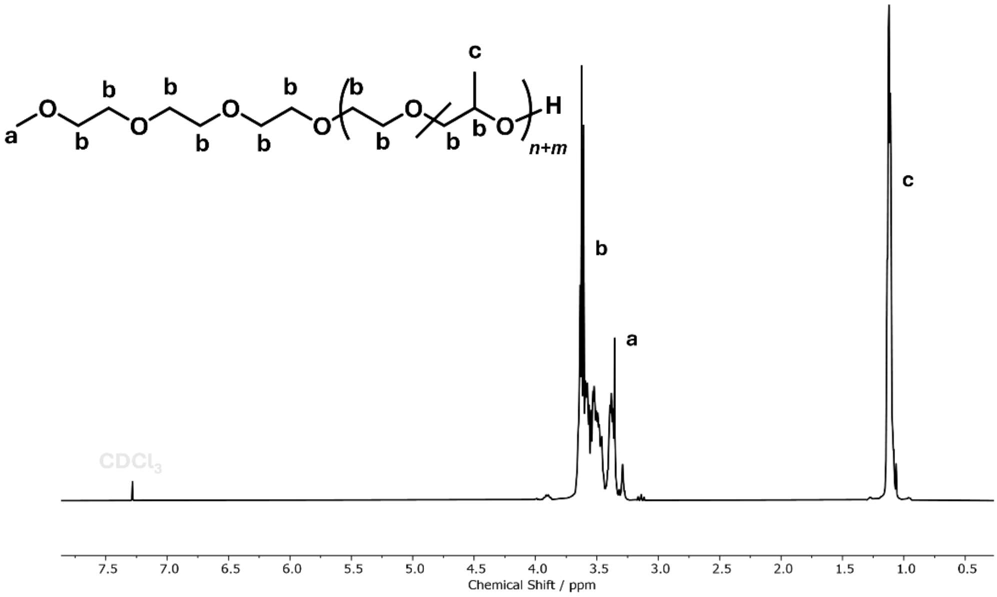

Figure S 82: ^{1}H NMR Spectrum (400 MHz, $CDCl_3$) of the P(EO-*co*-PO) copolymer obtained in toluene at 40 °C with 35 mol% of EO.

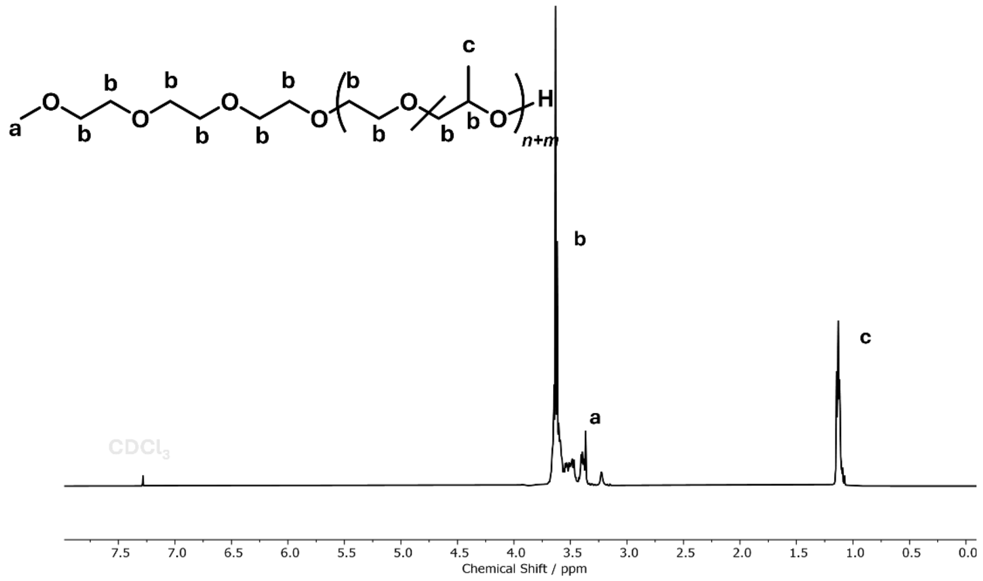

Figure S 83: ^{1}H NMR Spectrum (400 MHz, $CDCl_3$) of the P(EO-*co*-PO) copolymer obtained in toluene at 40 °C with 55 mol% of EO.

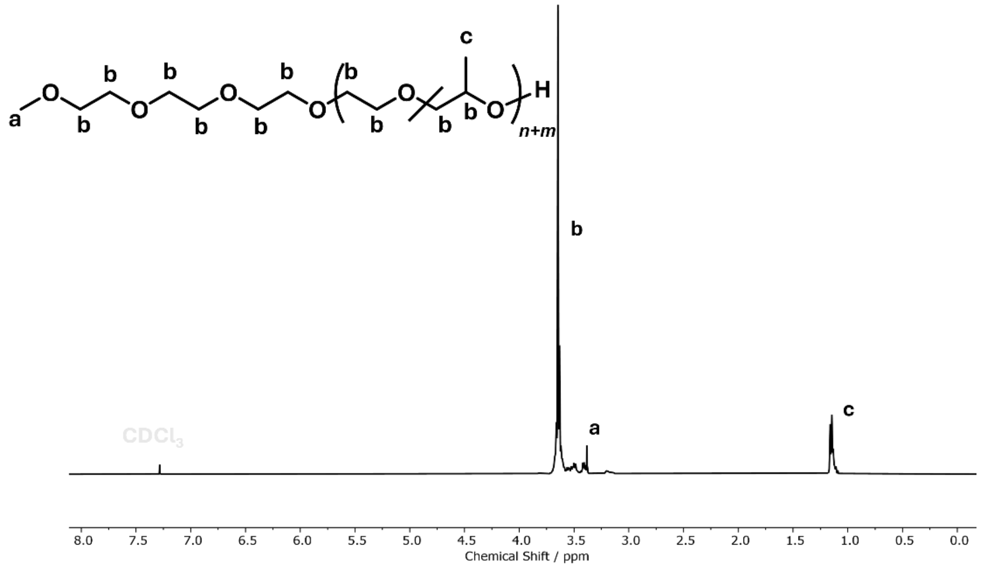

Figure S 84: ^{1}H NMR Spectrum (400 MHz, $CDCl_3$) of the P(EO-*co*-PO) copolymer obtained in toluene at 40 °C with 70 mol% of EO

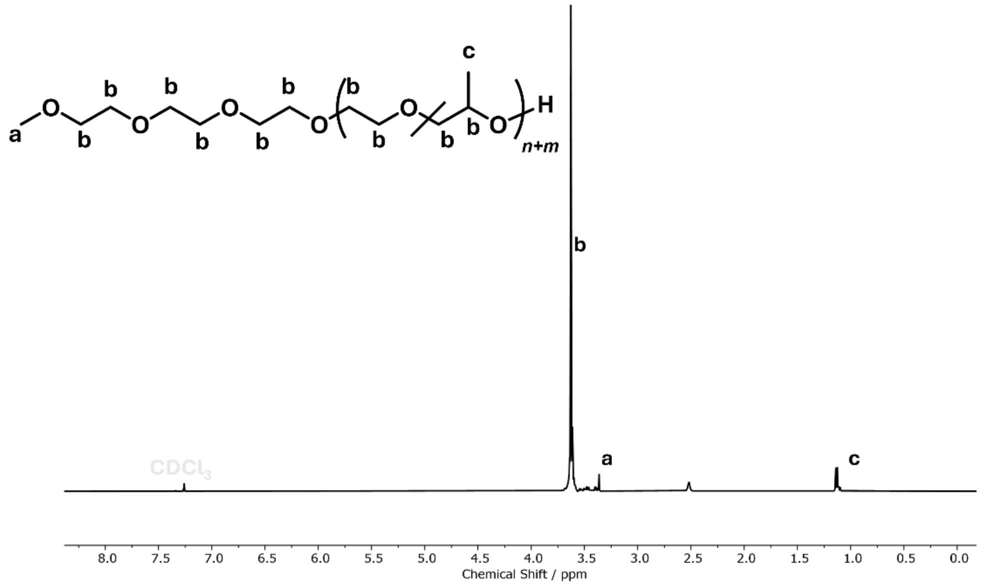

Figure S 85: ^{1}H NMR Spectrum (400 MHz, $CDCl_3$) of the P(EO-*co*-PO) copolymer obtained in toluene at 40 °C with 90 mol% of EO

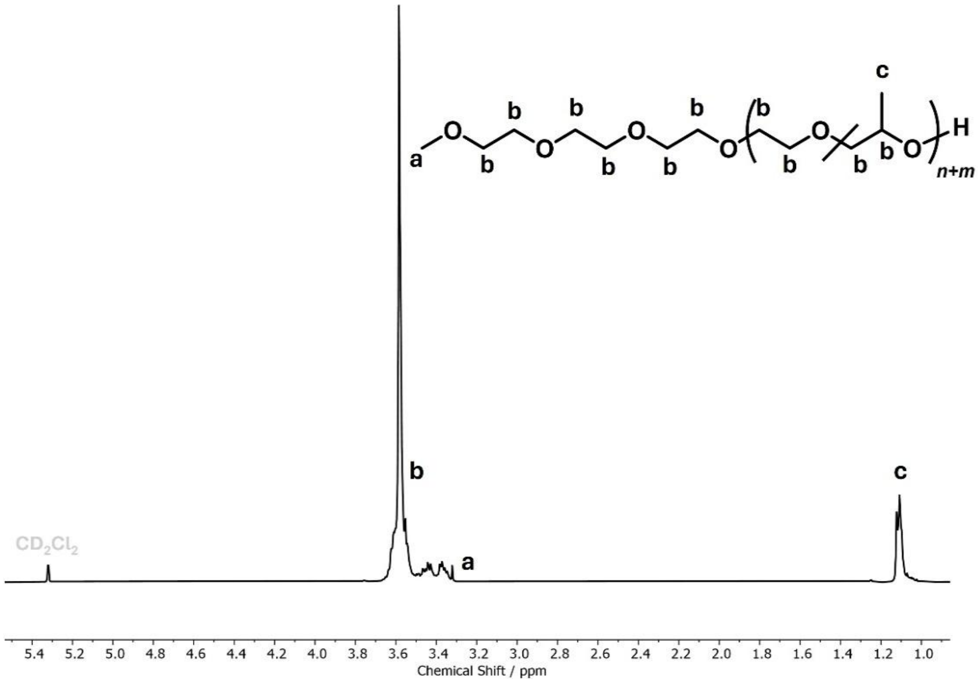

Figure S86: ^{1}H NMR Spectrum (400 MHz, CD_2Cl_2) of the P(EO-*co*-PO) copolymer obtained in toluene at 40 °C.

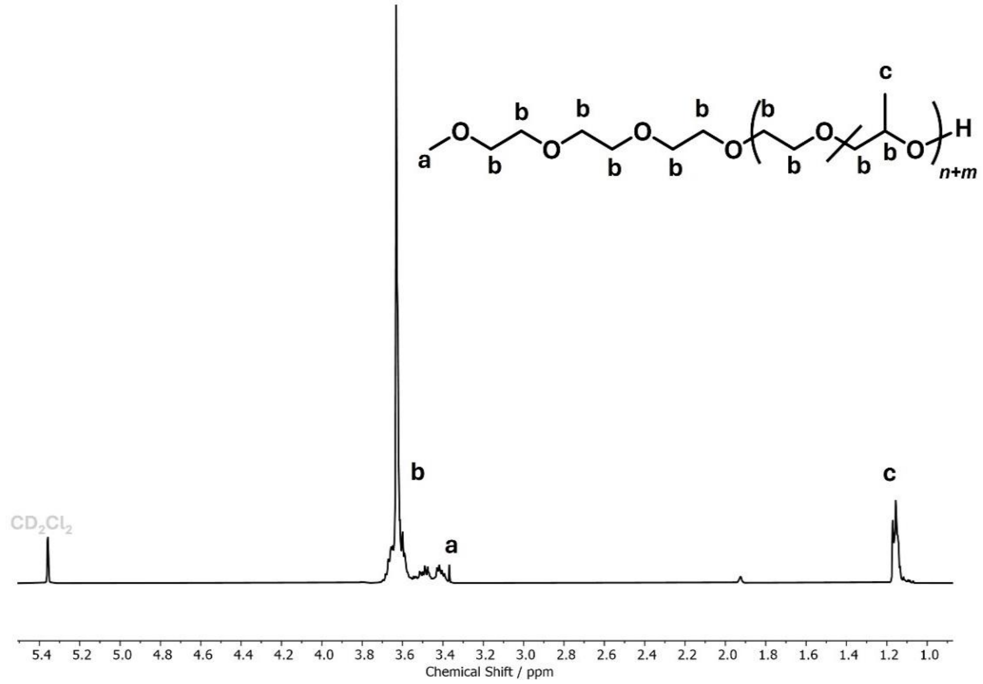

Figure S87: ^{1}H NMR Spectrum (400 MHz, CD_2Cl_2) of the P(EO-*co*-PO) copolymer obtained in toluene at 50 °C.

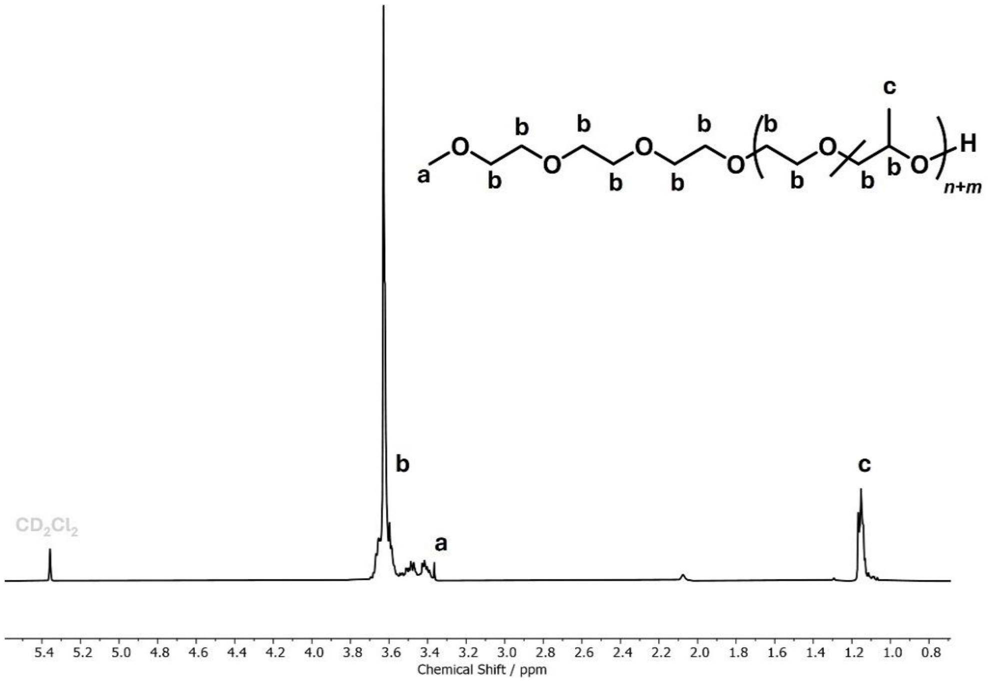

Figure S88: ^{1}H NMR Spectrum (400 MHz, CD_2Cl_2) of the P(EO-*co*-PO) copolymer obtained in toluene at 60 °C.

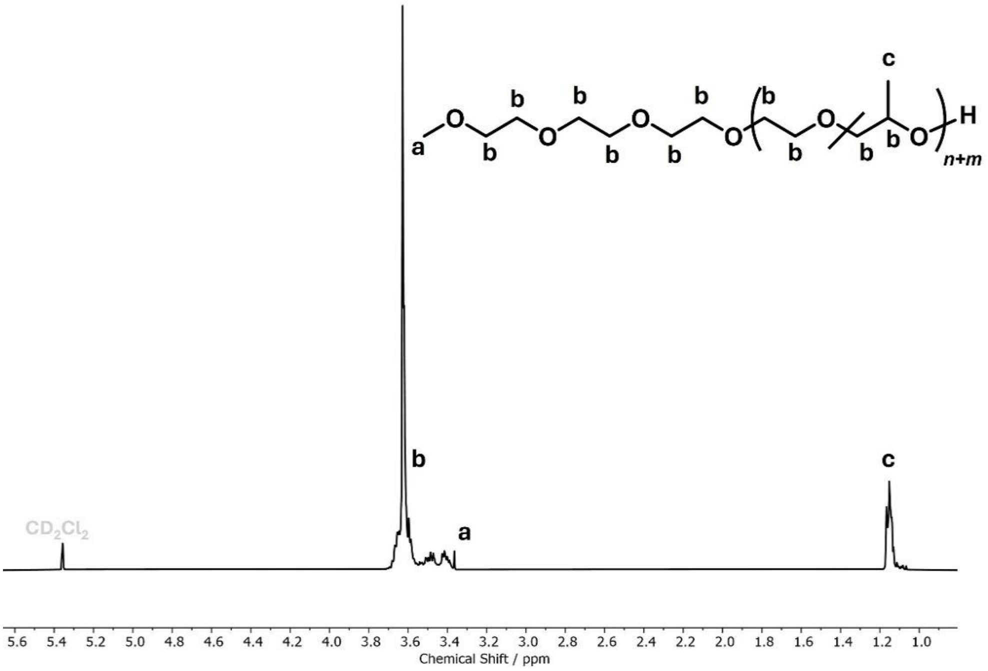

Figure S89: ^{1}H NMR Spectrum (400 MHz, CD_2Cl_2) of the P(EO-*co*-PO) copolymer obtained in toluene at 25 °C with addition of [18]C6.

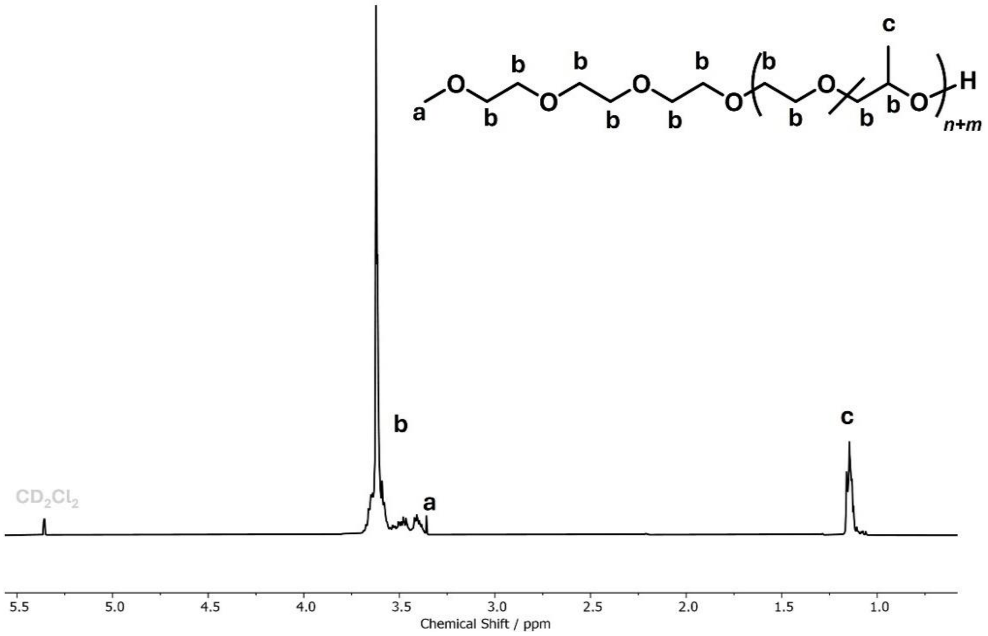

Figure S90: ^{1}H NMR Spectrum (400 MHz, CD_2Cl_2) of the P(EO-*co*-PO) copolymer obtained in anisole at 40 °C.

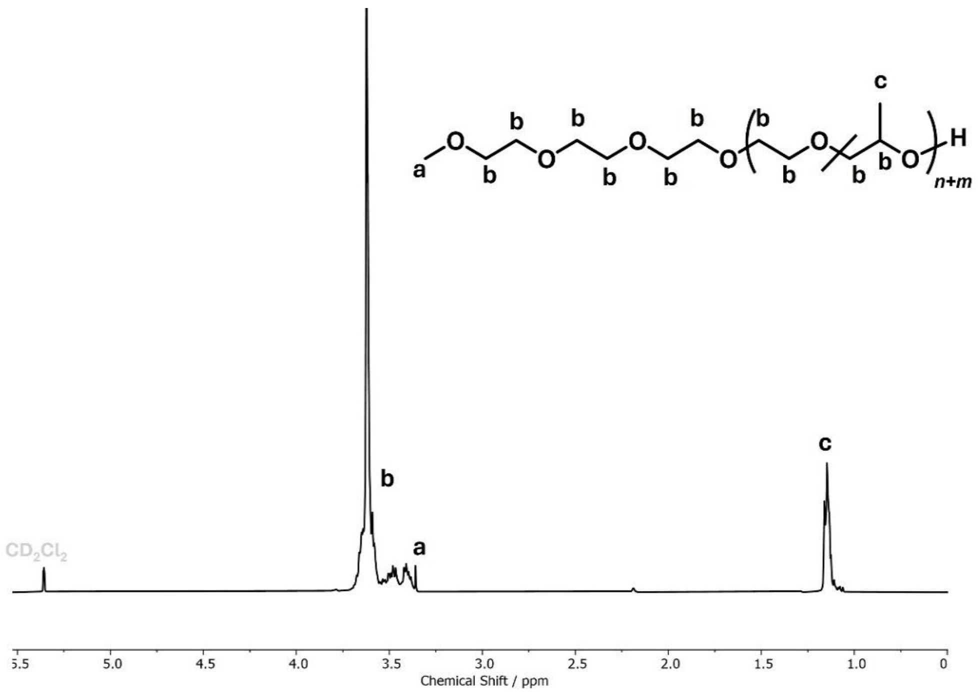

Figure S91: ^{1}H NMR Spectrum (400 MHz, CD_2Cl_2) of the P(EO-*co*-PO) copolymer obtained in DMSO at 40 °C.

Size exclusion chromatography

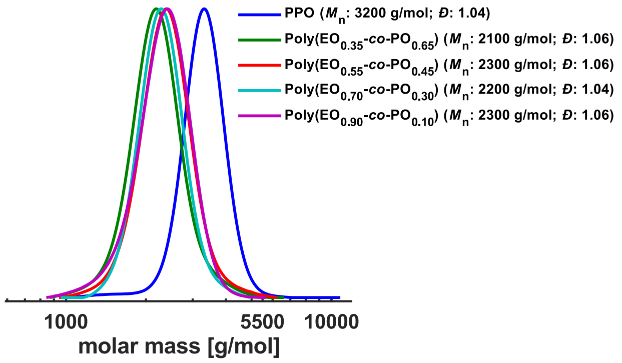

Figure S 92: SECs of all polymers synthesized for the T_{cp} measurements in correlation to the mol%$_{EO}$. RI-Detector, PEG calibration, eluent: DMF.

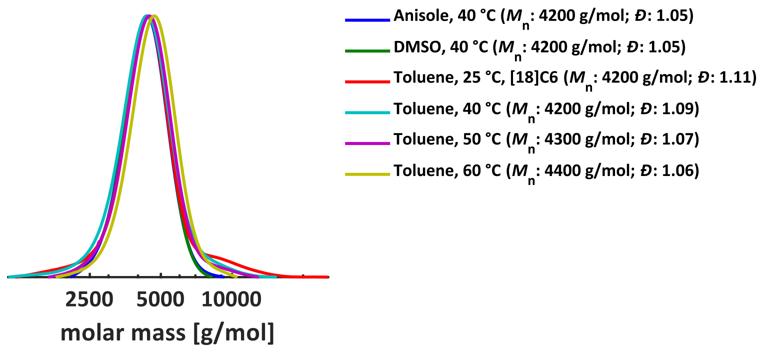

Figure S93: SECs of all polymers synthesized for turbidimetry and DSC measurements. The legend describes the synthesis conditions, with a degree of deprotonation of 90%. RI-Detector, PEG calibration, eluent: DMF.

MALDI-ToF MS Spectrometry

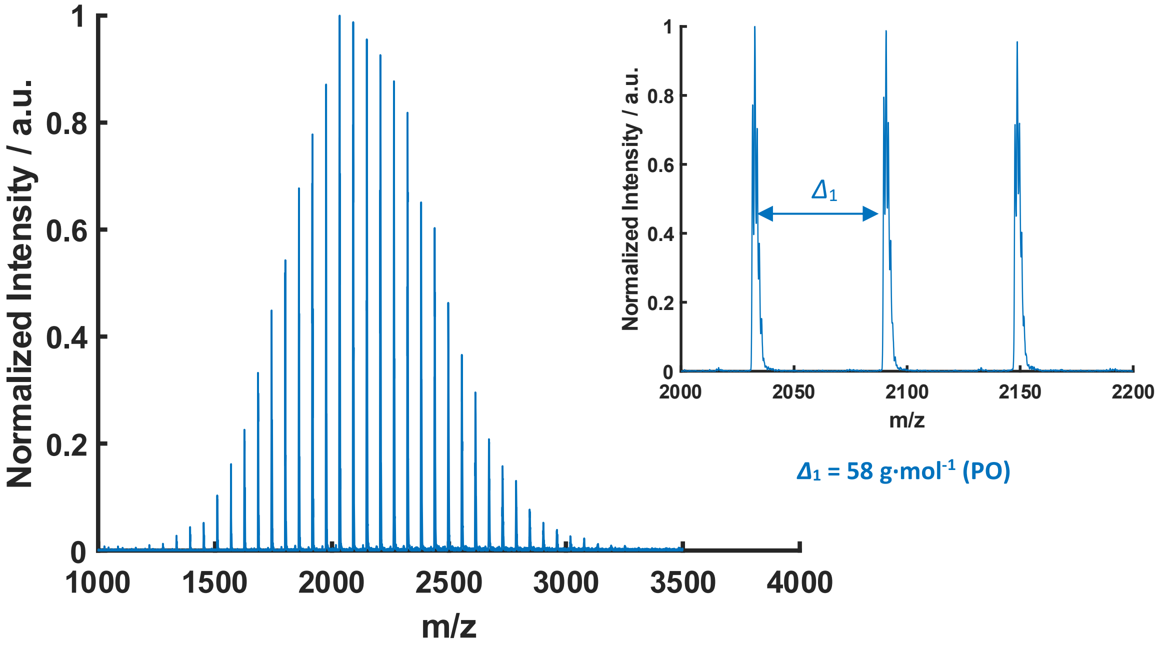

Figure S 94: MALDI-ToF MS of the commercial PPO homopolymer obtained from Merck KGaA.

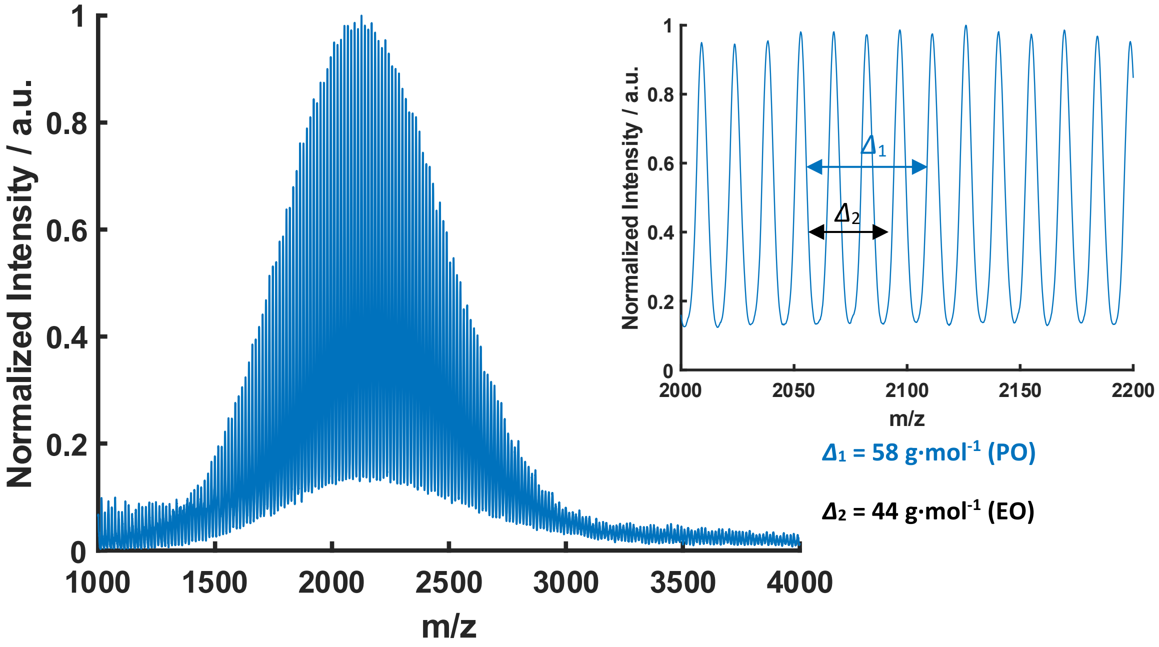

Figure S 95: MALDI-ToF MS of the P(EO-*co*-PO) copolymer obtained in toluene at 40 °C with 35 mol% of EO.

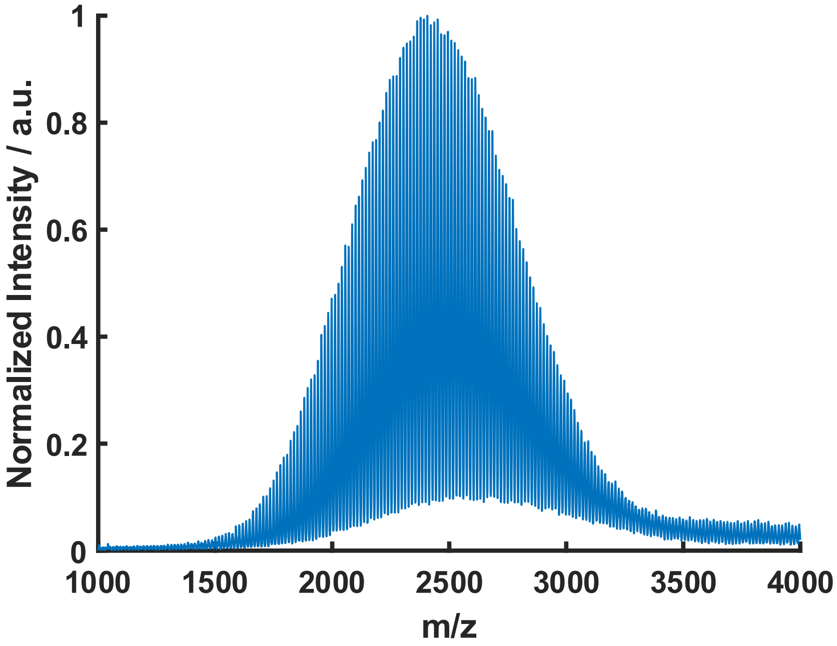

Figure S 96: MALDI-ToF MS of the P(EO-*co*-PO) copolymer obtained in toluene at 40 °C with 55 mol% of EO.

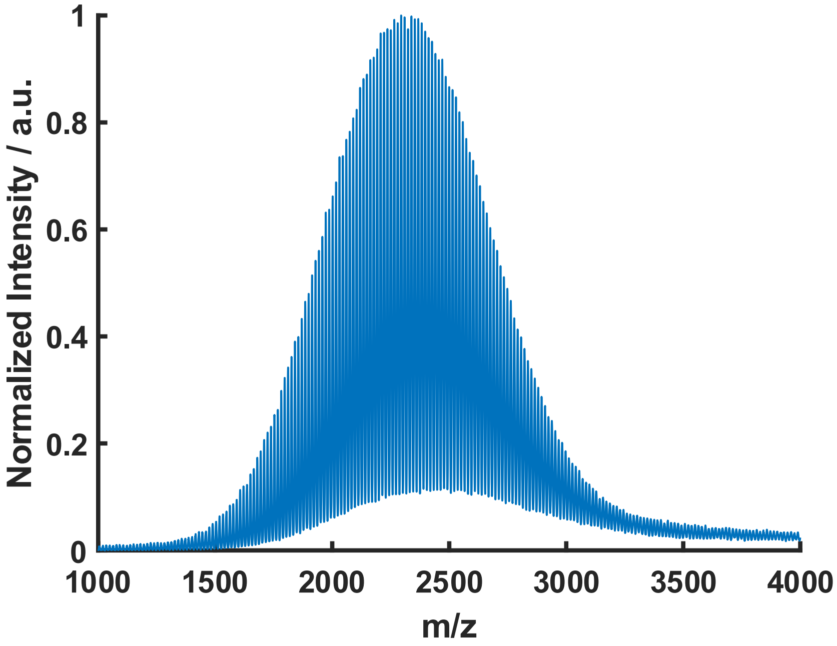

Figure S 97: MALDI-ToF MS of the P(EO-*co*-PO) copolymer obtained in toluene at 40 °C with 70 mol% of EO.

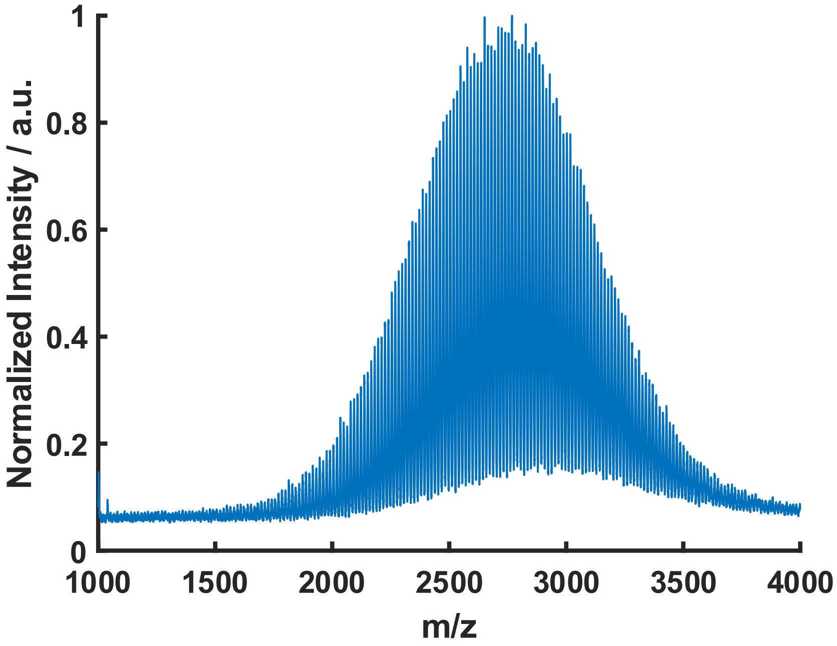

Figure S 98: MALDI-ToF MS of the P(EO-*co*-PO) copolymer obtained in toluene at 40 °C with 90 mol% of EO.

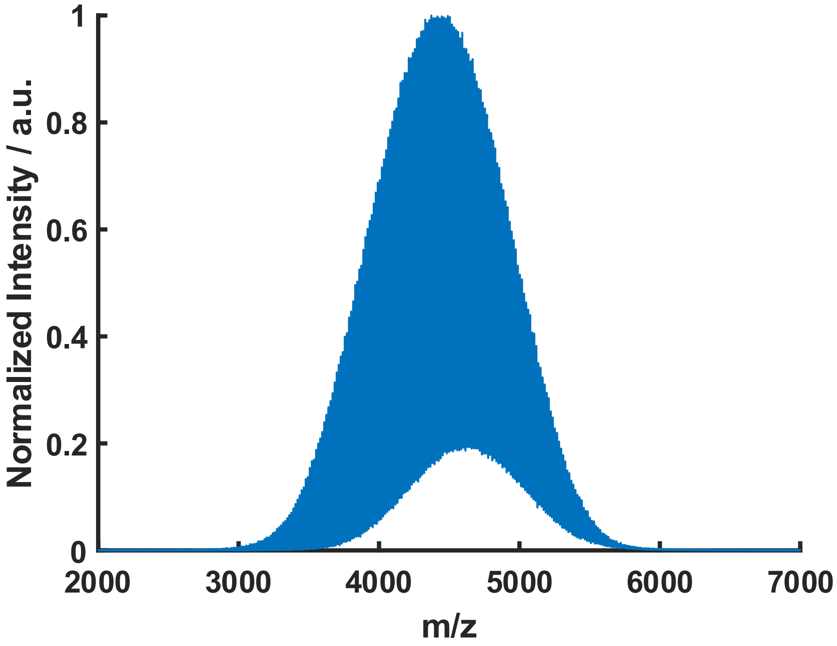

Figure S99: MALDI-ToF MS of the P(EO-*co*-PO) copolymer obtained in toluene at 40 °C.

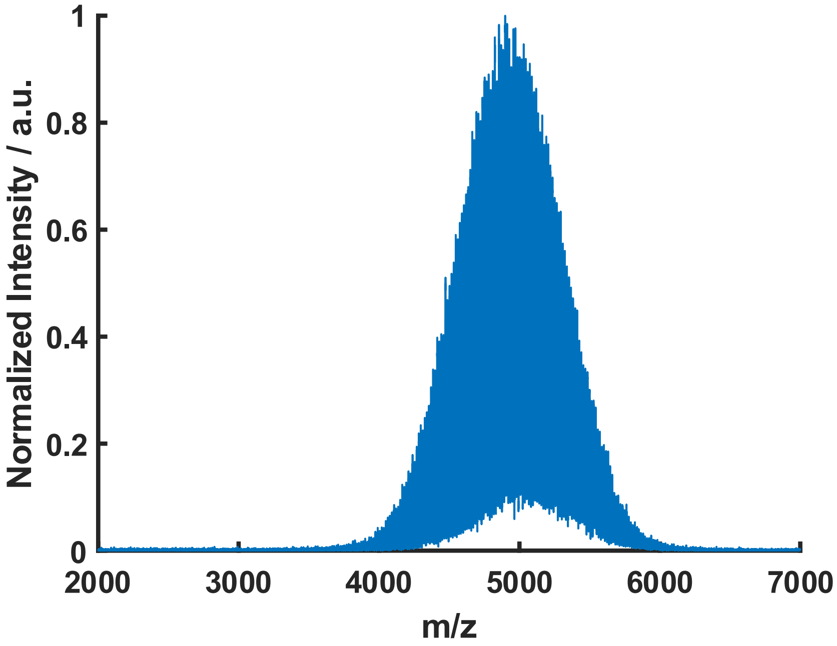

Figure S100: MALDI-ToF MS of the P(EO-*co*-PO) copolymer obtained in toluene at 50 °C.

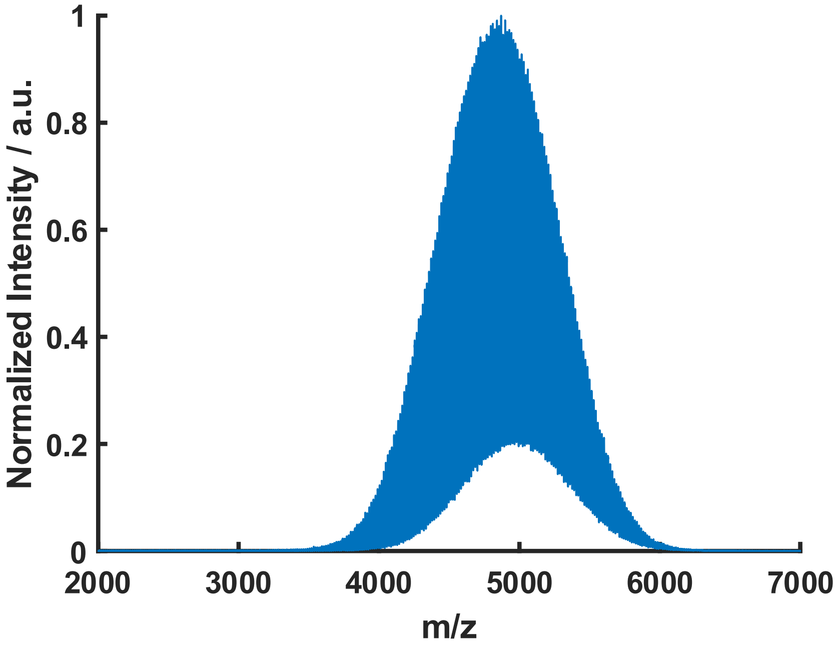

Figure S101: MALDI-ToF MS of the P(EO-*co*-PO) copolymer obtained in toluene at 60 °C.

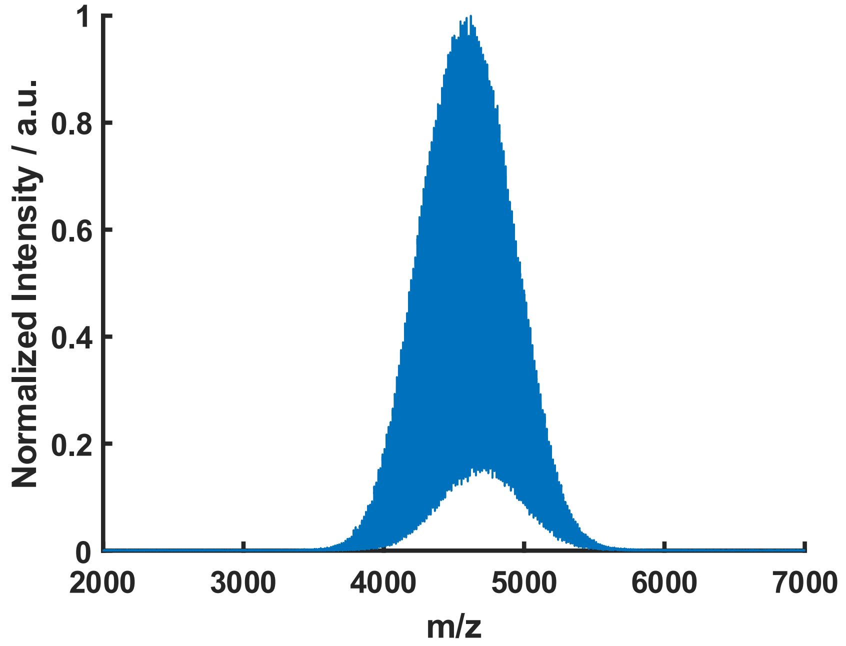

Figure S102: MALDI-ToF MS of the P(EO-*co*-PO) copolymer obtained in toluene at 25 °C with [18]C6.

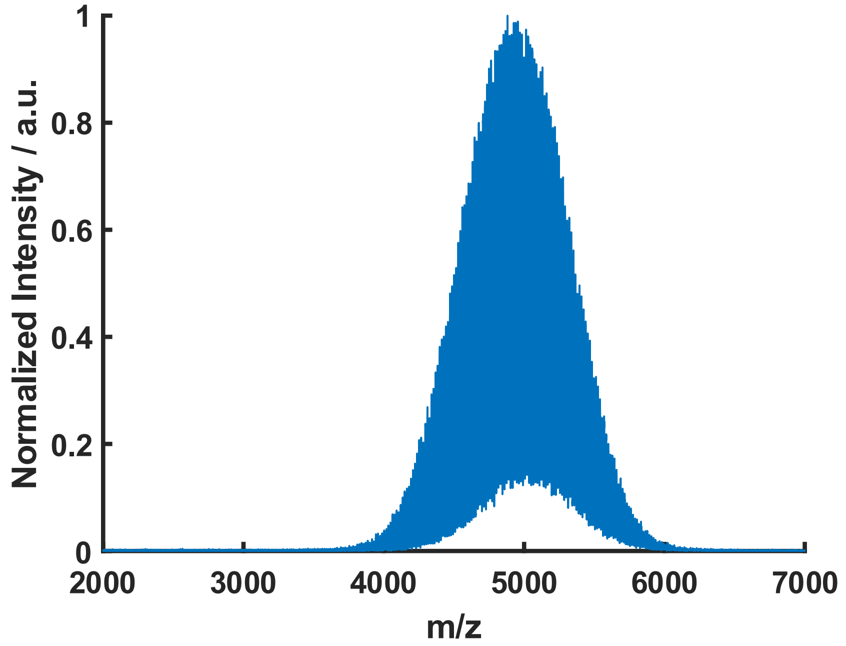

Figure S103: MALDI-ToF MS of the P(EO-*co*-PO) copolymer obtained in anisole at 40 °C.

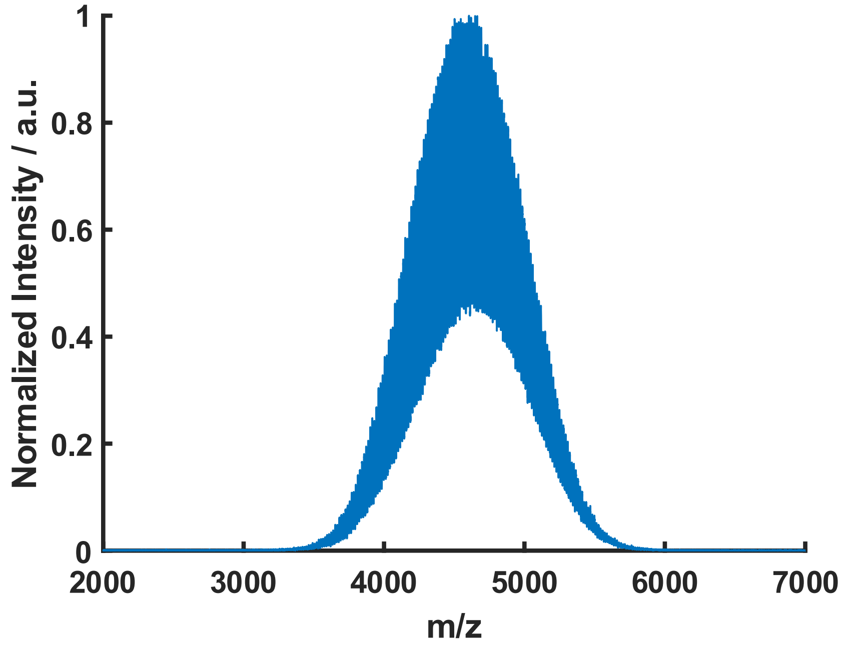

Figure S104: MALDI-ToF MS of the P(EO-*co*-PO) copolymer obtained in DMSO at 40 °C.

Turbidimetry measurements

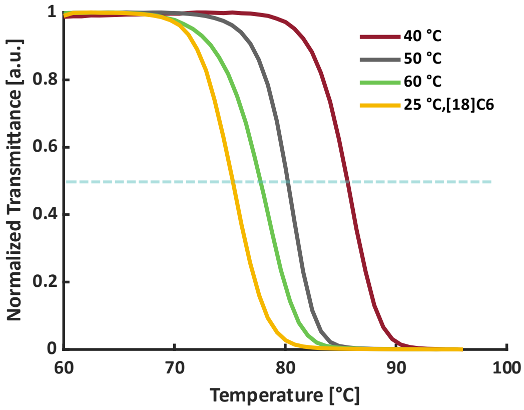

Figure S105: Results of the turbidimetry measurements of the polymers obtained from toluene at 25 °C with [18]C6, 40 °C, 50 °C, and 60 °C.

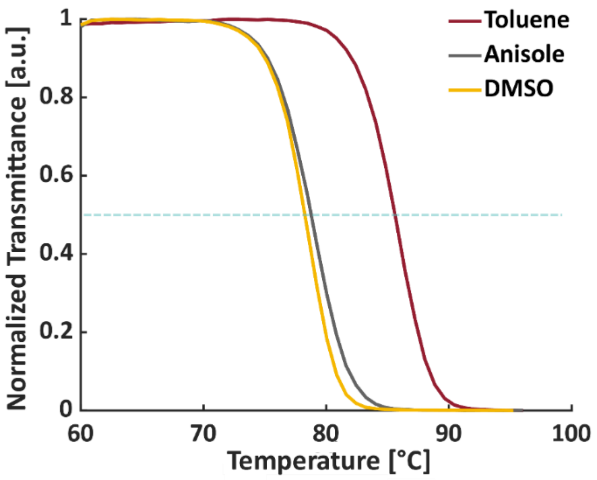

Figure S106: Results of the turbidimetry measurements of the polymers obtained at 40 °C in toluene, anisole, and DMSO.

DSC measurements

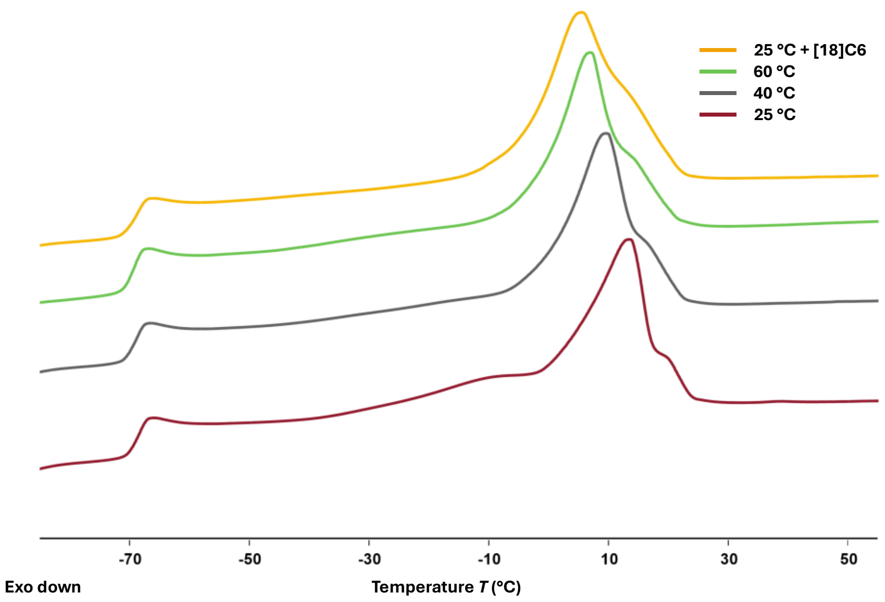

Figure S107: Results of the DSC measurements of the polymers obtained from toluene at 25 °C with [18]C6, 40 °C, 50 °C, and 60 °C.

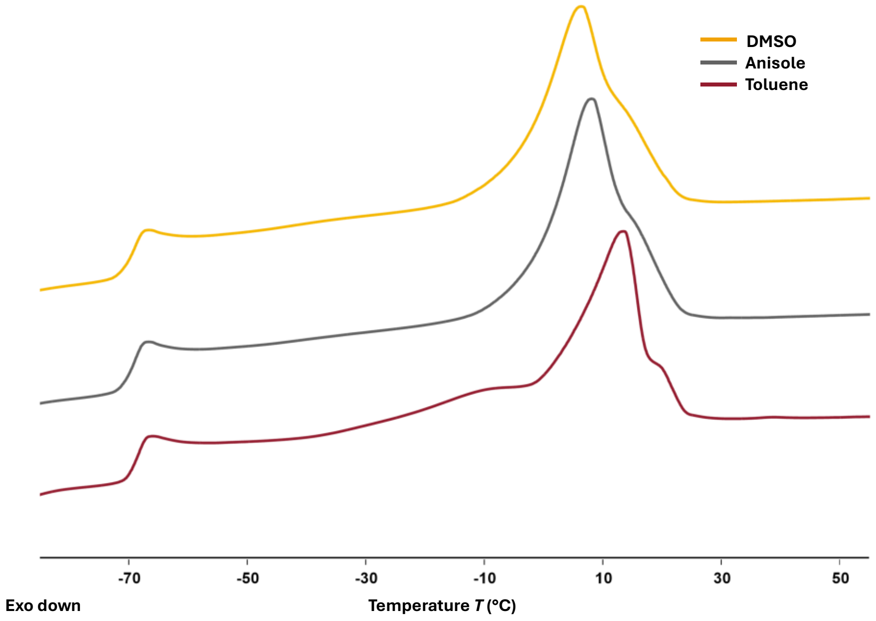

Figure S108: Results of the DSC measurements of the polymers obtained at 40 °C in toluene, anisole, and DMSO.

Table S2: Summary of obtained reactivity ratios, turbidimetry measurement results and DSC messurement results for the analyzed polymerization parameters.

Solvent	T [°C]	r_1 (PO)	r_2 (EO)	Δr[b]	T_{cp} [°C][c]	T_m [°C][d]	ΔH [J/g][e]
Toluene[a]	25	0.31	3.05	2.74	76	5	38
Toluene	40	0.26	3.78	3.52	86	13	42
Toluene	50	0.28	3.62	3.34	80	10	40
Toluene	60	0.31	3.21	2.90	78	7	39
Anisole	40	0.28	3.52	3.24	79	8	39
DMSO	40	0.32	3.10	2.78	78	6	37

[a]2 eq. [18]crown-6 per potassium. [b]$\Delta r = r_2 - r_1$. [c]Cloud point temperature determined via turbidimetry for c = 5 mg·ml^{-1}. [d]Melting temperature. [e]Melting enthalpy.

References

(1) Yu, G.-E.; Heatley, F.; Booth, C.; Blease, T. G. Anionic copolymerisation of ethylene oxide and propylene oxide. Investigation of double-bond content by NMR spectroscopy. *Eur. Polym. J.* **1995**, *31* (6), 589–593. DOI: 10.1016/0014-3057(94)00210-X.

(2) Steube, M.; Johann, T.; Plank, M.; Tjaberings, S.; Gröschel, A. H.; Gallei, M.; Frey, H.; Müller, A. H. E. Kinetics of Anionic Living Copolymerization of Isoprene and Styrene Using in Situ NIR Spectroscopy: Temperature Effects on Monomer Sequence and Morphology. *Macromolecules* **2019**, *52* (23), 9299–9310. DOI: 10.1021/acs.macromol.9b01790.

(3) Meyer, V. E.; Lowry, G. G. Integral and differential binary copolymerization equations. *J. Polym. Sci. A Gen. Pap.* **1965**, *3* (8), 2843–2851. DOI: 10.1002/pol.1965.100030811.
